# Basketball team optimization algorithm (BTOA): a novel sport-inspired meta-heuristic optimizer for engineering applications

**DOI:** 10.1038/s41598-025-05477-0

**Published:** 2025-07-01

**Authors:** Yujie Chen, Guangyu Wang, Baichuan Yin, Chongyun Ma, Zhiqiao Wu, Ming Gao

**Affiliations:** 1 School of Statistics, Dongbei University of Finance and Economics, Dalian, Liaoning China; 2 School of Data Science and Artificial Intelligence, Dongbei University of Finance and Economics, Dalian, Liaoning China; 3 School of Finance, Dongbei University of Finance and Economics, Dalian, Liaoning China; 4 School of Management Science and Engineering, Key Laboratory of Liaoning Province for Data Analytics and Decision-Making Optimization, Dongbei University of Finance and Economics, Dalian, Liaoning China

**Keywords:** Metaheuristic algorithms, Optimization, Dynamic positioning strategy, UAV path planning, Engineering applications, Machine learning, Civil engineering, Mechanical engineering, Computational science, Statistics

## Abstract

Real-world optimisation problems are increasingly high-dimensional, nonlinear and constrained. The No Free Lunch theorem implies that no single optimiser dominates across all problem classes, making domain-specific metaheuristics indispensable. Yet mainstream population-based methods often converge prematurely and fail to balance exploration and exploitation under such complexity. To address these limitations, we propose the Basketball Team Optimisation Algorithm (BTOA), a sports-inspired metaheuristic. BTOA maps four basketball concepts-high-intensity training, fast breaks, dynamic positioning and coordinated passing-onto cooperative search operators. In addition, we introduce two extensible modules: (i)a dynamic positioning strategy guided by diagonal structures, significantly improving global exploration capabilities, and (ii) a VariableAttributes to manage the distribution of individual diversity. These modules can be embedded into other population-based optimisers, enriching the heuristic design space. Extensive experiments on the CEC2005 and CEC2017 benchmark suites with 30, 50 and 100 dimensions show that BTOA attains the lowest mean error on 82.61% of the CEC2005 functions and on 66.67%, 63.3% and 66.67% of the CEC2017 functions, respectively. Wilcoxon signed-rank and Friedman tests confirm the statistical significance of these gains. Additional comparisons against several recently proposed algorithms and competition-winning algorithms further highlight BTOA’s consistent advantage. Beyond benchmark tests, BTOA performs well on real-world problems with complex constraints and large decision spaces, such as UAV path planning. Its principled design alleviates key shortcomings of existing metaheuristics and offers a scalable, reliable tool for contemporary engineering optimisation tasks.

## Introduction

Optimization problems are widely encountered across diverse fields such as image processing^1^, artificial intelligence^2^, automatic control^3^, aerospace^4^, biomedicine^5^, and production design^6^^7,8^. These problems typically require identifying the best solution from a set of feasible candidates under various constraints^9,10^. As the complexity and dimensionality of real-world problems grow, the demand for robust and efficient optimization algorithms becomes increasingly critical. Existing optimization methods can be broadly categorized into mathematical (deterministic) methods and metaheuristic (stochastic) methods^11^.

Classical mathematical optimization techniques, including the Nelder and Mead algorithm^12^, gradient descent method^13^, Hooke and Jeeves algorithm^14^, Lagrange multiplier method^15^, and Newton’s method^16^, are grounded in rigorous mathematical formulations. These methods perform well when objective functions are smooth, differentiable, and possess unique optima. However, they are often sensitive to initial conditions and struggle with non-convex, large-scale, and multimodal landscapes^17,18^.

To overcome these limitations, researchers have developed metaheuristic algorithms inspired by nature^19^, biology^20^, sports^21^, and mathematical logic^22^. These algorithms are typically gradient-free, population-based, and capable of handling high-dimensional, nonlinear, and multimodal optimization problems^23^. By combining randomness with adaptive search strategies, metaheuristics are well-suited for balancing global exploration and local exploitation^23,24^, and have been successfully applied to a variety of practical domains^25^.

Nevertheless, despite considerable advances, many metaheuristics still suffer from two major issues: premature convergence and loss of population diversity^26–29^. Maintaining an effective balance between exploration (searching new areas) and exploitation (refining known good areas) remains a persistent challenge^30–32^. According to the No Free Lunch (NFL) theorem^33^, no single algorithm performs best across all optimization problems. Due to their stochastic nature, metaheuristics may converge to suboptimal solutions, especially in cases where the problem structure is unknown or highly irregular^34,35^.

Moreover, different problems often align better with the search dynamics of specific algorithms. For example, the Ant Colony Optimization (ACO) algorithm^36^ is highly effective for discrete path planning but less suitable for continuous problems. The Electric Eel Foraging Optimization (EEFO) algorithm^34^ excels in hydropower control tasks, while the Artificial Protozoa Optimizer (APO)^31^ demonstrates strong performance in discrete image segmentation. Such domain-specific strengths indicate the value of designing new algorithms that generalize well across a wide range of problems while remaining customizable to specific scenarios^37–39^.

To further advance the development of more powerful algorithms and contribute reusable components to the heuristic optimization community, we propose a novel metaheuristic, Basketball Team Optimization Algorithm (BTOA), inspired by the cooperative strategies and dynamic behaviors observed in basketball teams. The algorithm captures four core behavioral mechanisms: high-intensity training, fast breaks, dynamic positioning, and boundary re-entry. Notably, BTOA introduces a Dynamic Positioning Strategy guided by diagonal structures to enhance global exploration, and an adaptivity factor that dynamically adjusts the balance between exploration and exploitation based on population ranking and the current iteration. These innovations are designed to mitigate premature convergence, maintain population diversity, and improve convergence accuracy in complex search spaces.

The major contributions of this study can be summarized as follows:

- *Novel heuristic algorithm* We propose a new optimization algorithm inspired by basketball players’ high-intensity training, fast break strategies, dynamic positioning, and return behaviors.
- *Dynamic positioning strategy* Introduces a dynamic positioning strategy guided by diagonal structures, enhancing global exploration capabilities.
- *Balancing diversity and adaptivity* Designed VariableAttributes to manage the distribution of individual diversity and a dynamic adaptivity factor to balance exploration and exploitation throughout iterations.
- *Comparative performance evaluation* Demonstrates significant advantages through extensive experiments on CEC2005 and CEC2017 benchmarks (30, 50, 100 dimensions) compared with six classical and four advanced algorithms, validated by Wilcoxon signed-rank and Friedman tests.
- *Real-world applicability* Shows effective performance in UAV path planning and six engineering problems, highlighting practical relevance.

## Literature review

Meta-heuristic algorithms have emerged as powerful tools for solving complex optimization problems across various domains. These algorithms are inspired by natural, social, and physical phenomena and can be broadly categorized into four groups: evolutionary algorithms, swarm intelligence algorithms, physics/mathematics-based algorithms, and human/social-based algorithms^40^. Each category encompasses a variety of algorithms with unique strategies and advantages, as shown in Table 1.

**Evolutionary Algorithms**

Evolutionary algorithms are inspired by the natural evolution process and principles of survival of the fittest. These algorithms simulate genetic evolution to perform search operations. A classic example is the Genetic Algorithm (GA)^41^, which uses selection, crossover, and mutation operations to evolve a population of solutions towards an optimal solution. Differential Evolution (DE)^42^ is another notable algorithm that enhances population diversity through differential operators. Other algorithms in this category include Evolution Strategy (ES)^43^, Memetic Algorithm (MA)^44^, Evolutionary Programming (EP)^45^, and Genetic Programming (GP)^46^. These algorithms effectively adapt and evolve solutions over successive generations, making them suitable for a wide range of optimization problems^47–50^.

**Swarm Intelligence Algorithms**

Swarm intelligence algorithms draw inspiration from the collective behavior of social organisms such as birds, fish, and insects. These algorithms simulate the decentralized, self-organizing nature of these organisms to perform complex tasks through simple interactions. Particle Swarm Optimization (PSO)^51^, inspired by the foraging behavior of birds, adjusts particle positions based on personal and global best positions. Ant Colony Optimization (ACO)^36^ mimics the pheromone trail-laying behavior of ants to find optimal paths. Additional swarm intelligence algorithms include Artificial Bee Colony (ABC)^52^, Firefly Algorithm (FA)^53^, Grey Wolf Optimizer (GWO)^20^, Harris Hawks Optimization (HHO)^54^, Chimp Optimization Algorithm (ChOA)^55^, Smell Agent Optimization (SAO)^35^, Reptile Search Algorithm (RSA)^56^, Beluga Whale Optimization (BWO)^57^, Aquila optimizer (AO)^58^, Artificial Protozoa Optimizer (APO)^31^ Crested Porcupine Optimizer (CPO)^59^, the Horned lizard optimization algorithms^60^ and Electric eel foraging optimization (EEFO)^34^. These algorithms leverage collective behavior and information sharing to explore and exploit the search space effectively.

**Physics/Mathematics-Based Algorithms**

Physics and mathematics-based algorithms utilize principles from physical laws and mathematical theories to guide the search process. Simulated Annealing (SA)^61^, inspired by the annealing process in metallurgy, explores the search space by probabilistically accepting worse solutions to escape local optima. Gravitational Search Algorithm (GSA)^62^ uses the law of gravity to attract agents towards optimal solutions. The Sine Cosine Algorithm (SCA)^63^ and Gradient-Based Optimizer (GBO)^64^ employ mathematical models to perform spatial searches, emphasizing both global exploration and local exploitation. Other notable algorithms in this category include Henry Gas Solubility Optimization (HGSO)^65^, Thermal Exchange Optimization (TEO)^66^, Atomic Orbital Search (AOS)^67^, Arithmetic Optimization Algorithm (AOA)^68^ and PID-based search algorithm (PSA)^69^. These algorithms benefit from their ability to model complex physical and mathematical phenomena, providing robust optimization capabilities.

**Human/Social-Based Algorithms**

Human and social-based algorithms are inspired by human behaviors, social interactions, sports and decision-making processes. These algorithms model various human activities such as political strategies, game playing, and social cooperation. The Political Optimizer (PO)^70^ simulates decision-making processes in political systems, while the Volleyball Premier League Algorithm (VPLA)^71^ models competitive interactions between volleyball teams. The Forensic-Based Investigation (FBI)^72^ algorithm mimics investigative techniques, and The Football Team Training Algorithm (FTTA)^21^ simulates the three stages of the training session: Collective Training, Group Training and Individual Extra Training. Other algorithms in this category include Transit Search (TS)^73^, Queuing Search Algorithm (QSA)^74^, and the Future Search Algorithm (FSA)^75^. These algorithms incorporate elements of human intuition and social dynamics to enhance optimization performance.

Meta-heuristic algorithms offer powerful tools for solving complex optimization problems across various domains^76–78^. Each category, whether inspired by natural evolution, swarm intelligence, physical laws, or human social behaviors, brings unique strengths and strategies to the optimization process. The continuous development and refinement of these algorithms hold significant promise for addressing increasingly complex and large-scale optimization challenges in the future^79–81^.

**Table 1:** Reviews of Meta-Heuristic Algorithms.

**Category**	**Algorithm**	**Inspiration**	**Year**
**Evolutionary Algorithms**	Genetic Algorithm (GA)	Natural selection	1996^41^
	Differential Evolution (DE)	Population evolution	1995^42^
	Evolution Strategy (ES)	Natural evolution	1973^43^
	Memetic Algorithm (MA)	Cultural evolution	1989^44^
	Evolutionary Programming (EP)	Genetic evolution	1999^45^
	Genetic Programming (GP)	Biological evolution	1994^46^
**Swarm Intelligence Algorithms**	Particle Swarm Optimization (PSO)	Foraging behavior of birds	1995^51^
	Ant Colony Optimization (ACO)	Pheromone trail-laying behavior of ants	2005^36^
	Artificial Bee Colony (ABC)	Foraging behavior of bees	2010^52^
	Firefly Algorithm (FA)	Flashing behavior of fireflies	2013^53^
	Grey Wolf Optimizer (GWO)	Leadership hierarchy of grey wolves	2014^20^
	Harris Hawks Optimization (HHO)	Cooperative hunting of Harris’ hawks	2019^54^
	Chimp Optimization Algorithm (ChOA)	Social hierarchy of chimpanzees	2020^55^
	Smell Agent Optimization (SAO)	Smell-based search behavior	2021^35^
	Reptile Search Algorithm (RSA)	Movement patterns of reptiles	2022^56^
	Beluga Whale Optimization (BWO)	Hunting strategies of beluga whales	2022^57^
	Aquila optimizer (AO)	Hunting behavior of eagles	2021^58^
	Artificial Protozoa Optimizer (APO)	Behavior of protozoa	2024^31^
	Crested Porcupine Optimizer (CPO)	Defense mechanism of porcupines	2024^59^
	Horned lizard optimization (HLOA)	Defense mechanism of horned lizards	2024^60^
	Electric eel foraging optimization (EEFO)	Foraging behavior of electric eels	2024^34^
**Physics/Mathematics-Based Algorithms**	Simulated Annealing (SA)	Annealing process in metallurgy	1993^61^
	Gravitational Search Algorithm (GSA)	Law of gravity	2009^62^
	Sine Cosine Algorithm (SCA)	Sine and cosine mathematical model	2016^63^
	Gradient-Based Optimizer (GBO)	Gradient search rules	2020^64^
	Henry Gas Solubility Optimization (HGSO)	Henry’s law of gas solubility	2019^65^
	Thermal Exchange Optimization (TEO)	Heat transfer processes	2017^66^
	Atomic Orbital Search (AOS)	Atomic orbitals	2021^67^
	Arithmetic Optimization Algorithm (AOA)	Arithmetic operations	2021^68^
	PID-based search algorithm (PSA)	PID control	2023^69^
**Human/Social-Based Algorithms**	Political Optimizer (PO)	Decision-making in political systems	2020^70^
	Volleyball Premier League Algorithm (VPLA)	Competitive interactions in volleyball	2018^71^
	Forensic-Based Investigation (FBI)	Investigative techniques	2020^72^
	Football Team Training Algorithm (FTTA)	Training processes of football players	2024^21^
	Transit Search (TS)	Exoplanet exploration	2022^73^
	Queuing Search Algorithm (QSA)	Human queuing actions	2018^74^
	Future Search Algorithm (FSA)	Human future planning behaviors	2019^75^

## Basketball team optimization algorithm

### Inspiration

The Basketball Team Optimization Algorithm (BTOA) draws its inspiration from the dynamic and strategic nature of basketball. The algorithm mimics four key behaviors observed in basketball teams: High-intensity Training, Fast Break Strategy, Dynamic Positioning Strategy, and Ball Re-entry. These behaviors capture the essence of a team’s adaptability, strategic thinking, and coordinated efforts to achieve optimal performance.

**High-intensity Training:** In basketball, elite players engage in rigorous training sessions to improve their skills and endurance. This process involves looking up to the best player as a model and striving to overcome various distractions and fatigue. By emulating this high-intensity training, the algorithm ensures that solutions are constantly refined and improved, mirroring the players’ relentless pursuit of excellence.

**Fast Break Strategy:** A fast break in basketball represents a rapid transition from defense to offense, aiming to score before the opposing team can set up its defense. This strategy highlights the importance of quick decision-making and precision. In BTOA, the fast break strategy accelerates the search process by swiftly moving towards promising solutions, akin to a player advancing towards the basket with speed and agility.

**Dynamic Positioning Strategy:**  Basketball players continuously adjust their positions on the court to cooperate with teammates and find optimal scoring opportunities. This dynamic positioning is crucial for effective teamwork and strategy execution. BTOA incorporates this strategy by enabling solutions to dynamically adjust their positions within the search space, ensuring a thorough exploration and exploitation of potential solutions.

**Ball Re-entry:**  When the ball goes out of bounds in basketball, it must be re-entered into play from the sideline, often to a random position. This action simulates the need to maintain the game flow despite disruptions. The boundary control strategy in BTOA, inspired by ball re-entry, ensures that solutions exceeding the search space boundaries are repositioned within feasible limits, maintaining the integrity of the optimization process.

### The construction of the mathematical model

This section introduces the detailed modeling process of the BTOA.

#### High-intensity training

**Fig. 1:**
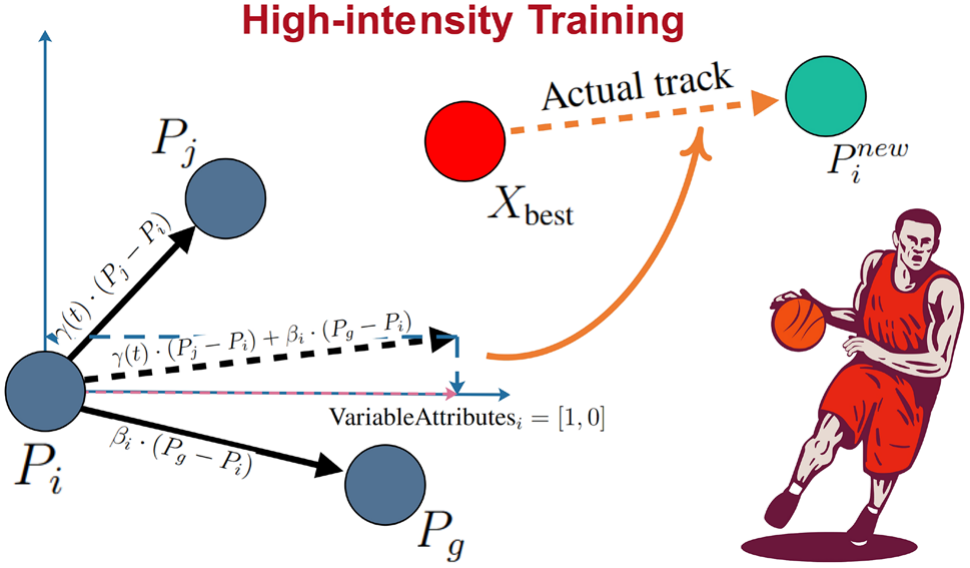
The process of high-intensity training.

In basketball training, elite players undergo high-intensity sessions, looking up to the best player as a model while facing various distractions. As training progresses, players become fatigued, diminishing their learning abilities and influence by others. Players differ in their ability to resist these distractions. The process of high-intensity training is modeled as follows:

1 $$\begin{aligned} P_{i}^{new} = X_{\text {best}} +\left( \gamma (t) \cdot (P_j - P_i) + \beta _i \cdot (P_g - P_i) \right) \odot \text {VariableAttributes}_i \end{aligned}$$

where $P_i$ is the current position of player *i*, $X_{\text {best}}$ is the best player’s position, and $P_j$ and $P_g$ are positions of two randomly selected players. The fatigue factor $\gamma (t)$ modulates peer influence over time, while the interference resilience coefficient $\beta _i$ measures player *i*’s ability to resist disruptions. The $\text {VariableAttributes}_i$ is a binary mask defining the dimensions in which player *i* can adjust. Figure 1 illustrates the process of high-intensity training in a 2D space.

*Maximum Changeable Attributes Coefficient* $\alpha _i$:. This coefficient is defined as:

2 $$\begin{aligned} \begin{aligned} \alpha _i =&\left\lceil \llceil \frac{D}{2} + \left( \frac{i - 1}{N - 1}\right) \times \frac{D}{2} \right\rceil \rrceil \\ \text {VariableAttributes}_i =&\text {CreateBinaryVector}(D, \alpha _i) \end{aligned} \end{aligned}$$

where *D* is the total number of dimensions, *i* is the player’s rank within the team, and *N* is the total number of players. The function $\text {CreateBinaryVector}(D, \alpha _i)$ generates a binary vector of length $D$ with exactly $\alpha _i$ entries set to 1, randomly chosen. This ensures each dimension has an equal chance of being selected for modification. The coefficient $\alpha _i$ determines the number of dimensions player *i* can adjust, based on their fitness rank. Players with lower fitness (higher *i* values) can adjust more attributes, ranging from 0.5*D* to *D*, which allows more low-rank players to explore the search space extensively, while more adept players focus on fine-tuning their strategies.

*Fatigue Factor* $$\gamma (t)$$.

3 $$\begin{aligned} \gamma (t) = 0.1 + 0.8 \times \sqrt{1 - \left( \frac{t}{T}\right) } \end{aligned}$$

where *t* represents the current iteration number, and *T* is the total number of iterations. This factor starts at 0.9 and decreases to 0.1 as *t* approaches *T*, representing how peer influence decreases as players become fatigued.

*Interference Resilience* $$\beta _i$$.

4 $$\begin{aligned} \beta _i = \frac{i}{\text {strongnum}} \end{aligned}$$

where *i* is the player’s rank within the team, and $\text {strongnum}$ is the number of players selected for intensive training. This coefficient increases with *i*, indicating that lower-ranked players (higher fitness) have poorer interference resilience. The parameter $\text {strongnum}$ is adjustable and defaults to $0.125 \times Teamsize$, where *Teamsize* is the number of players per iteration. The top $\text {strongnum}$ players are selected for high-intensity training.

**Differences among** $\beta _i$, $\alpha _i$, **and** $\gamma (t)$:

BTOA not only considers the balance between exploration and exploitation throughout the iterations but also emphasizes the balance within a single iteration among the population. In the case of a minimization problem, individuals with higher fitness (greater difference from the optimal solution) are more likely to explore better solutions. Therefore, BTOA ensures that within a single iteration, individuals with higher fitness have greater variability, enhancing their exploration of the search space and avoiding local optima.

Specifically:

- $\beta _i$ and $\alpha _i$ are used to adjust the scale of changes within the population during a single iteration, making sure that individuals with higher fitness have more room for adjustment.
- $\gamma (t)$ regulates the scale of changes across different iterations, gradually reducing the influence of peer learning as fatigue sets in.

In this way, BTOA dynamically balances the exploration and exploitation processes both within each iteration and throughout the entire optimization process, leading to more efficient convergence.

#### Fast break strategy

During a basketball game, a fast break represents a critical offensive strategy where a player rapidly advances the ball towards the opponent’s basket to score before the defense can properly set up. In this simulation, player *i* acts as the player executing the fast break, with $X_{\text {best}}$ symbolizing the basketball hoop(target). The player moves swiftly across the court, navigating through defensive players who might impede their path. The factor *f* serves as an adjustment coefficient, which reflects the player’s improving precision and confidence as the game progresses, changing from 2 to 1, indicating an enhancement in shooting accuracy.

The mathematical model for this process is given by:

5 $$\begin{aligned} \begin{aligned} \text {P}_{i}^{new}&= \text {P}_{i} + ( \text {rand} \odot f \cdot ( X_{\text {best}} - \text {P}_{i}) + \text {rand} \odot (1 - \frac{t}{\text {T}}) \cdot (\text {P}_j - \text {P}_g) )\odot \text {VariableAttributes}_i \end{aligned} \end{aligned}$$

where $P_{i}$ is the player’s current position, $X^*$ represents the target position (basket), $P_j$ and $P_g$ are the positions of opponents or defenders, $\text {rand}$ is a vector of random numbers between 0 and 1 introducing variability, and $VariableAttributes_i$ is a binary vector determining active dimensions. The adjustment factor *f* increases as the game progresses, improving the player’s shooting accuracy. The term $(1 - \frac{t}{T})$ simulates the player evading defensive interference during a fast break. Algorithmically, these settings shift the focus from exploration to exploitation as iterations increase.

**Fig. 2:**
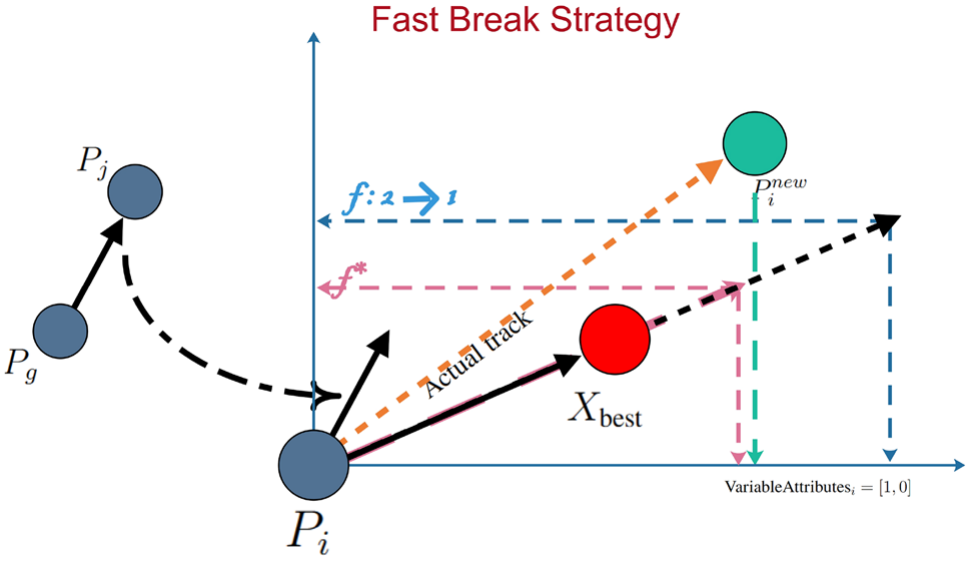
The process of fast break strategy.

Figure 2 illustrates the process of Fast Break Strategy in a 2D space.

*Adjustment Factor* *f*.

6 $$\begin{aligned} f = 2 - \left( \frac{t}{\text {T}}\right) ^2 \end{aligned}$$

where $t$ is the current iteration number, $T$ is the total number of iterations, and $f$ decreases from 2 to 1 as iterations progress. This simulates the player’s improvement in shooting accuracy and decision-making, reflecting how they get into the groove and become more accurate over time.

#### Dynamic positioning strategy

In basketball, players constantly adjust their positions to cooperate with teammates and find the optimal spot to receive passes and score. The inspiration for the strategy comes from this dynamic positioning process. In this strategy, two players coordinate their movements, and the ball is passed to the player who reaches a better position.

**Fig. 3:**
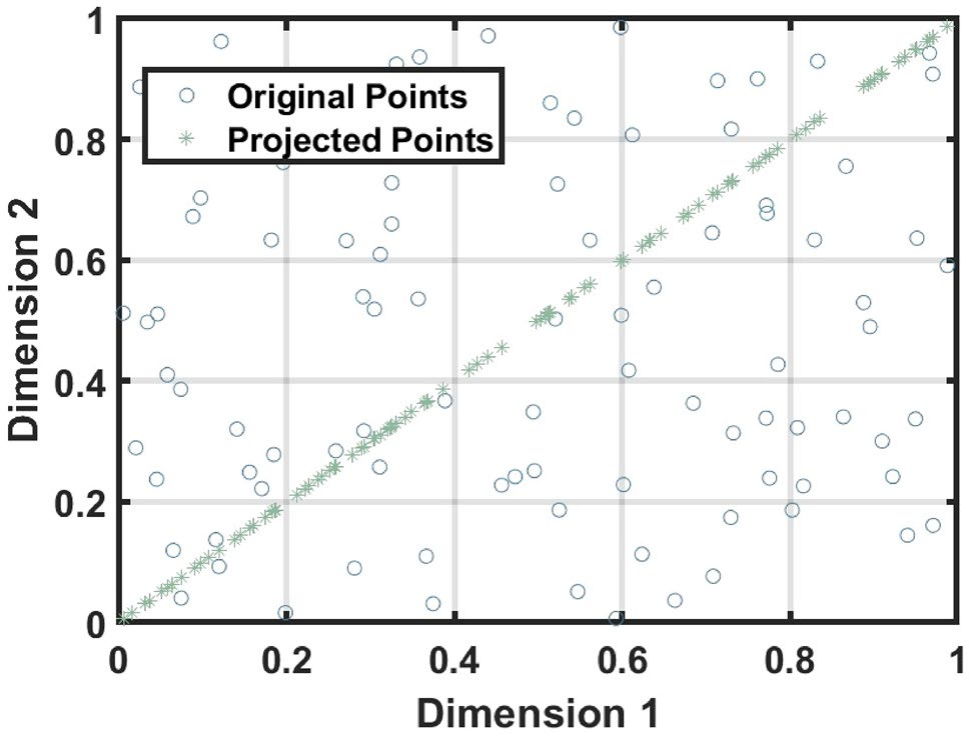
Projected points.

In the Dynamic Positioning Strategy, we use the diagonal as a guide for global search. We conducted a simple simulation where 100 points were randomly generated in a 2D space and projected onto the diagonal based on a random dimension. The projected points are uniformly distributed along the diagonal, demonstrating its global guiding effect for thoroughly exploring the solution space. Figure  3 illustrates the diagonal projection process.

The Dynamic Positioning Strategy consists of two approaches: the Random Position Strategy and the Diagonal Position Strategy.

**Random Position Strategy:**

In the Random Position Strategy, a player is randomly selected, and based on a randomly chosen dimension, the player is projected onto the diagonal. Starting from this diagonal position, a new candidate position is generated. This strategy simulates players moving randomly on the court to find openings.

*a. Randomly select a player and dimension*.

7 $$\begin{aligned} P_{\text {rand}}&= \text {RandomPlayer}(N) \end{aligned}$$

8 $$\begin{aligned} d_{\text {rand}}&= \text {RandomDimension}(D) \end{aligned}$$

where $N$ is the total number of players, $D$ is the total number of dimensions, $P_{\text {rand}}$ is the randomly selected player, and $d_{\text {rand}}$ is the randomly selected dimension.

*b. Calculate the player’s diagonal projection position*. 

9 $$\begin{aligned} L_{\text {rand}} = \text {lb} + \left( \frac{P_{\text {rand}}(d_{\text {rand}}) - \text {lb}(d_{\text {rand}})}{\text {ub}(d_{\text {rand}}) - \text {lb}(d_{\text {rand}})} \right) \cdot (\text {ub} - \text {lb}) \end{aligned}$$

where $\text {lb}$ and $\text {ub}$ are the lower and upper bounds of the search space, respectively, and $L_{\text {rand}}$ is the player’s diagonal projection position. Through this method, the player is projected onto the diagonal based on the randomly chosen dimension $d_{\text {rand}}$.

*c. Generate the candidate position* $C_1$.

10 $$\begin{aligned} Q_1&= L_{\text {rand}} + \text {rand} \odot (X_{\text {best}} - L_{\text {rand}}) \end{aligned}$$

11 $$\begin{aligned} C_1&= Q_1 + \text {randn} \odot (Q_1 - \delta \cdot P_i) \end{aligned}$$

where $X_{\text {best}}$ is the current best solution, $Q_1$ is the candidate position extending from the diagonal position $L_{\text {rand}}$ to the best solution, and $C_1$ is the final candidate position with random perturbations. Here, $\delta$ represents a random binary vector containing either 0 or 1, introducing variability to simulate disturbance in each dimension. The formula for $C_1$ combines the influence of the best solution $X_{\text {best}}$ with random perturbations, modeling the dynamic and unpredictable movement of a player on the court.

**Diagonal Position Strategy:**

In the Diagonal Position Strategy, the best solution is projected onto the diagonal, then a random position is generated, and a new candidate position is created based on symmetry around the diagonal position. The diagonal can span the entire search space, providing guidance that helps avoid local optima. This strategy simulates players moving along the diagonal on the court to find optimal attacking positions.

*a. Calculate the best player’s diagonal projection position*.

12 $$\begin{aligned} L_{\text {best}} = \text {lb} + \left( \frac{X_{\text {best}}(d_{\text {best}}) - \text {lb}(d_{\text {best}})}{\text {ub}(d_{\text {best}}) - \text {lb}(d_{\text {best}})} \right) \cdot (\text {ub} - \text {lb}) \end{aligned}$$

where $d_{\text {best}}$ is the randomly selected dimension of the best player, and $L_{\text {best}}$ is the best player’s diagonal projection position.

*b. Generate a random position*.

13 $$\begin{aligned} R = \text {lb} + \text {rand} \odot (\text {ub} - \text {lb}) \end{aligned}$$

where $R$ is the randomly generated position in the search space.

*c. Generate the candidate position* $C_2$.

14 $$\begin{aligned} C_2(d) = {\left\{ \begin{array}{ll} L_{\text {best}}(d) + \text {rand} \cdot (\text {ub}(d) - L_{\text {best}}(d)) & \text {if } R(d) < L_{\text {best}}(d) \\ \text {lb}(d) + \text {rand} \cdot (L_{\text {best}}(d) - \text {lb}(d)) & \text {otherwise} \end{array}\right. } \end{aligned}$$

where $C_2$ is the candidate position generated based on the symmetric point extended from the diagonal position. This formula essentially generates a point along the extension of the symmetric line without exceeding the boundaries. The significance of this process is to explore new potential solutions by leveraging symmetry and randomness, mimicking the strategic positioning adjustments made by players to optimize their placement on the court.

**Select the better solution**

Between the two candidate positions $R_1$ and $Q_2$ generated by the two positioning strategies, the one with the better fitness is selected as the new solution:

15 $$\begin{aligned} P_{i}^{new} = {\left\{ \begin{array}{ll} C_1 & \text {if } f(C_1) < f(C_2) \\ C_2 & \text {otherwise} \end{array}\right. } \end{aligned}$$

where $P_{i}^{new}$ is the new solution, and $f(C_1)$ and $f(C_2)$ are the fitness values of $C_1$ and $C_2$, respectively. By selecting the solution with the better fitness, the algorithm ensures that the ball is effectively passed to the player in the optimal position.

**Fig. 4:**
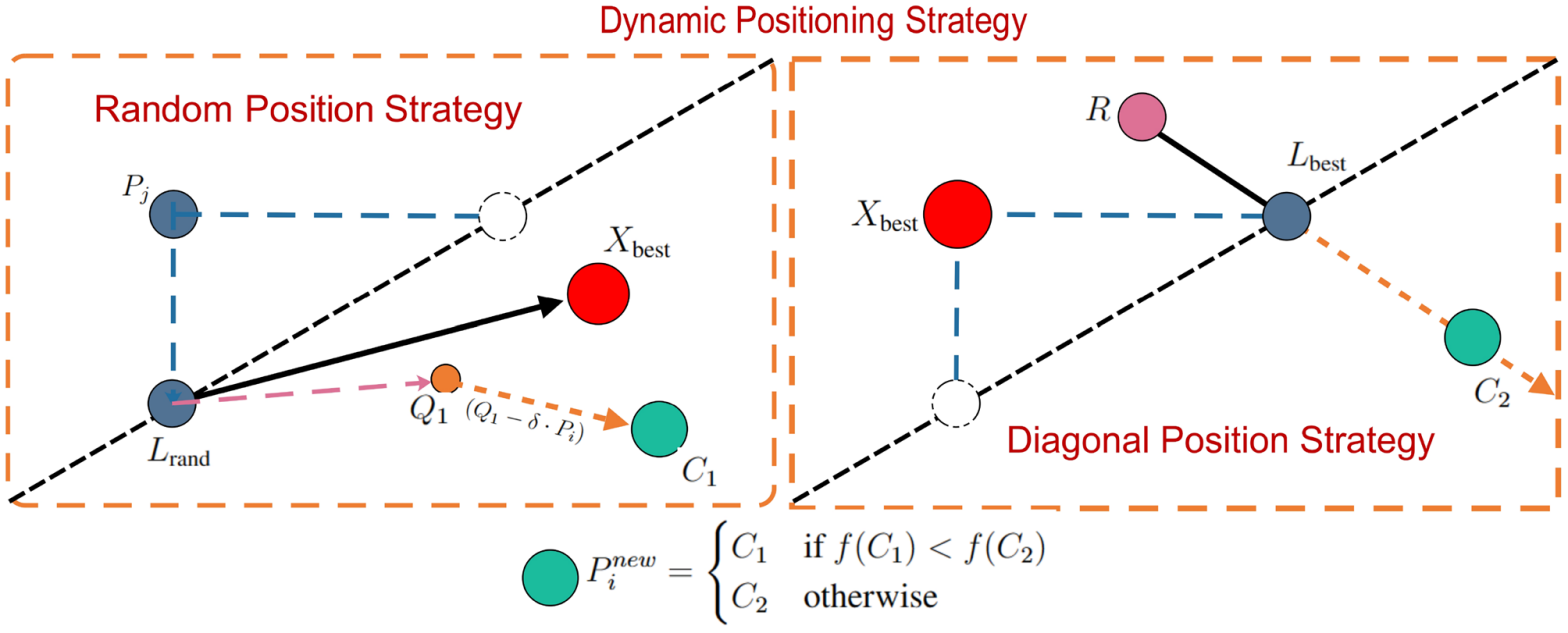
The process of dynamic positioning strategy.

Figure 4 illustrates the process of Dynamic Positioning Strategy in a 2D space.

#### Boundary control strategy: simulated ball re-entry

In a basketball game, when the ball goes out of bounds, players need to re-enter the ball from the sideline by passing it to a random position within the court. Inspired by this, the boundary control strategy in the Basketball Team Optimization Algorithm (BTOA) ensures that any solution exceeding the search space boundaries is randomly repositioned within the bounds, maintaining solution validity during the optimization process.

*Mathematical Formulation*. The mathematical formulation of this strategy is as follows:

16 $$\begin{aligned} X_d = {\left\{ \begin{array}{ll} q \cdot \text {ub}_d + (1 - q) \cdot \text {oldPos}_d & \text {if } X_d > \text {ub}_d \\ p \cdot \text {lb}_d + (1 - p) \cdot \text {oldPos}_d & \text {if } X_d < \text {lb}_d \end{array}\right. } \end{aligned}$$

where $X_d$ is the solution’s position in the $d$-th dimension, $\text {ub}_d$ and $\text {lb}_d$ are the upper and lower bounds of the search space in the $d$-th dimension, respectively. $\text {oldPos}_d$ is the solution’s previous position in the $d$-th dimension, $q$ and $p$ are random numbers between 0 and 1. This ensures that if a solution goes out of bounds, it is repositioned randomly but remains within the feasible search space, simulating the re-entry of a basketball into the court after going out of bounds.

#### Adaptivity factor for strategy adjustment

**Fig. 5:**
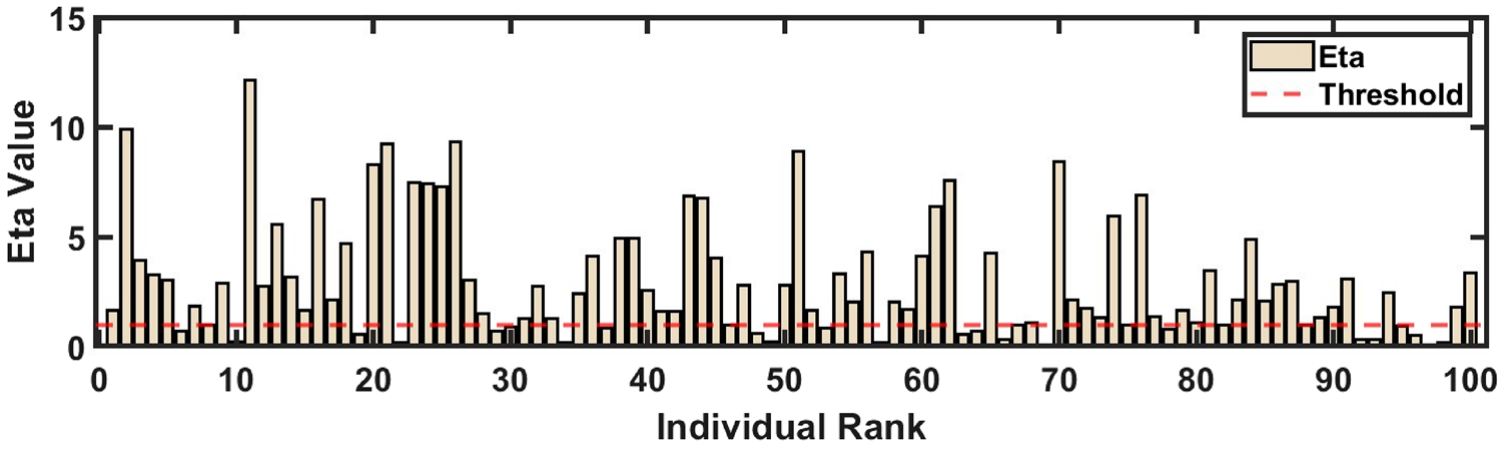
Distribution of the adaptivity factor $\eta$ across individual ranks.

The Adaptivity Factor ($\eta$) is used to switch between the fast break and dynamic positioning strategies. This factor is designed such that higher-ranked individuals have a greater probability of executing a fast break, while lower-ranked individuals are more likely to engage in dynamic positioning. If $\eta > 1$, the fast break strategy is chosen, while if $\eta < 1$, the dynamic positioning strategy is selected. The Adaptivity Factor ($\eta$) is defined as follows:

17 $$\begin{aligned} \eta = \kappa \cdot \exp \left( -\lambda \cdot \frac{i}{\text {TeamSize}}\right) \cdot \log \left( \frac{1}{\text {rand}}\right) \end{aligned}$$

where $\kappa$ and $\lambda$ are adjustable hyperparameters, $i$ is the individual’s rank within the team, $\text {TeamSize}$ is the total number of individuals, and $\text {rand}$ is a random number between 0 and 1. By default, $\kappa = 5$ and $\lambda = 1$. $\kappa$ influences the overall values of $\eta$, making higher values more likely and thus increasing the probability of a fast break. $\lambda$ controls the decay rate of $\eta$ with respect to the individual’s rank, increasing the likelihood of dynamic positioning for lower-ranked individuals. Figure 5 shows the distribution of the Adaptivity Factor $\eta$ across individual ranks. The red dashed line indicates $\eta = 1$.

The probability that $\eta > 1$ can be determined as follows:

18 $$\begin{aligned} P(\eta> 1) = P\left( 5 \cdot \exp \left( -\frac{i}{\text {TeamSize}}\right) \cdot \log \left( \frac{1}{\text {rand}}\right) > 1\right) \approx 0.711 \end{aligned}$$

Given the default parameters $\kappa = 5$ and $\lambda = 1$, the probability that $\eta > 1$ is approximately 71.1%, which was obtained by simulating the process 10,000 times.

The Adaptivity Factor effectively balances the exploration and exploitation processes. The fast break strategy emphasizes exploitation in the early stages and shifts to exploration in the later stages, while the dynamic positioning strategy focuses on global search throughout. By adjusting the proportion and distribution of these two strategies in each iteration, the Adaptivity Factor enhances the algorithm’s ability to navigate the search space and converge.

### Execution flow and procedure of BTOA

**Fig. 6:**
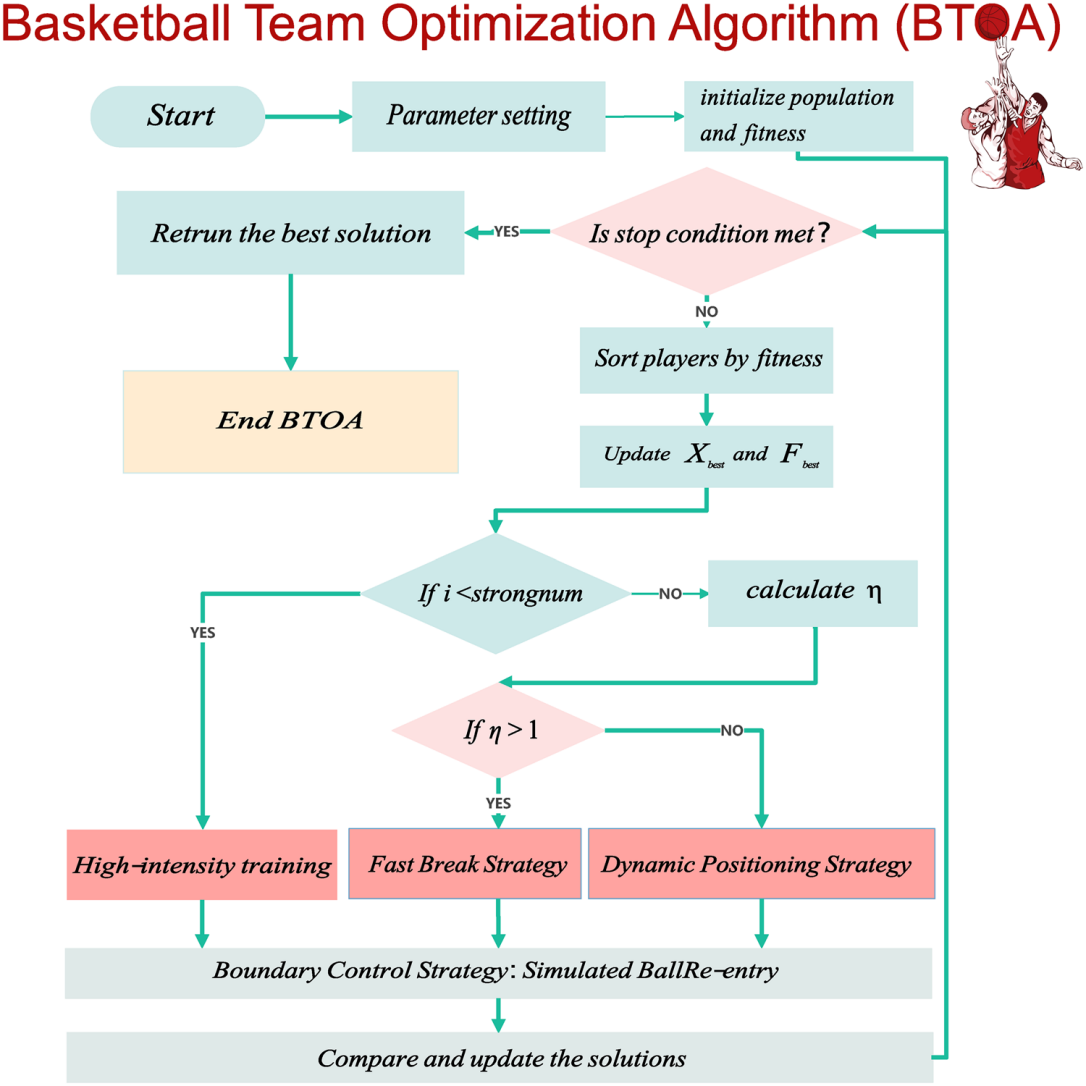
The execution flow chart of BTOA.

The execution flow of the Basketball Team Optimization Algorithm (BTOA) involves initializing the population of players, evaluating their fitness, and iteratively updating their positions based on different strategies inspired by basketball behaviors. The algorithm ensures that players with higher fitness have greater variability in their positions, balancing exploration and exploitation. The process continues until a a maximum number of iterations is met. The execution flow chart of BTOA is shown in Fig. 6.

The main steps of BTOA are as follows:

1. Initialize the population of players within the search space.
2. Evaluate the fitness of each player.
3. Sort the players based on their fitness and identify the best player.
4. Update player positions using High-intensity Training Strategy, Fast Break Strategy, or Dynamic Positioning Strategy.
5. Apply the Boundary Control Strategy to ensure all players remain within the search space.
6. Evaluate the new fitness of each player and update the best solution found.
7. Repeat steps 3–6 until the stopping criterion is met.

The detailed execution flow is presented in the Table 2.

**Table 2:**
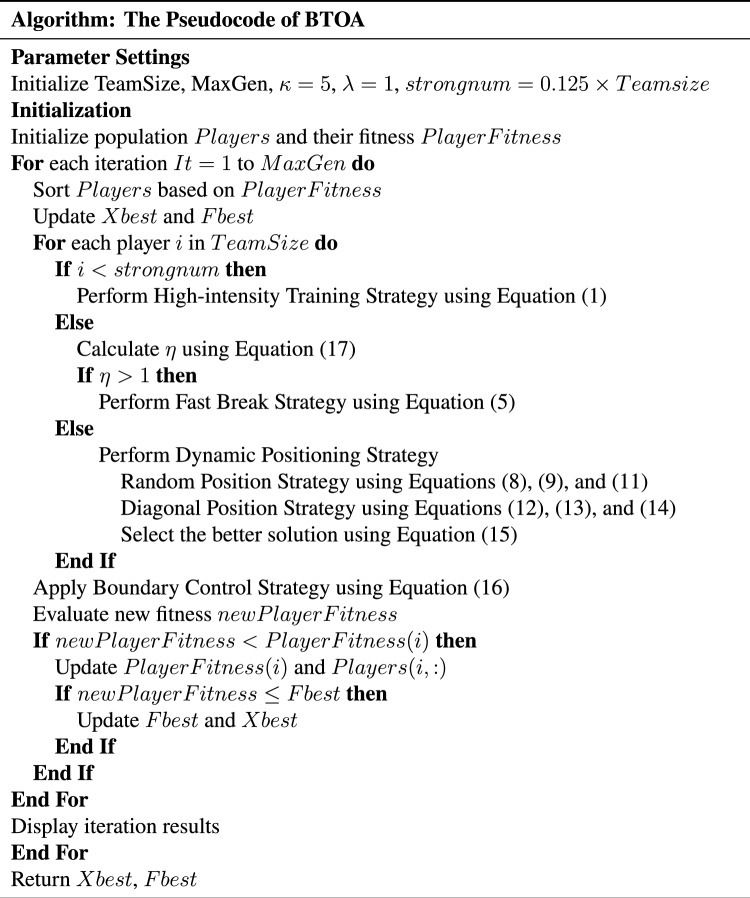
Pseudocode for BTOA.

### Computational complexity analysis

To thoroughly understand and evaluate the efficiency of BTOA, it is essential to analyze its computational complexity. The key parameters affecting the algorithm’s complexity include the population size (*TeamSize*), the dimensionality of the problem (*dim*), and the maximum number of iterations (*MaxGen*). BTOA’s computational complexity can be divided into two main parts: initialization and iterative process.

During the initialization phase, the population is initialized and their fitness values are computed. The complexity for this part is $O(\textit{TeamSize} \times \textit{dim})$. The iterative process, which includes sorting the population based on fitness, applying high-intensity training, fast break strategy, dynamic positioning strategy, and boundary control, is executed over *MaxGen* iterations. Each iteration involves operations with different complexities. For simplicity, we consider the worst-case scenario for each strategy:

- High-intensity Training Strategy: applied to the first 1/8 of the population.
- Fast Break Strategy: applied to approximately 70% of the remaining 7/8 of the population.
- Dynamic Positioning Strategy: applied to approximately 30% of the remaining 7/8 of the population.

The total computational complexity of BTOA is therefore given by:

19 $$\begin{aligned} \begin{aligned} O(\text {BTOA})&= O(\text {Initialization}) + O(\text {Iterative Process}) \\&= O(\textit{TeamSize} \times \textit{dim}) + \textit{MaxGen} \times \left[ O(\textit{TeamSize} \log \textit{TeamSize}) + O\left( \frac{1}{8} \textit{TeamSize} \times \textit{dim}\right) \right. \\&\quad + O\left( \frac{7}{8} \times 0.7 \times \textit{TeamSize} \times \textit{dim}\right) + O\left( \frac{7}{8} \times 0.3 \times \textit{TeamSize} \times \textit{dim}\right) \left. \right] \\&= O(\textit{MaxGen} \times \textit{TeamSize} \log \textit{TeamSize}) + O(\textit{MaxGen} \times \textit{TeamSize} \times \textit{dim}) \\&= O(\textit{MaxGen} \times \textit{TeamSize} \times (\log \textit{TeamSize} + \textit{dim})) \end{aligned} \end{aligned}$$

## Experiments on benchmark functions

### Numerical benchmarks and compared algorithms

To comprehensively evaluate the performance of our proposed algorithm, we employ two widely recognized benchmark suites: CEC2005^20,34^ and CEC2017^82^. The CEC2005 suite includes 7 unimodal functions (UFs) and 16 multimodal functions (MFs). Unimodal functions test the exploitation capabilities of algorithms with a single global optimum, while multimodal functions evaluate exploration abilities with multiple local optima. The CEC2017 suite, which extends these challenges by including hybrid composite, rotated, and shifted functions, tests an algorithm’s ability to handle complex and diverse landscapes. For CEC2017, we conducted experiments with 30, 50, and 100 dimensions. Combined with the fixed dimension of CEC2005, this results in a total of four experimental groups, thoroughly verifying the algorithm’s scalability and robustness.

We compare our algorithm against 10 other optimization algorithms, which include six classical algorithms: Beluga Whale Optimization (BWO)^57^, Sine Cosine Algorithm (SCA)^63^, Arithmetic Optimization Algorithm (AOA)^68^, Jaya Algorithm^83^, Differential Evolution (DE)^42^, and Aquila Optimizer (AO)^58^. Additionally, we consider four recent advanced algorithms: Triangulation Topology Aggregation Optimizer (TTAO)^84^, PID-based Search Algorithm (PSA)^69^, Crested Porcupine Optimizer (CPO)^59^, and Horned Lizard Optimization Algorithm (HLOA)^60^. These algorithms represent a broad spectrum of heuristic optimization techniques. Table 3 lists the parameters for each algorithm, which are set to their default values as specified in their original publications. Due to differences in precision between scientific notation and MATLAB’s computational precision, we conducted the comparisons using MATLAB’s precision and then converted the results to scientific notation.

**Table 3:** Parameter setting of the comparison algorithms.

Category	Algorithm	Parameters	Year
*All algorithms are set with a population size of 100 and 1000 iterations.*			
Classic Optimization Algorithms	BWO	Probability of a whale falling ($W_f$) = [0.1, 0.05]	2022
	SCA	Parameter (*a*) = 2	2016
	AOA	Control parameter $\sigma$ = 0.499 and sensitive parameter $\nu$ = 0.5	2021
	Jaya	*Parameters not specified*	2016
	DE	*f* = 0.7, *cr* = 0.1	1995
	AO	*u* = 0.0265; $r_0$ = 10; $\omega$ = 0.005; $\phi _0$ = $\frac{3\pi }{2}$	2021
Recent Advanced Algorithms	TTAO	*Parameters not specified*	2024
	PSA	$K_p$ = 1; $K_i$ = 0.5; $K_d$ = 1.2	2023
	CPO	$N_{\text {min}}$ = 15; *T* = 2; $\alpha$ = 0.2; $T_f$ = 0.8	2024
	HLOA	*Parameters not specified*	2024
Proposed Algorithm	BTOA	*kappa* = 5; $\lambda$ = 1; *strongnum* = 0.125 $\times$ Teamsize	-

To ensure a fair comparison, all algorithms are tested with a population size of 100 and run for 1000 iterations. Each experiment is repeated 30 times to mitigate the effects of randomness and to provide statistically significant results. The computational experiments are conducted on a system equipped with an Intel Core i7-13620H processor and an RTX4050 graphics card, running Matlab 2022a. This standardized computational environment ensures consistency and reliability in our results.

The following sections will conduct Quantitative Analysis, Convergence Analysis, Stability Analysis, exploration and exploitation Analysis, and Statistical Analysis of BTOA based on results obtained from the two benchmark suites.

### Experiment results on CEC2005

This subsection will conduct both quantitative and qualitative analyses on the 23 test functions of CEC2005 to evaluate the performance of BTOA on unimodal functions (UFs, F1-F7) and multimodal functions^34^ (MFs, F8-F23), and compare it with other algorithms.

#### Quantitative analysis

In this subsubsection, we conduct a quantitative analysis of BTOA on the 23 test functions of CEC2005, exploring its performance on unimodal functions (UFs, F1-F7) and multimodal functions (MFs, F8-F23), and comparing it with other algorithms. The solution quality of BTOA is assessed using two metrics^84^: average value (AVE) and standard deviation (STD). Tables 5 and 6 presents the experimental results of all compared algorithms on the 23 test functions. The best results for each test function are highlighted in bold.

The 7 unimodal functions (UFs, F1-F7), each with a single optimal solution, primarily evaluate the exploitation capability of the algorithms. The results indicate that BTOA achieves the best average value on 5 out of the 7 UFs. Notably, BTOA attains the global optimum on F1-F4 and F6. While other algorithms like BWO, HLOA, and PSA also perform well, they only achieve the optimal value on a few functions. Additionally, the STD for BTOA is 0 on these five test functions, demonstrating that BTOA consistently reaches the optimal value across 30 runs. This highlights BTOA’s strong exploitation capability for problems with a single optimum.

The 16 multimodal functions (MFs, F8-F23) can evaluate the exploration capability of the algorithms. The results show that BTOA achieves the best average value on 14 out of the 16 MFs. Although BTOA does not achieve the best value on F13 and F20, it comes very close to the optimal value. BTOA also excels in terms of STD, achieving the best average value on most functions. This demonstrates BTOA’s robust performance in handling functions with multiple local optima.

Overall, BTOA performs well on both unimodal and multimodal functions. As shown in the Table4, BTOA achieves the best results on 82.61% of the test functions compared to other algorithms on the CEC2005 benchmark suite.

**Table 4:** Number of times each algorithm achieved the lowest fitness in CEC2005.

BTOA	BWO	SCA	AOA	Jaya	DE	AO	TTAO	PSA	CPO	HLOA
**19**	7	0	4	1	9	3	8	5	0	7

**Table 5:** Performance comparison of various algorithms in CEC2005 (F1-F15).

Fun.	Index	BTOA	BWO	SCA	AOA	Jaya	DE	AO	TTAO	PSA	CPO	HLOA
F1	AVE	**0.00E+00**	**0.00E+00**	1.44E-04	1.57E-206	4.25E+00	6.79E-12	1.86E-298	7.74E-08	6.39E-17	1.95E+03	**0.00E+00**
	STD	**0.00E+00**	**0.00E+00**	5.25E-04	**0.00E+00**	1.28E+00	1.93E-12	**0.00E+00**	1.01E-07	1.90E-16	2.56E+03	**0.00E+00**
F2	AVE	**0.00E+00**	1.00E-265	6.97E-07	**0.00E+00**	2.36E+00	5.89E-08	1.71E-154	3.45E-04	5.27E-07	1.63E+01	5.68E-273
	STD	**0.00E+00**	**0.00E+00**	1.92E-06	**0.00E+00**	9.04E-01	7.84E-09	8.96E-154	1.42E-03	1.30E-06	1.07E+01	**0.00E+00**
F3	AVE	**0.00E+00**	**0.00E+00**	6.17E+02	4.01E-04	3.31E+04	2.27E+04	8.62E-237	2.01E+01	4.76E+00	6.46E+03	**0.00E+00**
	STD	**0.00E+00**	**0.00E+00**	8.80E+02	2.16E-03	4.86E+03	3.34E+03	**0.00E+00**	1.10E+01	3.74E+00	8.14E+03	**0.00E+00**
F4	AVE	**0.00E+00**	4.57E-257	8.33E+00	7.54E-03	2.29E+01	2.07E+00	9.62E-155	3.22E+00	4.98E-01	2.03E+01	5.36E-273
	STD	**0.00E+00**	**0.00E+00**	6.43E+00	1.55E-02	4.06E+00	2.27E-01	4.48E-154	1.02E+00	2.92E-01	1.05E+01	**0.00E+00**
F5	AVE	2.36E+01	**3.54E-17**	3.04E+01	2.77E+01	1.65E+03	3.63E+01	1.41E-04	6.45E+01	5.24E+01	5.73E+05	2.67E+01
	STD	1.78E-01	**1.56E-16**	9.09E+00	5.30E-01	3.50E+03	8.08E+00	2.68E-04	2.91E+01	4.11E+01	1.29E+06	7.14E+00
F6	AVE	**0.00E+00**	7.24E-12	7.23E-03	1.00E+00	6.73E-03	**0.00E+00**	3.43E-07	1.23E-33	**0.00E+00**	6.03E+01	2.31E-07
	STD	**0.00E+00**	5.57E-12	3.06E-03	1.90E-04	2.31E-03	**0.00E+00**	5.07E-07	3.70E-33	**0.00E+00**	6.79E+01	7.59E-07
F7	AVE	5.45E-05	1.93E-05	1.14E-02	**1.04E-05**	8.58E-02	2.20E-02	1.59E-05	3.68E-02	2.83E-02	8.13E-01	5.99E-05
	STD	3.73E-05	1.54E-05	9.55E-03	**9.84E-06**	2.20E-02	3.52E-03	1.32E-05	1.73E-02	1.40E-02	1.51E+00	5.21E-05
F8	AVE	**-1.26E+04**	**-1.26E+04**	-4.15E+03	-6.79E+03	-5.58E+03	-1.24E+04	-1.12E+04	-8.29E+03	-1.08E+04	-3.74E+03	-7.48E+03
	STD	**1.82E-12**	**1.82E-12**	2.04E+02	3.26E+02	6.87E+02	3.42E+02	2.55E+03	3.38E+02	5.02E+02	3.85E+02	6.82E+02
F9	AVE	**0.00E+00**	**0.00E+00**	8.69E-01	**0.00E+00**	2.41E+02	6.05E+01	**0.00E+00**	1.70E+01	1.87E+01	2.30E+02	**0.00E+00**
	STD	**0.00E+00**	**0.00E+00**	1.84E+00	**0.00E+00**	1.58E+01	4.29E+00	**0.00E+00**	4.83E+00	9.15E+00	8.39E+01	**0.00E+00**
F10	AVE	**8.88E-16**	**8.88E-16**	8.96E+00	**8.88E-16**	5.63E+00	6.89E-07	**8.88E-16**	4.21E+00	4.90E-09	8.98E+00	**8.88E-16**
	STD	**0.00E+00**	**0.00E+00**	9.29E+00	**0.00E+00**	5.45E+00	1.26E-07	**0.00E+00**	1.12E+00	7.66E-09	4.70E+00	**0.00E+00**
F11	AVE	**0.00E+00**	**0.00E+00**	4.63E-02	5.98E-02	1.04E+00	1.08E-10	**0.00E+00**	8.37E-03	2.22E-02	1.35E+01	**0.00E+00**
	STD	**0.00E+00**	**0.00E+00**	9.67E-02	6.07E-02	8.27E-03	8.86E-11	**0.00E+00**	9.90E-03	2.73E-02	1.48E+01	**0.00E+00**
F12	AVE	**1.57E-32**	1.76E-26	5.04E-01	2.01E-01	9.08E+00	9.54E-13	1.12E-07	5.65E-01	1.38E-02	1.92E+04	6.91E-03
	STD	**5.47E-48**	4.18E-26	1.90E-01	3.80E-02	2.29E+00	4.19E-13	2.32E-07	6.44E-01	5.82E-02	7.48E+04	2.59E-02
F13	AVE	**1.35E-32**	6.11E-26	2.21E+00	2.76E+00	8.11E+00	4.25E-12	7.03E-07	1.36E-01	3.66E-04	1.08E+06	1.86E-03
	STD	**5.47E-48**	6.80E-26	2.86E-01	1.34E-01	5.93E+00	1.35E-12	1.04E-06	1.92E-01	1.97E-03	4.79E+06	4.09E-03
F14	AVE	**9.98E-01**	9.98E-01	9.98E-01	7.00E+00	9.98E-01	**9.98E-01**	9.98E-01	**9.98E-01**	**9.98E-01**	2.53E+00	3.40E+00
	STD	**0.00E+00**	8.06E-15	2.68E-04	4.32E+00	7.63E-05	**0.00E+00**	5.53E-10	**0.00E+00**	1.62E-16	1.39E+00	2.32E+00
F15	AVE	**3.07E-04**	3.13E-04	8.49E-04	3.71E-03	5.12E-04	5.95E-04	4.11E-04	3.24E-04	1.80E-03	5.45E-03	4.58E-03
	STD	**1.54E-19**	1.59E-05	4.03E-04	6.21E-03	3.36E-04	8.95E-05	7.31E-05	7.87E-05	4.97E-03	5.18E-03	7.90E-03

**Table 6:** Performance comparison of various algorithms in CEC2005 (F16-F23).

Fun.	Index	BTOA	BWO	SCA	AOA	Jaya	DE	AO	TTAO	PSA	CPO	HLOA
F16	AVE	**-1.03E+00**	-1.03E+00	-1.03E+00	-1.03E+00	-1.03E+00	**-1.03E+00**	-1.03E+00	**-1.03E+00**	**-1.03E+00**	-1.02E+00	**-1.03E+00**
	STD	6.66E-16	1.91E-05	8.13E-06	4.29E-08	8.30E-06	6.66E-16	4.34E-05	6.66E-16	5.94E-16	1.80E-02	**5.21E-16**
F17	AVE	**3.98E-01**	3.99E-01	3.98E-01	4.02E-01	3.98E-01	**3.98E-01**	3.98E-01	**3.98E-01**	**3.98E-01**	4.33E-01	**3.98E-01**
	STD	**0.00E+00**	7.54E-04	3.52E-04	3.73E-03	1.46E-04	**0.00E+00**	3.10E-05	**0.00E+00**	**0.00E+00**	3.14E-02	**0.00E+00**
F18	AVE	**3.00E+00**	3.18E+00	3.00E+00	4.80E+00	3.00E+00	**3.00E+00**	3.00E+00	**3.00E+00**	**3.00E+00**	3.51E+00	3.00E+00
	STD	1.32E-15	2.27E-01	2.15E-06	6.73E+00	1.42E-04	1.78E-15	2.43E-03	1.25E-15	**9.63E-16**	6.21E-01	9.39E-15
F19	AVE	**-3.86E+00**	-3.86E+00	-3.86E+00	-3.85E+00	**-3.86E+00**	**-3.86E+00**	-3.86E+00	**-3.86E+00**	**-3.86E+00**	-3.84E+00	-3.86E+00
	STD	2.66E-15	1.08E-03	2.80E-03	1.59E-03	2.66E-15	2.66E-15	1.15E-03	2.66E-15	**2.50E-15**	1.65E-02	1.41E-03
F20	AVE	-3.28E+00	-3.31E+00	-3.07E+00	-3.16E+00	-3.25E+00	**-3.32E+00**	-3.23E+00	**-3.32E+00**	-3.27E+00	-2.99E+00	-3.25E+00
	STD	5.59E-02	4.05E-03	1.19E-01	5.57E-02	5.77E-02	**1.33E-15**	6.29E-02	**1.33E-15**	5.89E-02	1.36E-01	5.91E-02
F21	AVE	**-1.02E+01**	-1.02E+01	-4.21E+00	-4.14E+00	-7.61E+00	-1.02E+01	-1.02E+01	**-1.02E+01**	-7.81E+00	-3.44E+00	-9.40E+00
	STD	6.95E-15	1.61E-06	1.77E+00	7.34E-01	2.27E+00	9.44E-11	1.24E-03	**6.79E-15**	3.35E+00	1.45E+00	2.26E+00
F22	AVE	**-1.04E+01**	-1.04E+01	-5.34E+00	-4.99E+00	-9.17E+00	**-1.04E+01**	-1.04E+01	**-1.04E+01**	-8.03E+00	-3.40E+00	-9.07E+00
	STD	1.26E-15	4.00E-06	1.59E+00	1.28E+00	1.39E+00	**1.03E-15**	5.21E-04	**1.03E-15**	3.16E+00	1.24E+00	2.99E+00
F23	AVE	**-1.05E+01**	-1.05E+01	-5.86E+00	-5.08E+00	-9.04E+00	**-1.05E+01**	-1.05E+01	**-1.05E+01**	-7.70E+00	-2.87E+00	-8.98E+00
	STD	1.83E-15	2.36E-06	1.66E+00	1.75E+00	1.93E+00	**1.78E-15**	1.17E-03	1.83E-15	3.52E+00	9.06E-01	3.12E+00

#### Convergence analysis and stability analysis

**Fig. 7:**
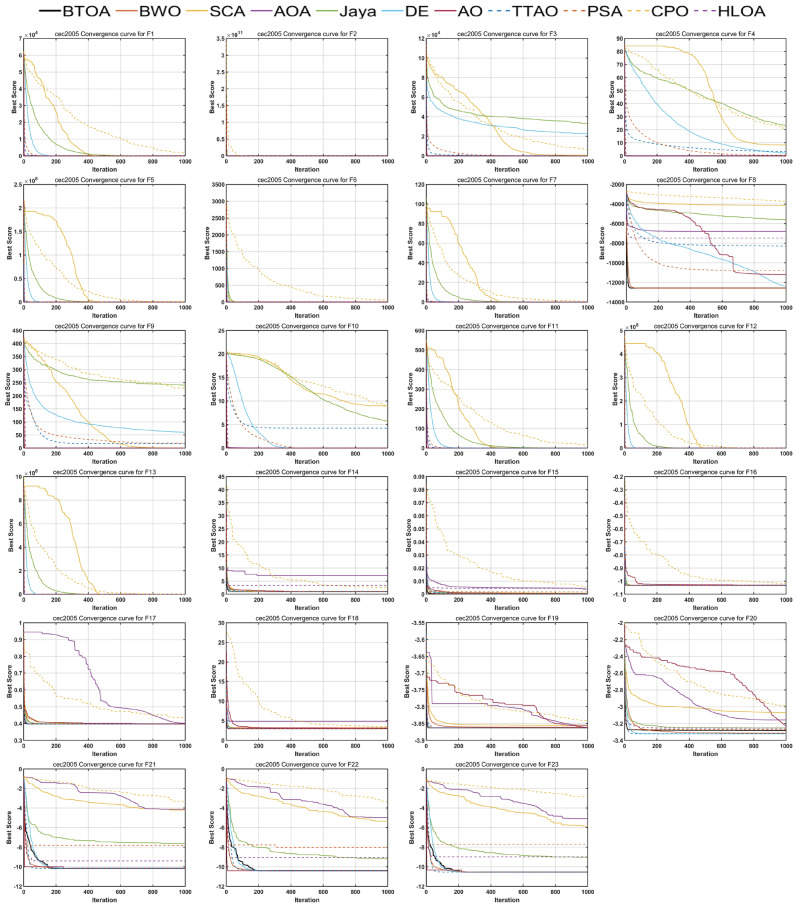
Convergence curves of BTOA in CEC2005.

The convergence capability of an algorithm is crucial. For unimodal functions with a single optimal solution, the algorithm should converge rapidly. For multimodal functions with multiple local optima, the algorithm should quickly converge while avoiding getting trapped in local optima. A competent heuristic optimization algorithm must adapt to these challenges. Figure  7 presents the convergence curves of BTOA compared to other algorithms. It can be observed that on the 7 UFs, BTOA exhibits a faster convergence rate than other algorithms, often finding the optimal solution in the early iterations. For the MFs, BTOA demonstrates rapid convergence on most functions, achieving superior solutions even in the presence of multiple peaks.

**Fig. 8:**
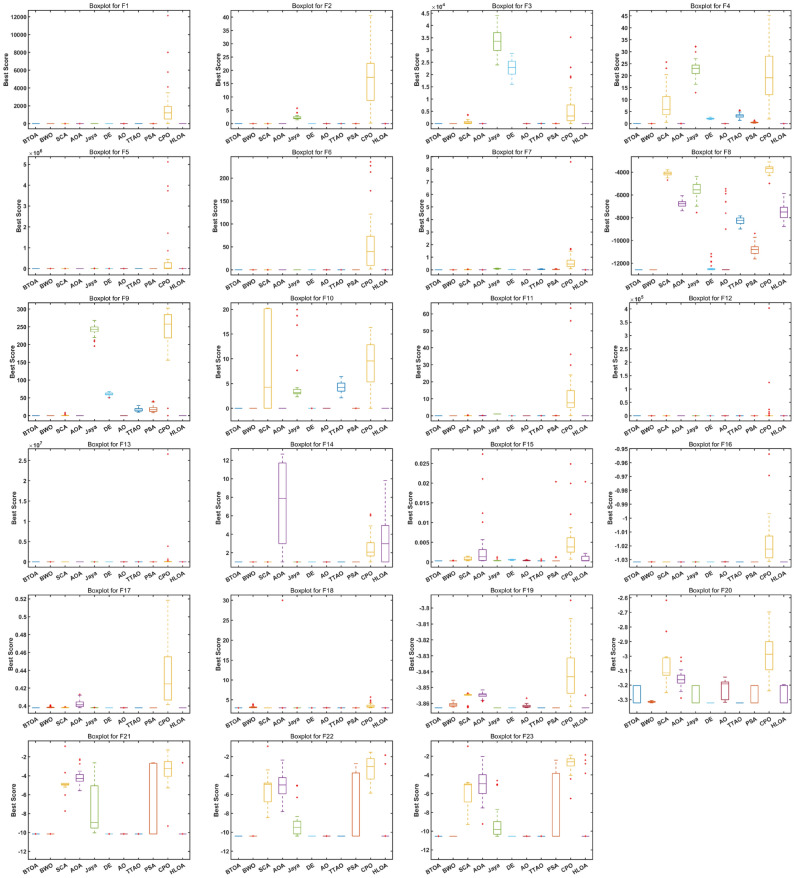
Box plot of BTOA in CEC2005.

To qualitatively analyze the stability of BTOA, we used box plots for visualization. Figure 8 shows the box plots of BTOA and other algorithms on the 23 test functions. It can be seen that the box plots of BTOA are the shortest in most cases, indicating that BTOA’s performance is consistently stable rather than coincidental.

#### Statistical analysis

We employed non-parametric test methods to evaluate the significant differences among the algorithms. The Wilcoxon signed-rank test^85^ was used to compare any two algorithms, while the Friedman^86^ test was applied for comparisons and rankings among multiple algorithms. The significance level $\alpha$ was set to 0.05.

Based on the results of 30 runs for each test function, we performed the Wilcoxon signed-rank test. Table  8 presents the results of the comparisons between BTOA and each algorithm. The P-values indicate the significance of the differences between the algorithms: ’+’ denotes that BTOA is significantly better than the compared algorithm, ’-’ indicates that BTOA is significantly worse, and ’=’ shows no significant difference between the algorithms. According to the statistical analysis, out of 230 pairwise comparisons, the BTOA algorithm outperforms other algorithms in 173 cases (75.22%). In 50 cases (21.74%), BTOA and the compared algorithms show similar performance. Only in 7 cases (3.04%) do other algorithms perform better than BTOA. This demonstrates the significant advantage of BTOA over the other algorithms.

Based on the mean results presented in Table  7, we conducted the Friedman test. The average rankings and overall rankings of the 11 algorithms across the 23 benchmark functions are summarized in Table 1. The results indicate significant differences among the algorithms, with BTOA achieving the highest average and overall rankings, followed by BWO, DE, AO, HLOA, TTAO, PSA, AOA, SCA, Jaya, and CPO.

**Table 7:** Friedman test on CEC2005.

	**BTOA**	**BWO**	**SCA**	**AOA**	**Jaya**	**DE**	**AO**	**TTAO**	**PSA**	**CPO**	**HLOA**	**p_value**
Mean of rank	**2.26**	3.96	8.22	7.20	8.39	4.48	4.89	5.26	5.72	10.70	4.93	**8.02E-22**
Overall rank	**1**	2	9	8	10	3	4	6	7	11	5	**8.02E-22**

**Table 8:** Wilcoxon signed-rank test on CEC2005.

BTOA vs	BWO		SCA		AOA		Jaya		DE		AO		TTAO		PSA		CPO		HLOA	
	**p**	**win**	**p**	**win**	**p**	**win**	**p**	**win**	**p**	**win**	**p**	**win**	**p**	**win**	**p**	**win**	**p**	**win**	**p**	**win**
**F1**	1.00E+00	=	1.73E-06	+	2.50E-01	=	1.73E-06	+	1.73E-06	+	1.73E-06	+	1.73E-06	+	1.73E-06	+	1.73E-06	+	1.00E+00	=
**F2**	1.73E-06	+	1.73E-06	+	1.00E+00	=	1.73E-06	+	1.73E-06	+	1.73E-06	+	1.73E-06	+	1.73E-06	+	1.73E-06	+	1.73E-06	+
**F3**	1.00E+00	=	1.73E-06	+	7.81E-03	+	1.73E-06	+	1.73E-06	+	1.73E-06	+	1.73E-06	+	1.73E-06	+	1.73E-06	+	1.00E+00	=
**F4**	1.73E-06	+	1.73E-06	+	1.23E-05	+	1.73E-06	+	1.73E-06	+	1.73E-06	+	1.73E-06	+	1.73E-06	+	1.73E-06	+	5.96E-05	+
**F5**	1.73E-06	-	1.73E-06	+	1.73E-06	+	1.73E-06	+	1.73E-06	+	1.73E-06	-	3.52E-06	+	1.11E-02	+	1.73E-06	+	3.59E-04	+
**F6**	1.73E-06	+	1.73E-06	+	1.73E-06	+	1.73E-06	+	1.00E+00	=	1.73E-06	+	2.50E-01	=	1.00E+00	=	1.73E-06	+	1.73E-06	+
**F7**	8.31E-04	-	1.73E-06	+	1.97E-05	-	1.73E-06	+	1.73E-06	+	1.06E-04	-	1.73E-06	+	1.73E-06	+	1.73E-06	+	8.94E-01	=
**F8**	1.00E+00	=	1.73E-06	+	1.73E-06	+	1.73E-06	+	1.73E-06	+	1.73E-06	+	1.73E-06	+	1.73E-06	+	1.73E-06	+	1.73E-06	+
**F9**	1.00E+00	=	1.73E-06	+	1.00E+00	=	1.73E-06	+	1.73E-06	+	1.00E+00	=	1.73E-06	+	1.73E-06	+	1.73E-06	+	1.00E+00	=
**F10**	1.00E+00	=	1.73E-06	+	1.00E+00	=	1.73E-06	+	1.73E-06	+	1.00E+00	=	1.73E-06	+	1.73E-06	+	1.73E-06	+	1.00E+00	=
**F11**	1.00E+00	=	1.73E-06	+	3.79E-06	+	1.73E-06	+	1.73E-06	+	1.00E+00	=	1.73E-06	+	1.73E-06	+	1.73E-06	+	1.00E+00	=
**F12**	1.73E-06	+	1.73E-06	+	1.73E-06	+	1.73E-06	+	1.73E-06	+	1.73E-06	+	1.73E-06	+	1.73E-06	+	1.73E-06	+	1.73E-06	+
**F13**	1.73E-06	+	1.73E-06	+	1.73E-06	+	1.73E-06	+	1.73E-06	+	1.73E-06	+	1.73E-06	+	1.73E-06	+	1.73E-06	+	1.73E-06	+
**F14**	4.18E-07	+	1.73E-06	+	1.73E-06	+	1.73E-06	+	1.00E+00	=	1.73E-06	+	1.00E+00	=	5.00E-01	=	1.73E-06	+	1.64E-05	+
**F15**	1.73E-06	+	1.73E-06	+	1.73E-06	+	1.73E-06	+	1.73E-06	+	1.73E-06	+	1.73E-06	+	2.09E-06	+	1.73E-06	+	1.45E-06	+
**F16**	1.73E-06	+	1.73E-06	+	1.73E-06	+	1.73E-06	+	1.00E+00	=	1.73E-06	+	1.00E+00	=	1.00E+00	=	1.73E-06	+	1.00E+00	=
**F17**	1.73E-06	+	1.73E-06	+	1.73E-06	+	1.23E-05	+	1.00E+00	=	1.73E-06	+	1.00E+00	=	1.00E+00	=	1.73E-06	+	1.00E+00	=
**F18**	1.73E-06	+	1.73E-06	+	1.73E-06	+	1.73E-06	+	1.80E-01	=	1.73E-06	+	1.00E+00	=	1.25E-01	=	1.73E-06	+	2.59E-06	+
**F19**	1.73E-06	+	1.73E-06	+	1.73E-06	+	1.00E+00	=	1.00E+00	=	1.73E-06	+	1.00E+00	=	1.00E+00	=	1.73E-06	+	1.00E+00	=
**F20**	6.44E-01	=	2.13E-06	+	8.47E-06	+	3.78E-02	+	4.88E-04	-	2.77E-03	+	4.88E-04	-	5.81E-01	=	1.92E-06	+	2.23E-02	+
**F21**	1.73E-06	+	1.73E-06	+	1.73E-06	+	1.73E-06	+	5.00E-01	=	1.73E-06	+	1.00E+00	=	1.95E-03	+	1.73E-06	+	6.25E-02	=
**F22**	1.73E-06	+	1.73E-06	+	1.73E-06	+	1.23E-05	+	1.00E+00	=	1.73E-06	+	1.00E+00	=	9.77E-04	+	1.73E-06	+	3.91E-03	+
**F23**	1.73E-06	+	1.73E-06	+	1.73E-06	+	8.30E-06	+	1.00E+00	=	1.73E-06	+	1.00E+00	=	4.88E-04	+	1.73E-06	+	1.95E-03	+

### Experiment results on CEC2017

To assess the capability of BTOA in solving complex optimization tasks, we employed a diverse set of 30 benchmark functions from the CEC2017 test suite. This suite includes two unimodal functions (C17F1 and C17F3), seven multimodal functions (C17F4-C17F10), 10 hybrid functions (C17F11-C17F20), and 10 composition functions (C17F21-C17F30)^87^. We tested these functions with dimensionalities of 30, 50, and 100, conducting both qualitative and quantitative analyses.

#### Quantitative analysis

The results, which include the mean and standard deviation of the best solutions obtained by BTOA and the other algorithms, are shown in Tables 11, 12, 13, 14, 15 and 16. The best outcomes for each test problem are highlighted in bold. Table 9 shows the number of functions in which each algorithm achieves the best solution in three different dimensions. The results indicate that BTOA achieves the best average value on 20 out of 30 test functions (66.67%) when the dimensionality is 30. When the dimensionality is increased to 50, BTOA achieves the best average value on 19 test functions (63.3%). For a dimensionality of 100, BTOA once again achieves the best average value on 20 test functions (66.67%). These findings demonstrate that BTOA’s performance remains consistent and robust across different dimensionalities. Whether in low-dimensional or high-dimensional settings, BTOA excels on the majority of functions in the CEC2017 benchmark suite.

**Table 9:** Number of times each algorithm achieved the lowest fitness in CEC2017.

	BTOA	BWO	SCA	AOA	Jaya	DE	AO	TTAO	PSA	CPO	HLOA
Dim=30	**20**	0	0	0	0	1	0	3	4	0	2
Dim=50	**19**	0	0	0	0	1	0	4	3	0	3
Dim=100	**20**	0	0	0	0	1	0	3	4	0	2

#### Convergence analysis and stability analysis

**Fig. 9:**
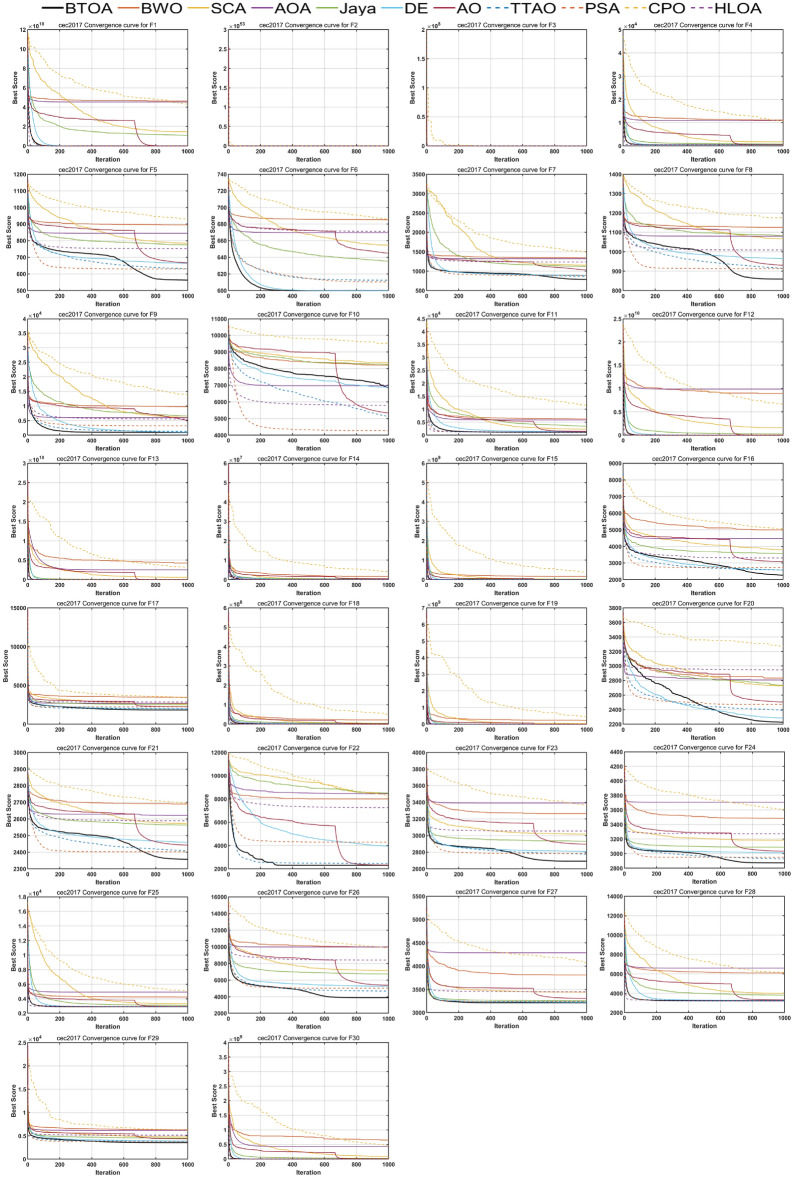
Convergence curves of BTOA in CEC2017 (Dim=30).

**Fig. 10:**
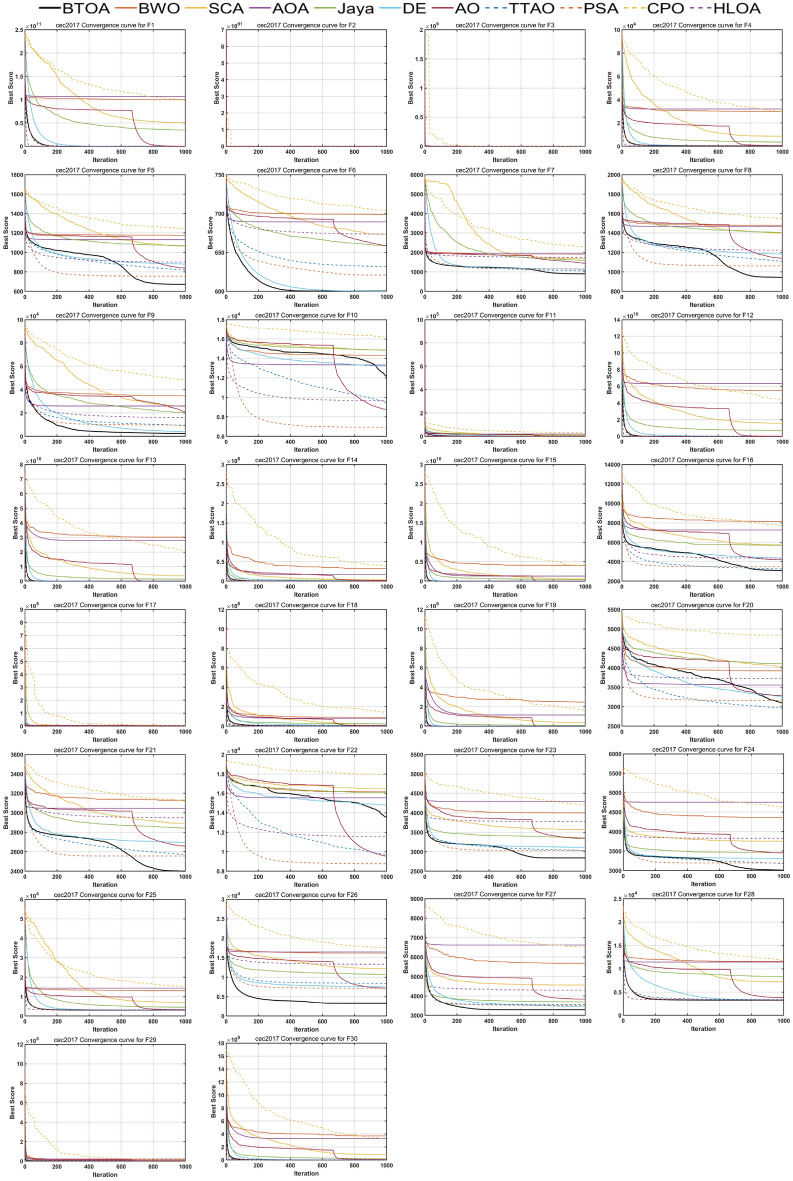
Convergence curves of BTOA in CEC2017 (Dim=50).

**Fig. 11:**
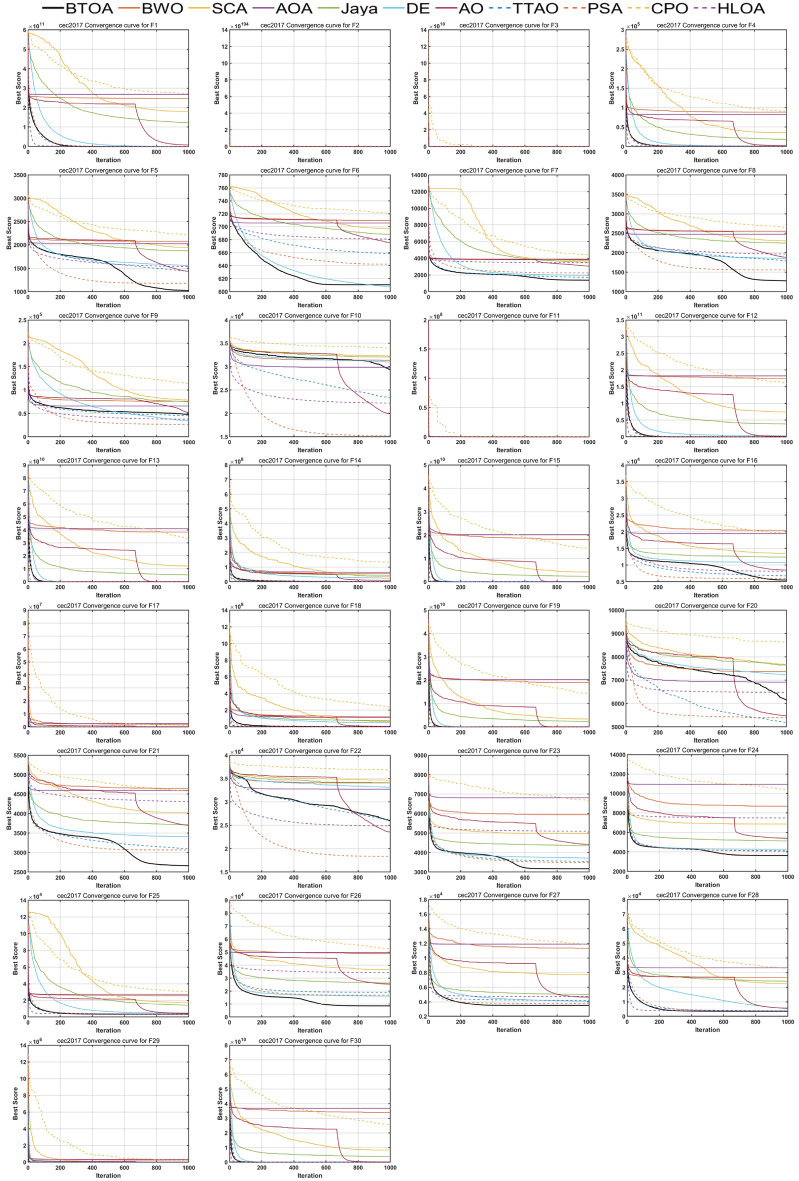
Convergence curves of BTOA in CEC2017 (Dim=100).

Figures 9, 10 and 11 illustrate the iterative curves of BTOA on CEC2017 benchmark functions with dimensionalities of 30, 50, and 100, respectively. As the dimensionality increases, the performance of BTOA remains relatively stable, with the shape of the iterative curves showing no significant changes for most test functions. It is evident that BTOA exhibits fast convergence on the majority of test functions and is capable of achieving superior solutions.

Notably, for functions F5, F7, F8, F21, F23, F24, and F26, although BTOA shows slower convergence in the early stages, there is a distinct inflection point in the mid-stages where the iterative curve rapidly declines, ultimately resulting in solutions better than those of the compared algorithms. This can be attributed to BTOA’s strong exploitation capability and its ability to escape local optima. However, as stated by the No Free Lunch (NFL) theorem^33^, no single metaheuristic optimization algorithm can perform optimally for all problems. While BTOA’s performance is commendable on most test functions, it performs relatively less effectively on F10 and F22, where it does not achieve better solutions after 1000 iterations. Nonetheless, the convergence trends of BTOA suggest that it still has potential for further improvement in the later stages, indicating its capacity for continued exploration of better solutions even after an initial slow start.

#### Exploration and exploitation analysis

This subsection will qualitatively analyze BTOA’s ability to balance exploration and exploitation^30^. Exploration involves searching broadly across the solution space to avoid local optima, while exploitation focuses on intensively searching promising areas to find optimal or near-optimal solutions. Striking this balance is critical for algorithm performance. Excessive exploration can lead to inefficiency and slow convergence, as the algorithm may spend too much time in less promising regions^87^. On the other hand, excessive exploitation may cause premature convergence to local optima, preventing the discovery of better solutions. To evaluate the performance of the proposed BTOA algorithm, we use a dimension-wise diversity measurement^84^. The rates of exploration and exploitation are calculated as follows:

20 $$\begin{aligned} Div^t= & \frac{1}{L} \sum _{k=1}^{L} Div_k \end{aligned}$$

21 $$\begin{aligned} Div_k= & \frac{1}{M} \sum _{m=1}^{M} \text {median}(y^k) - y_m^k \end{aligned}$$

where *L* denotes the variable dimension, $y_m^k$ is the *k*-th dimension of the *m*-th individual, and $\text {median}(y^k)$ is the median of the *k*-th variable in the population. The mathematical expressions to measure the percentage of exploration versus exploitation are written as:

22 $$\begin{aligned} Exploration\%= & \frac{Div_t}{Div_{\max }} \times 100 \end{aligned}$$

23 $$\begin{aligned} Exploitation\%= & \frac{|Div^t - Div_{\max }|}{Div_{\max }} \times 100 \end{aligned}$$

where $t = 1, 2, \ldots , P$, *P* represents the maximum number of iterations, $Div_t$ represents the population diversity at the *t*-th iteration, and $Div_{\max }$ is the maximum population diversity in the whole *P* iterations. In this study, we set the population size $M = 50$, the maximum number of iterations $P = 500$, and the dimension $L = 50$.

**Fig. 12:**
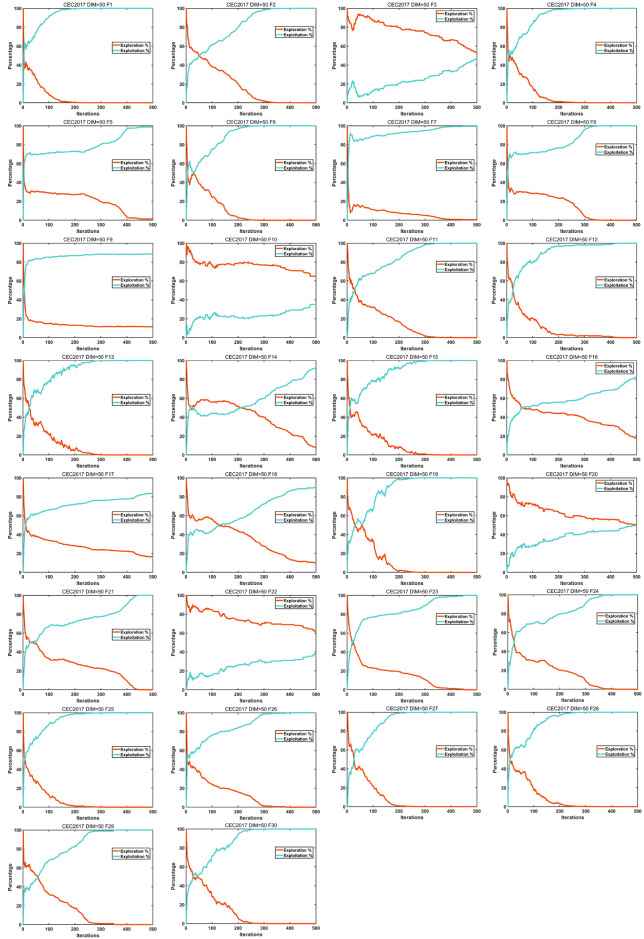
Exploration and exploitation ratios.

Figure 12 shows the variation of exploration and exploitation ratios with iterations for some test functions in the 50-dimensional CEC 2017 benchmark. As observed in Fig. 1, although the exploration and exploitation ratios vary for each test function, they exhibit a common trend: an emphasis on exploration in the early stages and a shift towards exploitation in the later stages. The varying ratios for different functions demonstrate that BTOA can adapt its balance between exploration and exploitation to achieve satisfactory performance across diverse optimization problems.

#### Statistical analysis

In this subsection, we conduct the Wilcoxon signed-rank test and Friedman test on the results of CEC2017 benchmarks with dimensionalities of 30, 50, and 100.

The results of the Wilcoxon signed-rank test are presented in Tables 17, 18 and 19. When the dimensionality is 30, BTOA significantly outperforms the compared algorithms in 267 out of 300 pairwise comparisons (89%), shows no significant difference in 20 comparisons (6.67%), and performs significantly worse in 13 comparisons (4.33%). For the 50-dimensional case, BTOA significantly outperforms in 260 comparisons (86.67%), shows no significant difference in 24 comparisons (8%), and performs worse in 16 comparisons (5.33%). In the 100-dimensional case, BTOA significantly outperforms in 267 comparisons (89%), shows no significant difference in 15 comparisons (5%), and performs worse in 16 comparisons (6%). These results demonstrate that BTOA’s superior performance on the majority of test functions is statistically significant, and the increase in dimensionality does not substantially affect its performance.

Table 10 presents the average rankings and overall rankings obtained from the Friedman test for the 30, 50, and 100 dimensions, all of which are statistically significant. It is evident that BTOA consistently achieves the first rank in both average and overall rankings across different dimensions. However, the rankings of the other algorithms are not stable. For 30 dimensions, the top five algorithms are BTOA, TTAO, PSA, DE, and AO. For 50 dimensions, the top five are BTOA, PSA, TTAO, DE, and AO. For 100 dimensions, the top five are BTOA, PSA, TTAO, HLOA, and DE.

**Table 10:** Friedman test on CEC2017.

	BTOA	BWO	SCA	AOA	Jaya	DE	AO	TTAO	PSA	CPO	HLOA	pvalue	Dim
Mean of rank	**2.26**	3.96	8.22	7.20	8.39	4.48	4.89	5.26	5.72	10.70	4.93	**8.02E-22**	**30**
Overall rank	**1**	2	9	8	10	3	4	6	7	11	5	**8.02E-22**	
Mean of rank	**1.83**	9.67	7.70	9.43	7.10	4.60	5.00	2.80	2.40	10.37	5.10	**1.33E-48**	**50**
Overall rank	**1**	10	8	9	7	4	5	3	2	11	6	**1.33E-48**	
Mean of rank	**1.87**	9.37	7.97	9.37	7.30	4.90	4.97	3.03	2.33	10.30	4.60	**3.37E-47**	**100**
Overall rank	**1**	9	8	10	7	5	6	3	2	11	4	**3.37E-47**

**Table 11:** Performance comparison of various algorithms in CEC2017 (F1-F15) Dim=30.

Fun.	Index	BTOA	BWO	SCA	AOA	Jaya	DE	AO	TTAO	PSA	CPO	HLOA
F1	AVE	3.82E+03	4.64E+10	1.46E+10	4.55E+10	1.08E+10	9.20E+03	2.18E+07	**2.14E+03**	5.40E+03	4.30E+10	4.90E+05
	STD	4.40E+03	3.89E+09	2.21E+09	6.47E+09	1.69E+09	3.61E+03	8.23E+06	**1.78E+03**	4.89E+03	6.12E+09	5.49E+05
F2	AVE	1.55E+13	1.50E+43	3.95E+35	4.36E+42	1.89E+34	2.22E+29	6.33E+25	9.67E+08	**1.22E+07**	8.71E+42	6.34E+13
	STD	4.70E+13	2.93E+43	7.37E+35	1.10E+43	3.65E+34	3.00E+29	2.56E+26	1.86E+09	**3.65E+07**	2.65E+43	2.16E+14
F3	AVE	1.10E+04	7.25E+04	4.89E+04	7.94E+04	1.24E+05	1.24E+05	2.69E+04	9.52E+03	1.33E+03	1.70E+05	**7.27E+02**
	STD	3.44E+03	6.16E+03	8.17E+03	6.50E+03	1.71E+04	1.64E+04	5.35E+03	2.17E+03	2.49E+03	3.61E+04	**1.87E+02**
F4	AVE	4.91E+02	1.11E+04	1.76E+03	1.09E+04	8.27E+02	4.94E+02	5.59E+02	4.94E+02	**4.82E+02**	1.07E+04	5.06E+02
	STD	2.09E+01	1.25E+03	2.99E+02	3.02E+03	8.19E+01	**3.29E+00**	3.03E+01	1.88E+01	3.49E+01	2.40E+03	3.85E+01
F5	AVE	**5.63E+02**	8.95E+02	7.85E+02	8.44E+02	7.75E+02	6.63E+02	6.67E+02	6.29E+02	6.30E+02	9.29E+02	7.52E+02
	STD	2.41E+01	2.38E+01	1.75E+01	3.70E+01	1.47E+01	**9.70E+00**	3.31E+01	2.09E+01	3.88E+01	2.56E+01	3.61E+01
F6	AVE	6.00E+02	6.85E+02	6.55E+02	6.70E+02	6.35E+02	**6.00E+02**	6.45E+02	6.12E+02	6.10E+02	6.86E+02	6.71E+02
	STD	1.77E-02	4.27E+00	5.86E+00	6.30E+00	4.48E+00	**9.47E-05**	6.76E+00	7.13E+00	6.63E+00	7.86E+00	9.46E+00
F7	AVE	**7.85E+02**	1.34E+03	1.15E+03	1.32E+03	1.12E+03	8.92E+02	1.03E+03	8.66E+02	8.88E+02	1.49E+03	1.23E+03
	STD	1.71E+01	4.81E+01	4.71E+01	6.38E+01	4.00E+01	**1.59E+01**	3.09E+01	3.31E+01	4.55E+01	8.03E+01	8.36E+01
F8	AVE	**8.59E+02**	1.13E+03	1.07E+03	1.08E+03	1.08E+03	9.63E+02	9.30E+02	9.14E+02	9.13E+02	1.17E+03	1.01E+03
	STD	1.74E+01	1.25E+01	2.12E+01	2.76E+01	1.52E+01	**1.04E+01**	3.12E+01	1.96E+01	2.69E+01	3.03E+01	4.85E+01
F9	AVE	**9.47E+02**	9.77E+03	6.12E+03	6.04E+03	6.56E+03	1.02E+03	5.14E+03	1.30E+03	3.19E+03	1.39E+04	5.54E+03
	STD	1.83E+02	7.99E+02	1.09E+03	7.18E+02	1.06E+03	**4.69E+01**	9.65E+02	4.21E+02	1.28E+03	2.01E+03	7.19E+02
F10	AVE	6.83E+03	8.20E+03	8.35E+03	6.96E+03	8.23E+03	6.88E+03	5.34E+03	5.12E+03	**4.28E+03**	9.51E+03	5.79E+03
	STD	1.54E+03	3.56E+02	**2.95E+02**	4.98E+02	3.36E+02	3.19E+02	5.31E+02	3.47E+02	6.06E+02	3.77E+02	8.01E+02
F11	AVE	**1.15E+03**	6.24E+03	2.35E+03	5.85E+03	3.46E+03	1.35E+03	1.47E+03	1.19E+03	1.24E+03	1.15E+04	1.26E+03
	STD	**2.57E+01**	8.14E+02	4.38E+02	1.77E+03	5.32E+02	3.17E+01	1.08E+02	2.85E+01	5.71E+01	2.80E+03	5.83E+01
F12	AVE	**8.70E+04**	8.94E+09	1.60E+09	9.87E+09	2.60E+08	1.54E+07	2.22E+07	2.28E+05	3.30E+05	6.61E+09	1.75E+06
	STD	**5.61E+04**	1.83E+09	3.79E+08	2.59E+09	8.06E+07	5.66E+06	1.81E+07	2.23E+05	2.49E+05	1.71E+09	1.61E+06
F13	AVE	**7.81E+03**	4.24E+09	5.90E+08	2.49E+09	4.29E+07	8.26E+05	3.57E+05	8.89E+03	1.68E+04	3.11E+09	3.86E+04
	STD	**7.82E+03**	1.55E+09	2.01E+08	1.70E+09	5.07E+07	4.13E+05	2.77E+05	8.19E+03	1.41E+04	1.22E+09	4.52E+04
F14	AVE	6.13E+03	1.69E+06	2.55E+05	4.15E+05	3.20E+05	1.05E+05	4.51E+05	5.12E+03	2.08E+04	4.15E+06	**3.27E+03**
	STD	5.91E+03	9.71E+05	1.99E+05	4.85E+05	1.55E+05	4.08E+04	4.46E+05	1.85E+03	1.78E+04	1.83E+06	**1.82E+03**
F15	AVE	8.18E+03	1.69E+08	2.57E+07	2.28E+04	1.54E+07	1.32E+05	9.50E+04	**3.22E+03**	8.37E+03	3.76E+08	1.23E+04
	STD	7.07E+03	1.03E+08	2.83E+07	8.69E+03	8.47E+06	6.65E+04	4.77E+04	**2.30E+03**	9.47E+03	2.07E+08	1.07E+04

**Table 12:** Performance comparison of various algorithms in CEC2017 (F16-F30) Dim=30.

Fun.	Index	BTOA	BWO	SCA	AOA	Jaya	DE	AO	TTAO	PSA	CPO	HLOA
F16	AVE	**2.27E+03**	4.99E+03	3.80E+03	4.47E+03	3.57E+03	2.57E+03	3.08E+03	2.57E+03	2.71E+03	5.07E+03	3.30E+03
	STD	2.56E+02	3.41E+02	2.56E+02	9.37E+02	1.74E+02	**1.59E+02**	3.54E+02	1.60E+02	3.09E+02	4.23E+02	3.60E+02
F17	AVE	**1.84E+03**	3.45E+03	2.61E+03	2.69E+03	2.49E+03	1.98E+03	2.25E+03	1.97E+03	2.23E+03	3.45E+03	2.86E+03
	STD	7.04E+01	2.92E+02	1.50E+02	3.09E+02	1.34E+02	**6.48E+01**	2.08E+02	9.42E+01	1.74E+02	2.03E+02	3.02E+02
F18	AVE	**1.40E+05**	2.19E+07	4.72E+06	4.51E+06	4.10E+06	1.15E+06	2.02E+06	1.41E+05	1.53E+05	5.17E+07	1.52E+05
	STD	8.69E+04	8.50E+06	2.76E+06	4.06E+06	2.17E+06	5.45E+05	1.59E+06	**6.55E+04**	1.54E+05	2.99E+07	1.52E+05
F19	AVE	9.20E+03	2.28E+08	3.74E+07	1.50E+06	3.13E+06	1.18E+05	1.00E+06	7.70E+03	**7.54E+03**	4.57E+08	1.12E+04
	STD	1.01E+04	9.12E+07	1.58E+07	9.51E+04	7.12E+06	5.54E+04	5.63E+05	**5.07E+03**	7.93E+03	2.23E+08	1.21E+04
F20	AVE	**2.23E+03**	2.83E+03	2.73E+03	2.81E+03	2.74E+03	2.28E+03	2.50E+03	2.39E+03	2.47E+03	3.27E+03	2.95E+03
	STD	1.12E+02	1.18E+02	1.29E+02	1.37E+02	1.06E+02	6.82E+01	1.49E+02	**6.72E+01**	1.99E+02	1.49E+02	1.97E+02
F21	AVE	**2.36E+03**	2.69E+03	2.57E+03	2.62E+03	2.56E+03	2.46E+03	2.44E+03	2.41E+03	2.40E+03	2.69E+03	2.59E+03
	STD	1.98E+01	3.22E+01	2.37E+01	5.46E+01	1.54E+01	**1.13E+01**	2.64E+01	1.76E+01	1.82E+01	2.98E+01	5.53E+01
F22	AVE	**2.30E+03**	8.00E+03	8.46E+03	8.45E+03	8.50E+03	3.94E+03	2.33E+03	2.45E+03	4.29E+03	8.17E+03	7.25E+03
	STD	**7.37E-01**	4.39E+02	2.46E+03	7.17E+02	1.89E+03	5.16E+02	8.49E+00	8.07E+02	1.95E+03	1.49E+03	1.17E+03
F23	AVE	**2.69E+03**	3.26E+03	3.02E+03	3.39E+03	2.93E+03	2.81E+03	2.90E+03	2.78E+03	2.78E+03	3.37E+03	3.05E+03
	STD	1.36E+01	4.28E+01	3.37E+01	1.24E+02	1.47E+01	**6.92E+00**	5.11E+01	2.38E+01	3.20E+01	5.78E+01	1.11E+02
F24	AVE	**2.87E+03**	3.48E+03	3.18E+03	3.70E+03	3.09E+03	3.01E+03	3.03E+03	2.93E+03	2.95E+03	3.60E+03	3.27E+03
	STD	1.61E+01	6.21E+01	2.50E+01	1.57E+02	1.13E+01	**1.10E+01**	5.90E+01	2.18E+01	3.94E+01	8.84E+01	1.52E+02
F25	AVE	**2.89E+03**	4.25E+03	3.30E+03	4.92E+03	3.06E+03	2.89E+03	2.93E+03	2.91E+03	2.90E+03	5.05E+03	2.92E+03
	STD	2.75E+00	1.66E+02	1.11E+02	6.46E+02	3.69E+01	**1.62E-01**	2.13E+01	1.87E+01	1.74E+01	5.43E+02	2.46E+01
F26	AVE	**3.90E+03**	9.97E+03	7.16E+03	9.98E+03	6.73E+03	5.25E+03	5.39E+03	4.68E+03	5.03E+03	9.86E+03	8.41E+03
	STD	5.75E+02	5.80E+02	2.60E+02	1.05E+03	1.75E+02	**6.74E+01**	1.60E+03	9.76E+02	1.07E+03	6.21E+02	1.71E+03
F27	AVE	**3.21E+03**	3.81E+03	3.44E+03	4.29E+03	3.25E+03	3.22E+03	3.31E+03	3.24E+03	3.24E+03	4.08E+03	3.45E+03
	STD	9.43E+00	1.05E+02	3.76E+01	2.47E+02	1.21E+01	**2.22E+00**	3.64E+01	9.30E+00	1.54E+01	1.85E+02	1.11E+02
F28	AVE	**3.20E+03**	6.10E+03	4.00E+03	6.60E+03	3.81E+03	3.27E+03	3.32E+03	3.22E+03	3.21E+03	6.13E+03	3.24E+03
	STD	3.20E+01	2.46E+02	2.26E+02	6.88E+02	2.88E+02	**1.40E+01**	2.95E+01	2.23E+01	1.90E+01	7.17E+02	2.26E+01
F29	AVE	**3.53E+03**	6.31E+03	4.75E+03	6.18E+03	4.48E+03	3.88E+03	4.36E+03	3.78E+03	3.78E+03	6.23E+03	5.16E+03
	STD	1.44E+02	4.66E+02	2.73E+02	9.94E+02	1.80E+02	**7.97E+01**	2.34E+02	9.81E+01	1.89E+02	4.18E+02	4.56E+02
F30	AVE	1.30E+04	6.52E+08	9.43E+07	4.31E+08	2.33E+07	1.06E+05	5.68E+06	**7.28E+03**	8.26E+03	4.62E+08	3.76E+04
	STD	4.48E+03	2.59E+08	3.87E+07	6.76E+08	1.12E+07	4.87E+04	4.39E+06	**1.64E+03**	2.13E+03	1.94E+08	2.29E+04

**Table 13:** Performance comparison of various algorithms in CEC2017 (F1-F15) Dim=50.

Fun.	Index	BTOA	BWO	SCA	AOA	Jaya	DE	AO	TTAO	PSA	CPO	HLOA
F1	AVE	**3.64E+03**	1.00E+11	5.07E+10	1.07E+11	3.47E+10	1.10E+07	2.84E+08	5.22E+05	4.36E+03	1.00E+11	1.40E+07
	STD	**3.99E+03**	3.53E+09	6.59E+09	1.14E+10	5.27E+09	1.42E+07	1.08E+08	1.03E+06	5.36E+03	9.04E+09	9.47E+06
F2	AVE	5.59E+29	3.33E+76	5.93E+67	5.66E+78	2.71E+66	3.62E+62	3.18E+50	9.18E+23	**7.39E+23**	4.20E+79	5.66E+37
	STD	3.01E+30	8.78E+76	2.30E+68	2.87E+79	1.10E+67	7.47E+62	9.91E+50	**2.87E+24**	3.91E+24	1.62E+80	2.15E+38
F3	AVE	7.81E+04	1.92E+05	1.37E+05	1.75E+05	2.76E+05	2.92E+05	1.59E+05	6.91E+04	4.14E+04	3.89E+05	**1.53E+04**
	STD	1.19E+04	2.05E+04	1.39E+04	2.13E+04	4.13E+04	2.86E+04	1.54E+04	1.15E+04	2.04E+04	8.02E+04	**6.43E+03**
F4	AVE	**5.36E+02**	2.98E+04	8.67E+03	3.19E+04	3.65E+03	6.43E+02	7.71E+02	5.84E+02	5.44E+02	2.98E+04	6.34E+02
	STD	4.23E+01	3.31E+03	1.91E+03	5.02E+03	6.42E+02	**1.46E+01**	6.81E+01	5.37E+01	6.01E+01	4.44E+03	6.25E+01
F5	AVE	**6.71E+02**	1.17E+03	1.07E+03	1.13E+03	1.07E+03	8.79E+02	8.40E+02	8.19E+02	7.56E+02	1.24E+03	9.01E+02
	STD	5.04E+01	1.98E+01	4.13E+01	4.06E+01	2.70E+01	**1.65E+01**	3.59E+01	5.26E+01	4.37E+01	4.49E+01	6.04E+01
F6	AVE	6.00E+02	6.99E+02	6.73E+02	6.89E+02	6.58E+02	**6.00E+02**	6.59E+02	6.32E+02	6.21E+02	7.04E+02	6.74E+02
	STD	3.63E-01	3.86E+00	5.77E+00	7.13E+00	5.09E+00	**1.37E-02**	5.52E+00	8.49E+00	7.80E+00	9.38E+00	6.85E+00
F7	AVE	**9.01E+02**	1.91E+03	1.67E+03	1.93E+03	1.60E+03	1.14E+03	1.45E+03	1.05E+03	1.15E+03	2.26E+03	1.75E+03
	STD	3.26E+01	4.46E+01	8.50E+01	5.80E+01	5.20E+01	**2.16E+01**	9.91E+01	7.17E+01	7.68E+01	1.94E+02	1.02E+02
F8	AVE	**9.43E+02**	1.48E+03	1.40E+03	1.47E+03	1.40E+03	1.18E+03	1.14E+03	1.11E+03	1.06E+03	1.55E+03	1.22E+03
	STD	4.05E+01	2.23E+01	3.65E+01	3.37E+01	3.12E+01	**1.73E+01**	4.28E+01	4.34E+01	5.31E+01	4.25E+01	6.79E+01
F9	AVE	**2.42E+03**	3.48E+04	2.56E+04	2.60E+04	2.04E+04	3.96E+03	2.10E+04	9.29E+03	9.71E+03	4.85E+04	1.61E+04
	STD	2.96E+03	2.12E+03	3.48E+03	3.45E+03	4.68E+03	**5.41E+02**	4.11E+03	3.37E+03	2.50E+03	6.89E+03	2.26E+03
F10	AVE	1.22E+04	1.43E+04	1.49E+04	1.33E+04	1.49E+04	1.32E+04	8.77E+03	9.49E+03	**6.94E+03**	1.62E+04	9.64E+03
	STD	2.36E+03	4.90E+02	4.36E+02	7.67E+02	4.04E+02	**3.72E+02**	1.06E+03	5.14E+02	6.05E+02	4.93E+02	1.25E+03
F11	AVE	**1.27E+03**	1.96E+04	8.39E+03	2.02E+04	1.04E+04	2.60E+03	1.78E+03	1.28E+03	1.36E+03	3.08E+04	1.39E+03
	STD	4.74E+01	1.78E+03	1.59E+03	3.43E+03	2.15E+03	2.34E+02	1.26E+02	**3.14E+01**	6.08E+01	6.60E+03	8.66E+01
F12	AVE	**2.12E+06**	5.49E+10	1.57E+10	6.33E+10	6.94E+09	2.59E+08	2.41E+08	2.40E+06	2.18E+06	4.37E+10	1.96E+07
	STD	1.22E+06	7.08E+09	3.23E+09	1.27E+10	9.11E+08	6.14E+07	1.80E+08	**8.31E+05**	1.27E+06	8.43E+09	1.12E+07
F13	AVE	8.94E+03	3.03E+10	3.74E+09	2.80E+10	1.41E+09	3.06E+06	2.75E+06	**6.29E+03**	6.57E+03	2.12E+10	7.15E+04
	STD	9.70E+03	6.19E+09	1.69E+09	1.14E+10	4.58E+08	1.56E+06	1.01E+06	**3.64E+03**	5.69E+03	5.12E+09	4.63E+04
F14	AVE	**8.80E+04**	3.26E+07	3.54E+06	1.71E+07	2.03E+06	1.28E+06	1.86E+06	1.05E+05	1.00E+05	3.86E+07	2.05E+05
	STD	**5.54E+04**	1.21E+07	1.49E+06	2.24E+07	9.16E+05	4.82E+05	1.06E+06	6.16E+04	7.06E+04	1.74E+07	5.49E+05
F15	AVE	1.04E+04	4.06E+09	5.56E+08	1.31E+09	3.92E+08	4.19E+05	3.49E+05	**6.95E+03**	1.09E+04	3.92E+09	2.14E+04
	STD	7.29E+03	1.20E+09	2.31E+08	1.70E+09	1.24E+08	3.09E+05	1.23E+05	**4.13E+03**	7.06E+03	1.49E+09	1.42E+04

**Table 14:** Performance comparison of various algorithms in CEC2017 (F16-F30) Dim=50.

Fun.	Index	BTOA	BWO	SCA	AOA	Jaya	DE	AO	TTAO	PSA	CPO	HLOA
F16	AVE	**3.08E+03**	8.15E+03	5.75E+03	7.28E+03	5.70E+03	4.35E+03	4.20E+03	3.25E+03	3.49E+03	7.72E+03	4.37E+03
	STD	3.63E+02	4.12E+02	3.06E+02	1.41E+03	3.06E+02	**1.94E+02**	5.77E+02	2.54E+02	4.80E+02	4.91E+02	6.67E+02
F17	AVE	**2.63E+03**	6.00E+03	4.48E+03	6.50E+03	4.49E+03	3.19E+03	3.55E+03	2.88E+03	3.24E+03	6.56E+03	3.90E+03
	STD	2.81E+02	6.83E+02	3.33E+02	1.47E+03	2.18E+02	**1.45E+02**	3.83E+02	2.03E+02	3.11E+02	6.15E+02	3.72E+02
F18	AVE	1.12E+06	8.91E+07	2.35E+07	8.04E+07	2.38E+07	7.95E+06	5.88E+06	8.18E+05	7.31E+05	1.45E+08	**4.50E+05**
	STD	8.15E+05	2.18E+07	1.09E+07	3.57E+07	7.54E+06	2.47E+06	3.64E+06	**3.45E+05**	4.05E+05	5.95E+07	3.78E+05
F19	AVE	2.17E+04	2.48E+09	3.74E+08	1.15E+09	7.91E+07	1.47E+05	1.79E+06	1.86E+04	1.68E+04	1.66E+09	**1.56E+04**
	STD	1.22E+04	8.07E+08	1.24E+08	9.62E+08	6.29E+07	5.97E+04	1.20E+06	**6.32E+03**	9.57E+03	6.11E+08	1.14E+04
F20	AVE	3.09E+03	3.92E+03	4.04E+03	3.56E+03	4.11E+03	3.26E+03	3.28E+03	**2.96E+03**	3.16E+03	4.84E+03	3.72E+03
	STD	4.26E+02	**1.21E+02**	1.90E+02	2.85E+02	1.58E+02	1.53E+02	2.58E+02	1.42E+02	3.03E+02	1.79E+02	3.44E+02
F21	AVE	**2.40E+03**	3.13E+03	2.89E+03	3.05E+03	2.84E+03	2.70E+03	2.66E+03	2.57E+03	2.56E+03	3.12E+03	2.95E+03
	STD	2.27E+01	5.55E+01	4.91E+01	6.18E+01	2.70E+01	**1.45E+01**	5.69E+01	3.30E+01	5.90E+01	4.31E+01	1.16E+02
F22	AVE	1.35E+04	1.62E+04	1.65E+04	1.55E+04	1.61E+04	1.48E+04	9.56E+03	9.68E+03	**8.77E+03**	1.79E+04	1.15E+04
	STD	3.24E+03	5.34E+02	4.77E+02	4.79E+02	4.50E+02	7.62E+02	2.65E+03	3.32E+03	1.48E+03	**4.23E+02**	1.14E+03
F23	AVE	**2.84E+03**	4.00E+03	3.57E+03	4.29E+03	3.36E+03	3.11E+03	3.33E+03	3.03E+03	3.03E+03	4.20E+03	3.77E+03
	STD	3.47E+01	7.69E+01	5.14E+01	2.40E+02	3.44E+01	**1.67E+01**	9.09E+01	5.94E+01	7.32E+01	1.63E+02	1.89E+02
F24	AVE	**3.01E+03**	4.36E+03	3.75E+03	4.75E+03	3.46E+03	3.31E+03	3.45E+03	3.18E+03	3.19E+03	4.63E+03	3.83E+03
	STD	3.67E+01	1.05E+02	7.10E+01	2.19E+02	3.20E+01	**1.39E+01**	1.04E+02	3.10E+01	7.38E+01	1.91E+02	1.48E+02
F25	AVE	**3.05E+03**	1.31E+04	7.04E+03	1.44E+04	4.50E+03	3.06E+03	3.29E+03	3.13E+03	3.07E+03	1.51E+04	3.10E+03
	STD	3.34E+01	5.44E+02	5.66E+02	1.46E+03	3.52E+02	**7.82E+00**	7.25E+01	2.62E+01	3.12E+01	1.75E+03	3.88E+01
F26	AVE	**3.29E+03**	1.62E+04	1.22E+04	1.65E+04	1.06E+04	7.53E+03	7.20E+03	8.40E+03	7.17E+03	1.76E+04	1.33E+04
	STD	7.92E+02	4.36E+02	8.30E+02	9.40E+02	3.80E+02	**1.44E+02**	2.79E+03	1.65E+03	1.52E+03	9.37E+02	1.33E+03
F27	AVE	**3.29E+03**	5.69E+03	4.57E+03	6.62E+03	3.69E+03	3.45E+03	3.84E+03	3.56E+03	3.52E+03	6.51E+03	4.30E+03
	STD	3.17E+01	2.97E+02	1.65E+02	5.57E+02	5.93E+01	**2.04E+01**	1.88E+02	6.28E+01	1.11E+02	4.00E+02	3.69E+02
F28	AVE	**3.30E+03**	1.16E+04	7.24E+03	1.14E+04	8.31E+03	3.36E+03	3.85E+03	3.39E+03	3.33E+03	1.18E+04	3.37E+03
	STD	3.07E+01	4.40E+02	6.70E+02	1.08E+03	1.09E+03	**2.33E+01**	2.24E+02	3.03E+01	2.60E+01	1.02E+03	4.18E+01
F29	AVE	**3.92E+03**	1.77E+04	7.80E+03	2.21E+04	6.66E+03	4.92E+03	5.99E+03	4.77E+03	4.39E+03	1.64E+04	6.57E+03
	STD	2.82E+02	4.56E+03	6.29E+02	8.27E+03	4.86E+02	**1.82E+02**	6.24E+02	3.03E+02	3.50E+02	7.33E+03	7.29E+02
F30	AVE	9.90E+05	3.71E+09	8.08E+08	3.31E+09	2.27E+08	9.06E+06	8.01E+07	**9.08E+05**	1.03E+06	3.21E+09	3.59E+06
	STD	2.41E+05	8.52E+08	1.96E+08	2.27E+09	1.03E+08	1.92E+06	2.43E+07	**9.75E+04**	2.26E+05	7.87E+08	1.82E+06

**Table 15:** Performance comparison of various algorithms in CEC2017 (F1-F15) Dim=100.

Fun.	Index	BTOA	BWO	SCA	AOA	Jaya	DE	AO	TTAO	PSA	CPO	HLOA
F1	AVE	**2.83E+05**	2.47E+11	1.80E+11	2.69E+11	1.22E+11	3.50E+08	8.38E+09	2.69E+07	3.02E+06	2.72E+11	4.08E+08
	STD	**4.15E+05**	7.82E+09	1.20E+10	1.17E+10	1.24E+10	1.30E+08	1.55E+09	7.54E+06	4.31E+06	1.91E+10	1.54E+08
F2	AVE	**2.71E+87**	3.41E+168	1.98E+158	1.28E+166	5.28E+156	2.80E+151	2.46E+135	1.97E+104	4.37E+87	3.84E+175	1.32E+118
	STD	9.89E+87	**6.55E+04**	**6.55E+04**	**6.55E+04**	**6.55E+04**	6.18E+151	1.28E+136	7.60E+104	2.11E+88	**6.55E+04**	7.11E+118
F3	AVE	2.88E+05	3.37E+05	3.96E+05	3.36E+05	7.85E+05	7.52E+05	3.16E+05	3.80E+05	3.92E+05	1.21E+06	**1.37E+05**
	STD	1.39E+04	**1.25E+04**	3.44E+04	1.52E+04	6.38E+04	5.97E+04	1.50E+04	4.05E+04	1.19E+05	1.42E+06	2.00E+04
F4	AVE	**7.35E+02**	8.80E+04	3.60E+04	8.18E+04	1.84E+04	1.06E+03	2.38E+03	9.46E+02	8.17E+02	8.95E+04	1.02E+03
	STD	**4.56E+01**	6.31E+03	5.82E+03	1.22E+04	2.83E+03	5.74E+01	3.84E+02	6.79E+01	5.62E+01	1.24E+04	8.18E+01
F5	AVE	**1.02E+03**	2.08E+03	1.94E+03	2.03E+03	1.87E+03	1.56E+03	1.44E+03	1.44E+03	1.18E+03	2.22E+03	1.54E+03
	STD	1.16E+02	**3.14E+01**	5.70E+01	6.90E+01	4.34E+01	3.58E+01	6.18E+01	1.07E+02	9.01E+01	5.58E+01	7.81E+01
F6	AVE	6.11E+02	7.10E+02	6.97E+02	7.06E+02	6.88E+02	**6.08E+02**	6.76E+02	6.59E+02	6.42E+02	7.21E+02	6.81E+02
	STD	4.08E+00	2.73E+00	3.72E+00	3.03E+00	6.96E+00	**4.64E-01**	4.24E+00	9.37E+00	6.32E+00	4.58E+00	6.76E+00
F7	AVE	**1.37E+03**	3.82E+03	3.73E+03	3.88E+03	3.53E+03	1.96E+03	3.06E+03	1.71E+03	2.25E+03	4.39E+03	3.45E+03
	STD	1.14E+02	5.38E+01	1.34E+02	6.23E+01	2.04E+02	**1.93E+01**	2.10E+02	1.48E+02	1.91E+02	3.24E+02	1.56E+02
F8	AVE	**1.28E+03**	2.55E+03	2.31E+03	2.47E+03	2.25E+03	1.85E+03	1.88E+03	1.79E+03	1.56E+03	2.66E+03	1.96E+03
	STD	6.93E+01	3.41E+01	5.61E+01	9.41E+01	6.58E+01	**2.39E+01**	9.66E+01	1.09E+02	9.01E+01	7.71E+01	1.01E+02
F9	AVE	4.76E+04	7.58E+04	7.85E+04	6.58E+04	7.50E+04	3.46E+04	5.14E+04	4.50E+04	**2.64E+04**	1.14E+05	3.78E+04
	STD	1.46E+04	3.31E+03	6.80E+03	4.81E+03	1.20E+04	3.95E+03	5.13E+03	5.85E+03	3.63E+03	1.08E+04	**2.86E+03**
F10	AVE	2.93E+04	3.13E+04	3.22E+04	2.98E+04	3.21E+04	3.10E+04	1.99E+04	2.34E+04	**1.53E+04**	3.40E+04	2.22E+04
	STD	2.98E+03	**5.53E+02**	5.82E+02	9.31E+02	5.79E+02	6.34E+02	1.92E+03	7.41E+02	1.53E+03	5.87E+02	2.10E+03
F11	AVE	1.26E+04	2.77E+05	1.07E+05	1.69E+05	2.16E+05	1.60E+05	1.35E+05	8.65E+03	**4.01E+03**	3.43E+05	5.26E+03
	STD	2.15E+03	4.06E+04	1.74E+04	2.32E+04	3.93E+04	2.37E+04	2.81E+04	1.11E+03	1.02E+03	6.29E+04	**9.76E+02**
F12	AVE	**1.43E+07**	1.74E+11	7.45E+10	1.82E+11	3.84E+10	2.72E+09	1.59E+09	4.29E+07	2.14E+07	1.60E+11	3.26E+08
	STD	**8.69E+06**	1.20E+10	9.38E+09	2.48E+10	5.97E+09	3.37E+08	5.19E+08	1.61E+07	1.13E+07	2.00E+10	1.17E+08
F13	AVE	**7.39E+03**	3.83E+10	1.21E+10	4.11E+10	5.25E+09	2.68E+05	1.08E+07	1.38E+04	1.03E+04	3.34E+10	1.02E+06
	STD	**5.14E+03**	3.74E+09	2.19E+09	5.15E+09	6.60E+08	4.39E+05	4.30E+06	8.01E+03	6.44E+03	5.42E+09	2.91E+06
F14	AVE	**7.56E+05**	5.57E+07	3.52E+07	6.43E+07	4.39E+07	2.42E+07	9.34E+06	8.61E+05	1.17E+06	1.32E+08	9.50E+05
	STD	4.15E+05	1.52E+07	1.53E+07	2.66E+07	1.21E+07	6.47E+06	3.64E+06	**3.38E+05**	5.07E+05	5.26E+07	4.48E+05
F15	AVE	5.48E+03	1.81E+10	4.16E+09	2.02E+10	2.27E+09	1.25E+06	2.29E+06	**3.93E+03**	5.14E+03	1.44E+10	4.93E+04
	STD	4.40E+03	2.46E+09	9.52E+08	4.05E+09	6.77E+08	7.13E+05	8.04E+05	**1.94E+03**	3.42E+03	3.37E+09	1.58E+04

**Table 16:** Performance comparison of various algorithms in CEC2017 (F16-F30) Dim=100.

**Fun.**	**Index**	**BTOA**	**BWO**	**SCA**	**AOA**	**Jaya**	**DE**	**AO**	**TTAO**	**PSA**	**CPO**	**HLOA**
F16	AVE	**5.49E+03**	2.04E+04	1.35E+04	1.95E+04	1.24E+04	1.07E+04	8.47E+03	6.74E+03	5.94E+03	1.95E+04	8.14E+03
	STD	6.39E+02	1.35E+03	1.00E+03	3.07E+03	3.65E+02	**2.65E+02**	9.78E+02	6.25E+02	7.19E+02	2.01E+03	1.73E+03
F17	AVE	**4.56E+03**	2.03E+06	1.91E+04	2.48E+06	1.40E+04	7.30E+03	6.69E+03	4.98E+03	5.31E+03	1.07E+06	6.91E+03
	STD	6.98E+02	1.08E+06	1.19E+04	2.38E+06	1.71E+03	**2.40E+02**	7.01E+02	5.06E+02	5.61E+02	8.75E+05	6.64E+02
F18	AVE	1.81E+06	1.20E+08	6.40E+07	1.14E+08	7.35E+07	4.99E+07	6.52E+06	1.70E+06	1.75E+06	2.36E+08	**1.41E+06**
	STD	1.04E+06	3.63E+07	2.75E+07	5.97E+07	2.61E+07	1.18E+07	2.67E+06	9.34E+05	8.92E+05	8.60E+07	**5.87E+05**
F19	AVE	6.41E+03	1.90E+10	3.38E+09	2.02E+10	2.30E+09	1.93E+06	1.55E+07	**4.99E+03**	5.86E+03	1.42E+10	2.67E+05
	STD	5.56E+03	2.30E+09	7.66E+08	4.19E+09	4.55E+08	1.60E+06	1.11E+07	**2.27E+03**	3.58E+03	2.80E+09	2.17E+05
F20	AVE	6.15E+03	7.36E+03	7.67E+03	6.91E+03	7.65E+03	7.21E+03	5.48E+03	**5.19E+03**	5.39E+03	8.64E+03	6.48E+03
	STD	1.03E+03	3.39E+02	2.24E+02	4.45E+02	2.82E+02	**1.32E+02**	6.23E+02	2.22E+02	5.19E+02	3.62E+02	6.18E+02
F21	AVE	**2.66E+03**	4.64E+03	4.03E+03	4.59E+03	3.71E+03	3.41E+03	3.70E+03	3.09E+03	3.07E+03	4.58E+03	4.31E+03
	STD	6.22E+01	9.30E+01	8.72E+01	1.68E+02	6.76E+01	**1.94E+01**	2.00E+02	1.02E+02	1.11E+02	1.43E+02	2.20E+02
F22	AVE	2.59E+04	3.40E+04	3.46E+04	3.27E+04	3.41E+04	3.31E+04	2.36E+04	2.61E+04	**1.84E+04**	3.69E+04	2.49E+04
	STD	9.71E+03	**4.66E+02**	5.94E+02	1.14E+03	7.76E+02	5.18E+02	1.31E+03	8.23E+02	9.80E+02	5.65E+02	2.47E+03
F23	AVE	**3.17E+03**	5.94E+03	4.98E+03	6.81E+03	4.35E+03	3.71E+03	4.42E+03	3.55E+03	3.49E+03	6.68E+03	5.10E+03
	STD	5.58E+01	1.36E+02	9.91E+01	3.57E+02	8.52E+01	**2.79E+01**	1.98E+02	1.22E+02	9.16E+01	2.63E+02	2.90E+02
F24	AVE	**3.63E+03**	8.69E+03	6.86E+03	1.09E+04	5.13E+03	4.26E+03	5.40E+03	4.07E+03	4.15E+03	1.04E+04	7.49E+03
	STD	8.56E+01	2.98E+02	2.38E+02	8.66E+02	1.10E+02	**1.88E+01**	2.65E+02	1.07E+02	1.69E+02	6.97E+02	7.04E+02
F25	AVE	**3.39E+03**	2.57E+04	1.77E+04	2.73E+04	1.42E+04	4.39E+03	4.54E+03	3.64E+03	3.47E+03	3.04E+04	3.69E+03
	STD	**5.15E+01**	1.33E+03	2.12E+03	2.30E+03	1.71E+03	9.16E+01	2.27E+02	6.08E+01	7.15E+01	3.04E+03	6.49E+01
F26	AVE	**8.53E+03**	4.89E+04	3.66E+04	4.97E+04	2.62E+04	1.61E+04	2.54E+04	1.90E+04	1.69E+04	5.26E+04	3.43E+04
	STD	2.15E+03	1.25E+03	2.39E+03	2.66E+03	1.10E+03	**2.91E+02**	4.21E+03	3.77E+03	2.56E+03	3.19E+03	5.01E+03
F27	AVE	**3.50E+03**	1.13E+04	7.71E+03	1.19E+04	5.01E+03	4.08E+03	4.65E+03	4.20E+03	3.76E+03	1.18E+04	4.73E+03
	STD	**4.68E+01**	6.39E+02	4.80E+02	1.17E+03	2.94E+02	7.52E+01	3.45E+02	1.43E+02	1.18E+02	1.03E+03	7.17E+02
F28	AVE	**3.49E+03**	2.66E+04	2.26E+04	3.33E+04	2.40E+04	5.24E+03	5.62E+03	3.75E+03	3.60E+03	3.30E+04	3.68E+03
	STD	**3.43E+01**	4.94E+02	2.00E+03	2.95E+03	1.48E+03	5.07E+02	5.56E+02	3.65E+01	4.68E+01	3.00E+03	6.55E+01
F29	AVE	**6.53E+03**	2.48E+05	2.11E+04	3.27E+05	1.70E+04	9.44E+03	1.11E+04	7.67E+03	7.01E+03	2.29E+05	1.17E+04
	STD	7.01E+02	8.64E+04	3.90E+03	2.26E+05	1.57E+03	**2.62E+02**	1.25E+03	4.78E+02	3.92E+02	1.21E+05	1.22E+03
F30	AVE	**1.48E+04**	3.40E+10	8.30E+09	3.68E+10	3.73E+09	2.87E+06	1.70E+08	1.97E+05	3.31E+04	2.57E+10	1.20E+07
	STD	**5.42E+03**	3.50E+09	1.32E+09	7.66E+09	5.12E+08	5.78E+05	8.08E+07	1.24E+05	2.03E+04	5.03E+09	9.30E+06

**Table 17:** Wilcoxon signed-rank test on CEC2017 (Dim=30).

BTOA vs	BWO		SCA		AOA		Jaya		DE		AO		TTAO		PSA		CPO		HLOA	
	p	win	p	win	p	win	p	win	p	win	p	win	p	win	p	win	p	win	p	win
**F1**	1.00E+00	=	1.73E-06	+	2.50E-01	=	1.73E-06	+	1.73E-06	+	1.73E-06	+	1.73E-06	+	1.73E-06	+	1.73E-06	+	1.00E+00	=
**F2**	1.73E-06	+	1.73E-06	+	1.00E+00	=	1.73E-06	+	1.73E-06	+	1.73E-06	+	1.73E-06	+	1.73E-06	+	1.73E-06	+	1.73E-06	+
**F3**	1.00E+00	=	1.73E-06	+	7.81E-03	+	1.73E-06	+	1.73E-06	+	1.73E-06	+	1.73E-06	+	1.73E-06	+	1.73E-06	+	1.00E+00	=
**F4**	1.73E-06	+	1.73E-06	+	1.23E-05	+	1.73E-06	+	1.73E-06	+	1.73E-06	+	1.73E-06	+	1.73E-06	+	1.73E-06	+	5.96E-05	+
**F5**	1.73E-06	-	1.73E-06	+	1.73E-06	+	1.73E-06	+	1.73E-06	+	1.73E-06	-	3.52E-06	+	1.11E-02	+	1.73E-06	+	3.59E-04	+
**F6**	1.73E-06	+	1.73E-06	+	1.73E-06	+	1.73E-06	+	1.00E+00	=	1.73E-06	+	2.50E-01	=	1.00E+00	=	1.73E-06	+	1.73E-06	+
**F7**	8.31E-04	-	1.73E-06	+	1.97E-05	-	1.73E-06	+	1.73E-06	+	1.06E-04	-	1.73E-06	+	1.73E-06	+	1.73E-06	+	8.94E-01	=
**F8**	1.00E+00	=	1.73E-06	+	1.73E-06	+	1.73E-06	+	1.73E-06	+	1.73E-06	+	1.73E-06	+	1.73E-06	+	1.73E-06	+	1.73E-06	+
**F9**	1.00E+00	=	1.73E-06	+	1.00E+00	=	1.73E-06	+	1.73E-06	+	1.00E+00	=	1.73E-06	+	1.73E-06	+	1.73E-06	+	1.00E+00	=
**F10**	1.00E+00	=	1.73E-06	+	1.00E+00	=	1.73E-06	+	1.73E-06	+	1.00E+00	=	1.73E-06	+	1.73E-06	+	1.73E-06	+	1.00E+00	=
**F11**	1.00E+00	=	1.73E-06	+	3.79E-06	+	1.73E-06	+	1.73E-06	+	1.00E+00	=	1.73E-06	+	1.73E-06	+	1.73E-06	+	1.00E+00	=
**F12**	1.73E-06	+	1.73E-06	+	1.73E-06	+	1.73E-06	+	1.73E-06	+	1.73E-06	+	1.73E-06	+	1.73E-06	+	1.73E-06	+	1.73E-06	+
**F13**	1.73E-06	+	1.73E-06	+	1.73E-06	+	1.73E-06	+	1.73E-06	+	1.73E-06	+	1.73E-06	+	1.73E-06	+	1.73E-06	+	1.73E-06	+
**F14**	4.18E-07	+	1.73E-06	+	1.73E-06	+	1.73E-06	+	1.00E+00	=	1.73E-06	+	1.00E+00	=	5.00E-01	=	1.73E-06	+	1.64E-05	+
**F15**	1.73E-06	+	1.73E-06	+	1.73E-06	+	1.73E-06	+	1.73E-06	+	1.73E-06	+	1.73E-06	+	2.09E-06	+	1.73E-06	+	1.45E-06	+
**F16**	1.73E-06	+	1.73E-06	+	1.73E-06	+	1.73E-06	+	1.00E+00	=	1.73E-06	+	1.00E+00	=	1.00E+00	=	1.73E-06	+	1.00E+00	=
**F17**	1.73E-06	+	1.73E-06	+	1.73E-06	+	1.23E-05	+	1.00E+00	=	1.73E-06	+	1.00E+00	=	1.00E+00	=	1.73E-06	+	1.00E+00	=
**F18**	1.73E-06	+	1.73E-06	+	1.73E-06	+	1.73E-06	+	1.80E-01	=	1.73E-06	+	1.00E+00	=	1.25E-01	=	1.73E-06	+	2.59E-06	+
**F19**	1.73E-06	+	1.73E-06	+	1.73E-06	+	1.00E+00	=	1.00E+00	=	1.73E-06	+	1.00E+00	=	1.00E+00	=	1.73E-06	+	1.00E+00	=
**F20**	6.44E-01	=	2.13E-06	+	8.47E-06	+	3.78E-02	+	4.88E-04	-	2.77E-03	+	4.88E-04	-	5.81E-01	=	1.92E-06	+	2.23E-02	+
**F21**	1.73E-06	+	1.73E-06	+	1.73E-06	+	1.73E-06	+	5.00E-01	=	1.73E-06	+	1.00E+00	=	1.95E-03	+	1.73E-06	+	6.25E-02	=
**F22**	1.73E-06	+	1.73E-06	+	1.73E-06	+	1.23E-05	+	1.00E+00	=	1.73E-06	+	1.00E+00	=	9.77E-04	+	1.73E-06	+	3.91E-03	+
**F23**	1.73E-06	+	1.73E-06	+	1.73E-06	+	8.30E-06	+	1.00E+00	=	1.73E-06	+	1.00E+00	=	4.88E-04	+	1.73E-06	+	1.95E-03	+

**Table 18:** Wilcoxon signed-rank test on CEC2017 (Dim=50).

BTOA vs	BWO		SCA		AOA		Jaya		DE		AO		TTAO		PSA		CPO		HLOA	
	p	win	p	win	p	win	p	win	p	win	p	win	p	win	p	win	p	win	p	win
**F1**	1.73E-06	+	1.73E-06	+	1.73E-06	+	1.73E-06	+	1.73E-06	+	1.73E-06	+	1.73E-06	+	9.26E-01	=	1.73E-06	+	1.73E-06	+
**F2**	1.73E-06	+	1.73E-06	+	1.73E-06	+	1.73E-06	+	1.73E-06	+	1.73E-06	+	8.94E-04	-	3.11E-05	-	1.73E-06	+	1.73E-06	+
**F3**	1.73E-06	+	1.73E-06	+	1.73E-06	+	1.73E-06	+	1.73E-06	+	1.73E-06	+	9.84E-03	-	3.18E-06	-	1.73E-06	+	1.73E-06	-
**F4**	1.73E-06	+	1.73E-06	+	1.73E-06	+	1.73E-06	+	1.73E-06	+	1.73E-06	+	2.61E-04	+	6.73E-01	=	1.73E-06	+	4.73E-06	+
**F5**	1.73E-06	+	1.73E-06	+	1.73E-06	+	1.73E-06	+	1.73E-06	+	1.92E-06	+	2.35E-06	+	5.79E-05	+	1.73E-06	+	1.73E-06	+
**F6**	1.73E-06	+	1.73E-06	+	1.73E-06	+	1.73E-06	+	3.18E-06	-	1.73E-06	+	1.73E-06	+	1.73E-06	+	1.73E-06	+	1.73E-06	+
**F7**	1.73E-06	+	1.73E-06	+	1.73E-06	+	1.73E-06	+	1.73E-06	+	1.73E-06	+	2.88E-06	+	1.73E-06	+	1.73E-06	+	1.73E-06	+
**F8**	1.73E-06	+	1.73E-06	+	1.73E-06	+	1.73E-06	+	1.73E-06	+	1.73E-06	+	1.73E-06	+	3.52E-06	+	1.73E-06	+	1.73E-06	+
**F9**	1.73E-06	+	1.73E-06	+	1.73E-06	+	1.73E-06	+	4.53E-04	+	1.73E-06	+	6.98E-06	+	1.13E-05	+	1.73E-06	+	1.92E-06	+
**F10**	7.69E-06	+	1.73E-06	+	4.72E-02	+	2.35E-06	+	9.78E-02	=	6.98E-06	-	8.92E-05	-	1.92E-06	-	1.73E-06	+	1.36E-04	-
**F11**	1.73E-06	+	1.73E-06	+	1.73E-06	+	1.73E-06	+	1.73E-06	+	1.73E-06	+	3.39E-01	=	1.02E-05	+	1.73E-06	+	6.34E-06	+
**F12**	1.73E-06	+	1.73E-06	+	1.73E-06	+	1.73E-06	+	1.73E-06	+	1.73E-06	+	2.89E-01	=	9.75E-01	=	1.73E-06	+	1.73E-06	+
**F13**	1.73E-06	+	1.73E-06	+	1.73E-06	+	1.73E-06	+	1.73E-06	+	1.73E-06	+	9.92E-01	=	6.44E-01	=	1.73E-06	+	1.92E-06	+
**F14**	1.73E-06	+	1.73E-06	+	1.73E-06	+	1.73E-06	+	1.73E-06	+	1.73E-06	+	3.71E-01	=	5.72E-01	=	1.73E-06	+	7.81E-01	=
**F15**	1.73E-06	+	1.73E-06	+	1.73E-06	+	1.73E-06	+	1.73E-06	+	1.73E-06	+	3.16E-02	-	7.34E-01	=	1.73E-06	+	2.41E-03	+
**F16**	1.73E-06	+	1.73E-06	+	1.73E-06	+	1.73E-06	+	1.92E-06	+	2.35E-06	+	4.28E-02	+	1.71E-03	+	1.73E-06	+	1.92E-06	+
**F17**	1.73E-06	+	1.73E-06	+	1.73E-06	+	1.73E-06	+	3.88E-06	+	1.73E-06	+	1.48E-03	+	1.92E-06	+	1.73E-06	+	1.73E-06	+
**F18**	1.73E-06	+	1.73E-06	+	1.73E-06	+	1.73E-06	+	1.73E-06	+	1.73E-06	+	3.93E-01	=	1.25E-01	=	1.73E-06	+	1.48E-04	-
**F19**	1.73E-06	+	1.73E-06	+	1.73E-06	+	1.73E-06	+	1.73E-06	+	1.73E-06	+	3.09E-01	=	1.16E-01	=	1.73E-06	+	5.45E-02	=
**F20**	2.35E-06	+	1.73E-06	+	2.83E-04	+	1.73E-06	+	9.78E-02	=	4.95E-02	+	1.71E-01	=	3.49E-01	=	1.73E-06	+	8.47E-06	+
**F21**	1.73E-06	+	1.73E-06	+	1.73E-06	+	1.73E-06	+	1.73E-06	+	1.73E-06	+	1.73E-06	+	1.73E-06	+	1.73E-06	+	1.73E-06	+
**F22**	4.45E-05	+	5.22E-06	+	4.11E-03	+	1.80E-05	+	2.13E-01	=	8.92E-05	-	1.15E-04	-	1.64E-05	-	1.73E-06	+	1.38E-03	-
**F23**	1.73E-06	+	1.73E-06	+	1.73E-06	+	1.73E-06	+	1.73E-06	+	1.73E-06	+	1.73E-06	+	1.73E-06	+	1.73E-06	+	1.73E-06	+
**F24**	1.73E-06	+	1.73E-06	+	1.73E-06	+	1.73E-06	+	1.73E-06	+	1.73E-06	+	1.73E-06	+	1.73E-06	+	1.73E-06	+	1.73E-06	+
**F25**	1.73E-06	+	1.73E-06	+	1.73E-06	+	1.73E-06	+	3.60E-01	=	1.73E-06	+	1.92E-06	+	3.00E-02	+	1.73E-06	+	2.05E-04	+
**F26**	1.73E-06	+	1.73E-06	+	1.73E-06	+	1.73E-06	+	1.73E-06	+	2.35E-06	+	1.73E-06	+	1.73E-06	+	1.73E-06	+	1.73E-06	+
**F27**	1.73E-06	+	1.73E-06	+	1.73E-06	+	1.73E-06	+	1.73E-06	+	1.73E-06	+	1.73E-06	+	1.73E-06	+	1.73E-06	+	1.73E-06	+
**F28**	1.73E-06	+	1.73E-06	+	1.73E-06	+	1.73E-06	+	5.22E-06	+	1.73E-06	+	2.35E-06	+	1.38E-03	+	1.73E-06	+	5.22E-06	+
**F29**	1.73E-06	+	1.73E-06	+	1.73E-06	+	1.73E-06	+	1.92E-06	+	1.73E-06	+	1.73E-06	+	1.80E-05	+	1.73E-06	+	1.73E-06	+
**F30**	1.73E-06	+	1.73E-06	+	1.73E-06	+	1.73E-06	+	1.73E-06	+	1.73E-06	+	1.99E-01	=	3.39E-01	=	1.73E-06	+	1.73E-06	+

**Table 19:** Wilcoxon signed-rank test on CEC2017 (Dim=100).

BTOA vs	BWO		SCA		AOA		Jaya		DE		AO		TTAO		PSA		CPO		HLOA	
	p	win	p	win	p	win	p	win	p	win	p	win	p	win	p	win	p	win	p	win
**F1**	1.73E-06	+	1.73E-06	+	1.73E-06	+	1.73E-06	+	1.73E-06	+	1.73E-06	+	1.73E-06	+	2.35E-06	+	1.73E-06	+	1.73E-06	+
**F2**	1.73E-06	+	1.73E-06	+	1.73E-06	+	1.73E-06	+	1.73E-06	+	1.73E-06	+	3.52E-06	+	6.27E-02	=	1.73E-06	+	1.73E-06	+
**F3**	1.73E-06	+	1.73E-06	+	1.73E-06	+	1.73E-06	+	1.73E-06	+	5.79E-05	+	1.73E-06	+	8.19E-05	+	1.73E-06	+	1.73E-06	-
**F4**	1.73E-06	+	1.73E-06	+	1.73E-06	+	1.73E-06	+	1.73E-06	+	1.73E-06	+	1.73E-06	+	6.98E-06	+	1.73E-06	+	1.73E-06	+
**F5**	1.73E-06	+	1.73E-06	+	1.73E-06	+	1.73E-06	+	1.73E-06	+	1.73E-06	+	1.73E-06	+	9.71E-05	+	1.73E-06	+	1.73E-06	+
**F6**	1.73E-06	+	1.73E-06	+	1.73E-06	+	1.73E-06	+	3.61E-03	-	1.73E-06	+	1.73E-06	+	1.73E-06	+	1.73E-06	+	1.73E-06	+
**F7**	1.73E-06	+	1.73E-06	+	1.73E-06	+	1.73E-06	+	1.73E-06	+	1.73E-06	+	1.73E-06	+	1.73E-06	+	1.73E-06	+	1.73E-06	+
**F8**	1.73E-06	+	1.73E-06	+	1.73E-06	+	1.73E-06	+	1.73E-06	+	1.73E-06	+	1.73E-06	+	1.92E-06	+	1.73E-06	+	1.73E-06	+
**F9**	2.13E-06	+	1.92E-06	+	8.47E-06	+	2.35E-06	+	4.53E-04	-	2.71E-01	=	2.54E-01	=	5.75E-06	-	1.73E-06	+	2.26E-03	-
**F10**	2.22E-04	+	1.92E-06	+	7.34E-01	=	2.60E-06	+	5.32E-03	+	2.13E-06	-	2.88E-06	-	1.73E-06	-	1.73E-06	+	2.88E-06	-
**F11**	1.73E-06	+	1.73E-06	+	1.73E-06	+	1.73E-06	+	1.73E-06	+	1.73E-06	+	2.60E-06	-	1.73E-06	-	1.73E-06	+	1.73E-06	-
**F12**	1.73E-06	+	1.73E-06	+	1.73E-06	+	1.73E-06	+	1.73E-06	+	1.73E-06	+	2.60E-06	+	1.66E-02	+	1.73E-06	+	1.73E-06	+
**F13**	1.73E-06	+	1.73E-06	+	1.73E-06	+	1.73E-06	+	2.35E-06	+	1.73E-06	+	1.04E-03	+	1.57E-02	+	1.73E-06	+	1.73E-06	+
**F14**	1.73E-06	+	1.73E-06	+	1.73E-06	+	1.73E-06	+	1.73E-06	+	1.73E-06	+	8.22E-02	=	8.94E-04	+	1.73E-06	+	7.19E-02	=
**F15**	1.73E-06	+	1.73E-06	+	1.73E-06	+	1.73E-06	+	1.73E-06	+	1.73E-06	+	2.29E-01	=	9.43E-01	=	1.73E-06	+	1.73E-06	+
**F16**	1.73E-06	+	1.73E-06	+	1.73E-06	+	1.73E-06	+	1.73E-06	+	1.73E-06	+	7.69E-06	+	7.73E-03	+	1.73E-06	+	2.35E-06	+
**F17**	1.73E-06	+	1.73E-06	+	1.73E-06	+	1.73E-06	+	1.73E-06	+	1.73E-06	+	9.84E-03	+	1.29E-03	+	1.73E-06	+	1.92E-06	+
**F18**	1.73E-06	+	1.73E-06	+	1.73E-06	+	1.73E-06	+	1.73E-06	+	1.73E-06	+	7.04E-01	=	7.81E-01	=	1.73E-06	+	4.49E-02	-
**F19**	1.73E-06	+	1.73E-06	+	1.73E-06	+	1.73E-06	+	1.73E-06	+	1.73E-06	+	5.72E-01	=	7.81E-01	=	1.73E-06	+	1.73E-06	+
**F20**	1.36E-05	+	1.92E-06	+	3.06E-04	+	1.73E-06	+	1.36E-05	+	3.85E-03	-	2.41E-04	-	8.94E-04	-	1.73E-06	+	2.37E-01	=
**F21**	1.73E-06	+	1.73E-06	+	1.73E-06	+	1.73E-06	+	1.73E-06	+	1.73E-06	+	1.73E-06	+	1.73E-06	+	1.73E-06	+	1.73E-06	+
**F22**	1.49E-05	+	2.35E-06	+	3.61E-03	+	5.75E-06	+	1.96E-03	+	4.49E-02	-	2.89E-01	=	1.71E-03	-	1.73E-06	+	1.71E-01	=
**F23**	1.73E-06	+	1.73E-06	+	1.73E-06	+	1.73E-06	+	1.73E-06	+	1.73E-06	+	1.73E-06	+	1.73E-06	+	1.73E-06	+	1.73E-06	+
**F24**	1.73E-06	+	1.73E-06	+	1.73E-06	+	1.73E-06	+	1.73E-06	+	1.73E-06	+	1.73E-06	+	1.73E-06	+	1.73E-06	+	1.73E-06	+
**F25**	1.73E-06	+	1.73E-06	+	1.73E-06	+	1.73E-06	+	1.73E-06	+	1.73E-06	+	1.73E-06	+	1.20E-03	+	1.73E-06	+	1.73E-06	+
**F26**	1.73E-06	+	1.73E-06	+	1.73E-06	+	1.73E-06	+	1.73E-06	+	1.73E-06	+	1.92E-06	+	1.73E-06	+	1.73E-06	+	1.73E-06	+
**F27**	1.73E-06	+	1.73E-06	+	1.73E-06	+	1.73E-06	+	1.73E-06	+	1.73E-06	+	1.73E-06	+	1.73E-06	+	1.73E-06	+	1.73E-06	+
**F28**	1.73E-06	+	1.73E-06	+	1.73E-06	+	1.73E-06	+	1.73E-06	+	1.73E-06	+	1.73E-06	+	1.73E-06	+	1.73E-06	+	1.73E-06	+
**F29**	1.73E-06	+	1.73E-06	+	1.73E-06	+	1.73E-06	+	1.73E-06	+	1.73E-06	+	7.69E-06	+	5.67E-03	+	1.73E-06	+	1.73E-06	+
**F30**	1.73E-06	+	1.73E-06	+	1.73E-06	+	1.73E-06	+	1.73E-06	+	1.73E-06	+	1.73E-06	+	1.36E-05	+	1.73E-06	+	1.73E-06	+

### Experiments on the latest and champion algorithms

To further evaluate the performance of the proposed BTOA algorithm, we conduct comparisons with three state-of-the-art baselines: the recently proposed Divine Religions Algorithm (DRA), and two well-established champion algorithms-JADE (Adaptive Differential Evolution) and L-SHADE. These algorithms represent strong competitors in the field of heuristic optimization and serve as suitable benchmarks to assess the competitiveness of BTOA.

The comparison is carried out on the classical CEC2005 benchmark and the more challenging CEC2017 benchmark under three different dimensional settings: 30, 50, and 100. Each function is independently run 30 times. Table 20 reports the number of times each algorithm achieves the best fitness on each benchmark, while Table 21 presents the results of the Friedman test in terms of average ranks and overall rankings. Detailed results including mean, variance, and Wilcoxon signed-rank test statistics are provided in the Appendix (SI Tables 1–8).

**Table 20:** Number of times each algorithm achieved the lowest fitness in each benchmark.

Benchmark	BTOA	DRA	JADE	LSHADE
CEC2005	**21**	8	1	8
CEC2017 dim=30	**18**	0	3	11
CEC2017 dim=50	**19**	0	4	7
CEC2017 dim=100	**17**	0	7	6

**Table 21:** Friedman test results for each benchmark (Mean of rank and Overall rank).

Test Function	Type	BTOA	DRA	JADE	LSHADE
CEC2005	Mean of rank	**1.413**	2.804	3.261	2.522
	Overall rank	**1**	3	4	2
CEC2017 dim=30	Mean of rank	**1.733**	4.000	2.433	1.833
	Overall rank	**1**	4	3	2
CEC2017 dim=50	Mean of rank	**1.600**	4.000	2.433	1.967
	Overall rank	**1**	4	3	2
CEC2017 dim=100	Mean of rank	**1.800**	4.000	2.300	1.900
	Overall rank	**1**	4	3	2

On the **CEC2005 benchmark**, BTOA achieves a dominant performance, obtaining the best fitness in 21 out of 23 functions and ranking first in the Friedman test. This result indicates that BTOA is highly effective on classical test problems. Although DRA is a recent algorithm, it fails to outperform BTOA proposed methods consistently, suggesting possible overfitting to modern benchmarks. L-SHADE performs reliably across the board and ranks second overall, whereas JADE exhibits relatively poor performance.

On the **CEC2017 benchmark with 30 dimensions**, the performance gap between BTOA and the other algorithms begins to narrow. BTOA still leads in the number of best performances (18 functions), but L-SHADE shows increased competitiveness, outperforming BTOA on several functions. This suggests that in more modern and diverse problem landscapes, algorithmic adaptability plays a greater role. The Wilcoxon test results confirm that while BTOA remains statistically superior in most cases, L-SHADE is a strong secondary contender.

In the **50-dimensional setting**, the difficulty of optimization increases significantly. BTOA still achieves the highest number of wins (19 out of 30), but the performance differences among the top three algorithms (BTOA, L-SHADE, and JADE) become less pronounced in several functions. This reflects the increasing challenge of maintaining performance consistency as the problem space grows. Nevertheless, BTOA retains the lowest average rank, confirming its advantage in overall robustness.

Finally, in the **100-dimensional benchmark**, the limitations of DRA and JADE become more evident. These algorithms fail to scale effectively, while BTOA and L-SHADE emerge as the only two methods capable of delivering stable performance. Although BTOA’s win count drops slightly to 17, it still holds the best overall rank, indicating good scalability.

## Application of BTOA to engineering design problems

To evaluate the practicability and superiority of BTOA, it was compared with several state-of-the-art algorithms (i.e., BWO, SCA, AOA, Jaya, DE, AO,TTAO, PSA, CPO, HLOA) on a series of engineering optimization problems. Specifically, BTOA’s performance was tested on UAV path planning and six classical engineering problems. These problems include the design of a corrugated bulkhead, a multiple disk clutch brake design, the Blending-Pooling-Separation problem, an industrial refrigeration system, Himmelblau’s function, and a cantilever beam. These optimization problems encompass both equality and inequality constraints, as follows:

$$\begin{aligned} \begin{array}{rl} \min & \quad f(x),\quad x\in \textbf{R}^n\\ \text {Subject to}& \quad g_i(x)\leqslant 0\\ & \quad h_j(x)=0\end{array} \end{aligned}$$

where, $g_i$ and $h_j$ are respectively inequality constraints and equality constraints. $R^n$ is a Euclidean n-spaces.One of the major hurdles in tackling intricate engineering issues is constraint handling. Several effective techniques exist for managing constraints^58^, including penalty functions, hybrid methods, separation of objective functions and constraints, and special operators. Penalty function-based techniques for handling constraints penalize solutions that deviate significantly from the feasible region. This approach converts constrained optimization problems into unconstrained ones, thereby enabling the handling of constraints. The proposed algorithm aims to find the optimal solution within the feasible region, thus minimizing the computational expense of engineering optimization.

Consistent with previous evaluations, all algorithms were configured with a population size of 100, 1000 iterations, and were run 30 times. Quantitative analysis and statistical tests were performed to ensure the robustness of the results. Next, we will conduct a comparative analysis of the algorithms in UAV path planning and six classical engineering problems.

### Applications in UAV path planning

In this study, we address the path planning issue using a cost function that integrates both optimal criteria and UAV constraints. The detailed description of the problem, including the mathematical formulation and constraints, is provided in the appendix.

#### Results on UAV path planning

**Fig. 13:**
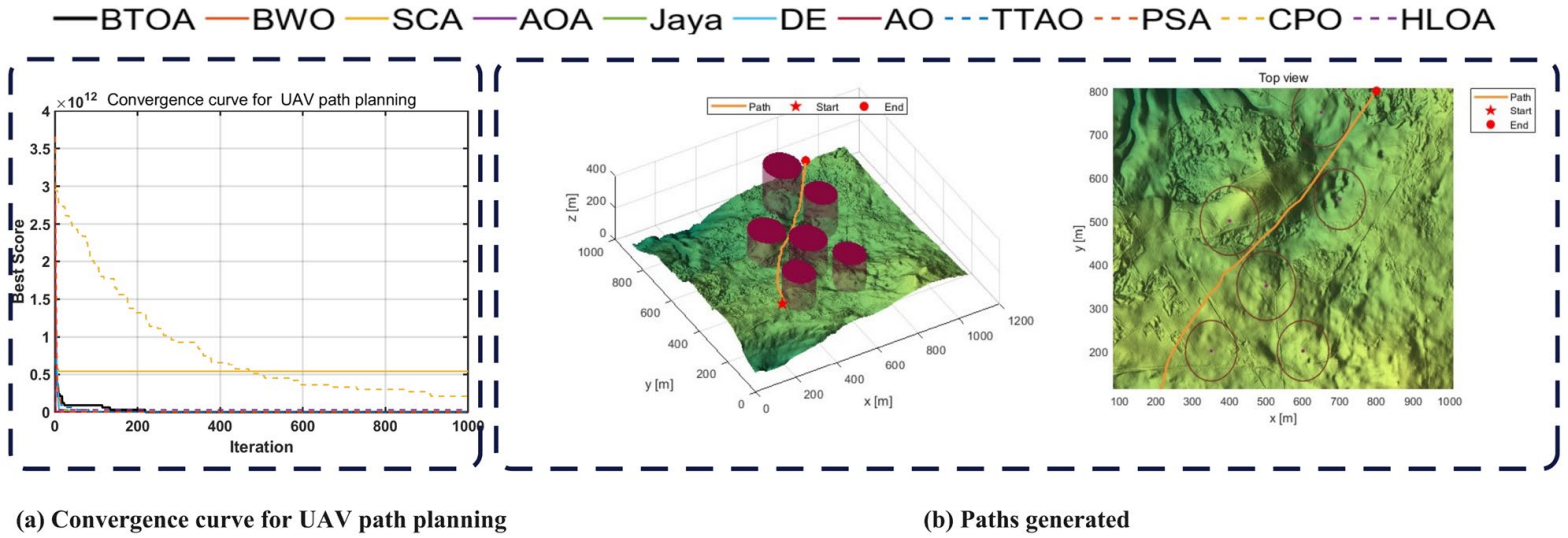
Convergence curves of BTOA in UAV path planning .

Figure 13 a presents the convergence curves of all algorithms for UAV path planning. While SCA and CPO converge slowly and get trapped in local optima, the other algorithms initially show rapid convergence. Although BTOA’s early convergence speed is not as fast as some algorithms, its iterative curve continues to decline after 200 iterations, quickly surpassing all other algorithms. Figure 13b illustrates the optimized path obtained by BTOA, which perfectly avoids all obstacles. Table 22 provides a clearer numerical representation, showing that BTOA’s mean result over 30 runs outperforms all comparison algorithms, despite its standard deviation not being the lowest, with only a small margin from the lowest value.

Table 23 presents the results of the Wilcoxon signed-rank test, comparing BTOA with each algorithm. The results indicate that BTOA is significantly superior to all comparison algorithms in UAV path planning, further demonstrating BTOA’s outstanding performance in this application.

**Table 22:** Performance comparison of various algorithms in UAV path planning.

Fun.	Index	BTOA	BWO	SCA	AOA	Jaya	DE	TTAO	PSA	CPO	AO	HLOA
UAV	AVE	**8.62E+03**	2.19E+04	5.40E+11	9.66E+03	1.39E+04	1.22E+04	9.26E+03	8.76E+03	2.10E+11	1.46E+04	3.00E+10
	STD	5.65E+02	2.39E+03	4.41E+11	**2.60E+02**	1.41E+03	3.40E+02	7.67E+02	5.17E+02	3.81E+11	1.22E+03	1.62E+11

**Table 23:** Wilcoxon signed-rank test on UAV path planning.

BTOA vs	BWO		SCA		AOA		Jaya		DE		TTAO		PSA		CPO	AO		HLOA	
	**p**	**win**	**p**	**win**	**p**	**win**	**p**	**win**	**p**	**win**	**p**	**win**	**p**	**win**	**p**	**p**	**win**	**p**	**win**
**UAV**	1.73E-06	+	1.73E-06	+	2.88E-06	+	1.73E-06	+	1.73E-06	+	1.73E-06	+	2.58E-03	+	1.85E-01	1.73E-06	+	2.35E-06	+

### Applications in six classical engineering problems

In this work, we compare six classic engineering optimization problems, including the Corrugated Bulkhead Design, Multiple Disk Clutch Brake Design, Blending-Pooling-Separation Problem, Industrial Refrigeration System Optimization, Himmelblau’s Function Problem, and Cantilever Beam Optimization Problem. The detailed descriptions of these problems, including their mathematical formulations, constraints, and optimization objectives, are provided in the Supplementary Information. Relevant illustrations are shown in SI Figures 1-4.

#### Results on six classical engineering problems

Table 24 presents the mean and standard deviation (STD) results of 30 runs of BTOA and 10 comparison algorithms on six classical engineering problems, where E1-E6 correspond to the six engineering problems previously described. As shown in the table, BTOA outperforms all comparison algorithms in five out of the six engineering problems. Although it does not achieve the best mean value in the Corrugated Bulkhead Design problem, it is very close to the best average value achieved by SCA. The STDs indicate that BTOA exhibits high stability in engineering problems, achieving the lowest standard deviation in four out of the six problems.

To investigate whether the comparison results are statistically significant, we used the Wilcoxon signed-rank test to compare the algorithms pairwise. Table 25 shows that out of 60 comparisons, BTOA significantly outperforms the comparison algorithms in 43 instances (71.67%), ties in 15 instances (25%), and underperforms in only 2 instances (3.33%).

Overall, BTOA demonstrates strong performance in engineering problems, effectively handling complex engineering optimization tasks with good stability.

**Table 24:** Performance comparison of various algorithms in six classical engineering problems.

Fun.	Index	BTOA	BWO	SCA	AOA	Jaya	DE	AO	TTAO	PSA	CPO	HLOA
E1	AVE	6.84E+00	6.76E+00	**6.38E+00**	7.76E+00	6.84E+00	6.84E+00	8.08E+00	6.92E+00	6.84E+00	6.84E+00	6.85E+00
	STD	**1.34E-10**	1.26E+00	2.54E+00	8.34E-01	7.63E-09	4.81E-05	5.43E-01	9.81E-01	8.25E-09	1.32E-07	5.28E-02
E2	AVE	**2.35E-01**	2.35E-01	2.36E-01	2.38E-01	**2.35E-01**	**2.35E-01**	3.16E-01	2.36E-01	**2.35E-01**	2.35E-01	2.35E-01
	STD	**5.55E-17**	1.87E-04	1.88E-04	6.53E-03	**5.55E-17**	**5.55E-17**	3.23E-02	2.73E-04	**5.55E-17**	1.04E-16	1.22E-16
E3	AVE	**5.37E+03**	3.40E+06	1.98E+06	2.10E+06	6.02E+04	1.58E+05	2.44E+07	1.32E+06	1.93E+04	7.86E+03	1.03E+06
	STD	**5.45E+03**	6.27E+05	1.82E+06	1.34E+06	4.76E+04	3.70E+04	8.23E+06	7.17E+05	1.63E+04	7.20E+03	1.22E+06
E4	AVE	**7.71E+00**	7.76E+00	8.43E+00	1.65E+02	1.28E+03	7.71E+00	2.22E+05	2.82E+03	**7.71E+00**	1.91E+03	3.22E+03
	STD	**1.44E-15**	3.81E-02	4.09E-01	2.02E+02	4.75E+03	6.08E-04	1.58E+05	6.87E+03	1.63E-15	5.71E+03	7.18E+03
E5	AVE	**-3.09E+04**	-3.09E+04	-3.08E+04	-3.07E+04	**-3.09E+04**	**-3.09E+04**	-3.04E+04	-3.09E+04	**-3.09E+04**	**-3.09E+04**	-3.09E+04
	STD	1.52E-11	2.19E-01	1.06E+01	6.03E+01	1.66E-11	1.55E-11	1.32E+02	2.78E-01	1.50E-11	2.04E-11	**1.30E-11**
E6	AVE	**1.34E+01**	1.34E+01	1.36E+01	1.75E+01	1.34E+01	1.34E+01	2.78E+01	1.34E+01	1.34E+01	1.34E+01	1.34E+01
	STD	7.62E-15	1.44E-02	1.14E-01	4.66E+00	7.43E-11	7.71E-08	5.70E+00	4.36E-03	8.83E-15	**6.66E-15**	1.43E-14

**Table 25:** Wilcoxon signed-rank test on six classical engineering problems.

BTOA vs	BWO		SCA		AOA		Jaya		DE		AO		TTAO		PSA		CPO		HLOA	
	p	win	p	win	p	win	p	win	p	win	p	win	p	win	p	win	p	win	p	win
**E1**	3.11E-05	-	1.48E-02	-	1.73E-06	+	1.73E-06	+	1.73E-06	+	1.73E-06	+	3.11E-05	+	1.73E-06	+	1.73E-06	+	6.89E-05	+
**E2**	1.73E-06	+	1.73E-06	+	1.61E-06	+	1.00E+00	=	1.00E+00	=	1.73E-06	+	1.73E-06	+	1.00E+00	=	1.00E+00	=	1.00E+00	=
**E3**	1.73E-06	+	1.73E-06	+	1.73E-06	+	3.88E-06	+	1.73E-06	+	1.73E-06	+	1.73E-06	+	6.98E-06	+	2.80E-01	=	1.73E-06	+
**E4**	1.73E-06	+	1.73E-06	+	1.73E-06	+	1.56E-01	=	1.73E-06	+	1.73E-06	+	1.73E-06	+	1.00E+00	=	5.52E-06	+	1.29E-06	+
**E5**	1.73E-06	+	1.73E-06	+	1.73E-06	+	1.00E+00	=	1.00E+00	=	1.73E-06	+	1.73E-06	+	1.00E+00	=	1.25E-01	=	2.50E-01	=
**E6**	1.73E-06	+	1.73E-06	+	1.73E-06	+	1.73E-06	+	1.73E-06	+	1.73E-06	+	1.73E-06	+	1.00E+00	=	6.25E-02	=	1.56E-02	+

## Discussion

In this work, we proposed the Basketball Team Optimization Algorithm (BTOA), a novel metaheuristic that translates key cooperative behaviors from basketball, such as high-intensity training, fast breaks, and dynamic positioning, into a structured optimization framework. The design of BTOA emphasizes both population diversity and adaptive control over the exploration-exploitation trade-off, which is a fundamental challenge in heuristic algorithm design.

One of BTOA’s major strengths lies in its hierarchical adaptation mechanism. Time-dependent control factors such as $\gamma (t)$ and *f* regulate the algorithm’s global search dynamics, while individual-level parameters like $\alpha _i$ and $\beta _i$ introduce variability based on fitness rank, enabling a diverse population to co-evolve effectively. The adaptivity factor $\eta$ allows each individual to dynamically choose between aggressive (fast break) or explorative (dynamic positioning) behaviors, enhancing search flexibility across complex landscapes.

Moreover, the proposed diagonal-guided positioning strategy provides a geometrically informed exploration mechanism, which is especially beneficial in high-dimensional spaces. Combined with randomized perturbations and a robust boundary control strategy, BTOA exhibits both search depth and breadth.

It is worth noting that the parameters used in BTOA are kept fixed across experiments to ensure fairness and reproducibility. Nevertheless, for specific problem domains, these parameters may be further adjusted to fine-tune the algorithm’s performance. Furthermore, BTOA is currently presented as a general-purpose baseline algorithm without embedded problem-specific enhancements. For domain-specific applications, additional hybrid or problem-oriented strategies could be integrated to further improve convergence speed or solution quality.

## Conclusions

This paper introduced the Basketball Team Optimization Algorithm (BTOA), a novel sports-inspired metaheuristic that models group dynamics and adaptive behaviors through metaphors drawn from basketball. The algorithm incorporates high-intensity learning mechanisms, diagonal-guided positioning strategies, and a probabilistic adaptivity control framework to maintain a fine-grained balance between exploration and exploitation throughout the search process.

Empirical validation on CEC2005 and CEC2017 benchmark suites, as well as several real-world engineering problems, demonstrated that BTOA delivers robust and competitive performance in both low and high-dimensional settings. Its modular design and stable convergence behavior make it a promising tool for continuous optimization tasks.

While BTOA has shown strong general performance, several opportunities remain for future work to extend its capabilities and applicability:

- *Extension to multi-objective optimization* Currently, BTOA is formulated for single-objective problems. Future work may extend its framework to support multi-objective optimization, enabling its application to Pareto-based decision-making scenarios.
- *Integration of problem-specific strategies* The current version of BTOA is a baseline general-purpose algorithm. It can be enhanced with domain-specific operators or hybrid mechanisms to better suit specific tasks such as discrete, constrained, or dynamic optimization.
- *Incorporation of learning mechanisms* Combining BTOA with machine learning components, such as reinforcement learning for strategy selection or surrogate modeling for fitness estimation, could further improve its adaptability and efficiency, particularly in expensive or real-time optimization environments.

## Supplementary Information

Supplementary Information.

## Data Availability

This study did not involve any human experiments or use of human data or samples. It focuses on the development of a heuristic optimization algorithm and does not utilize or generate any datasets. However, the code for the proposed algorithm will be made publicly available upon acceptance at the following repository: https://github.com/myqiaozhi/BTOA.
