# Supplementary material for: Basketball team optimization algorithm (BTOA): a novel sport-inspired meta-heuristic optimizer for engineering applications
Source: Sci Rep. 2025 Jul 1;15:21629. doi: 10.1038/s41598-025-05477-0 (PMC12214561; doi:10.1038/s41598-025-05477-0)
Supplement: Supplementary file 1 — Supplementary Information. [file 41598_2025_5477_MOESM1_ESM.pdf]

Appendix A

This appendix provides detailed numerical results to supplement the main experimental analysis presented in Section 4.4. It includes the mean and standard deviation of fitness values for each test function, as well as the Wilcoxon signed-rank test results comparing the proposed algorithm with each baseline. The results are reported in **SI Tables 1 to 8**, which cover all benchmark sets used in the comparative study.

SI Table 1. Performance Comparison of Additional Algorithms in CEC2005

Fun.	Index	BTOA	DRA	JADE	LSHADE	Fun.	Index	BTOA	DRA	JADE	LSHADE
F1	AVE	**0.00E+00**	**0.00E+00**	5.54E+03	1.62E+03	F13	AVE	**1.35E-32**	4.64E-02	2.47E+07	4.22E+06
	STD	**0.00E+00**	**0.00E+00**	7.76E+03	2.34E+02		STD	**5.47E-48**	6.91E-02	3.72E+07	2.06E+06
F2	AVE	**0.00E+00**	**0.00E+00**	2.15E+01	1.33E+01	F14	AVE	**9.98E-01**	1.02E+01	9.98E-01	**9.98E-01**
	STD	**0.00E+00**	**0.00E+00**	1.72E+01	1.58E+00		STD	**0.00E+00**	3.89E+00	8.43E-10	**0.00E+00**
F3	AVE	**0.00E+00**	**0.00E+00**	2.88E+04	5.70E+04	F15	AVE	**3.07E-04**	2.18E-03	4.09E-04	1.11E-03
	STD	**0.00E+00**	**0.00E+00**	3.59E+03	6.51E+03		STD	**2.17E-19**	6.99E-04	1.94E-04	3.59E-03
F4	AVE	**0.00E+00**	**0.00E+00**	4.58E+01	6.17E+01	F16	AVE	**-1.03E+00**	-4.90E-01	-1.03E+00	**-1.03E+00**
	STD	**0.00E+00**	**0.00E+00**	7.26E+00	5.40E+00		STD	**6.66E-16**	3.48E-01	1.82E-05	**6.66E-16**
F5	AVE	2.37E+01	**7.12E+00**	7.13E+06	1.45E+06	F17	AVE	**3.98E-01**	7.18E-01	3.98E-01	**3.98E-01**
	STD	**1.74E-01**	9.77E+00	1.14E+07	4.39E+05		STD	**0.00E+00**	1.62E-01	1.10E-05	**0.00E+00**
F6	AVE	**0.00E+00**	4.70E-03	1.67E+00	**0.00E+00**	F18	AVE	**3.00E+00**	2.02E+01	3.00E+00	3.00E+00
	STD	**0.00E+00**	6.96E-03	4.13E+00	**0.00E+00**		STD	**1.30E-15**	1.08E+01	4.64E-04	1.33E-15
F7	AVE	**7.71E-05**	1.27E-03	3.75E+00	1.11E+00	F19	AVE	**-3.86E+00**	-3.61E+00	-3.86E+00	**-3.86E+00**
	STD	**7.65E-05**	1.03E-03	5.11E+00	2.60E-01		STD	**2.66E-15**	1.81E-01	3.03E-05	**2.66E-15**
F8	AVE	**-1.26E+04**	-2.69E+03	-8.68E+03	-1.12E+04	F20	AVE	-3.27E+00	-2.09E+00	**-3.32E+00**	-3.22E+00
	STD	**1.82E-12**	3.91E+02	1.28E+03	2.77E+02		STD	5.93E-02	4.33E-01	**9.59E-03**	4.05E-02
F9	AVE	**0.00E+00**	**0.00E+00**	1.43E+02	1.17E+02	F21	AVE	**-1.02E+01**	-9.25E+00	-1.01E+01	**-1.02E+01**
	STD	**0.00E+00**	**0.00E+00**	5.25E+01	9.92E+00		STD	**7.00E-15**	1.07E+00	1.76E-01	**7.00E-15**
F10	AVE	**8.88E-16**	**8.88E-16**	1.25E+01	1.38E+01	F22	AVE	**-1.04E+01**	-9.81E+00	-1.03E+01	**-1.04E+01**
	STD	**0.00E+00**	**0.00E+00**	3.93E+00	2.66E+00		STD	1.52E-15	8.10E-01	1.66E-01	**1.45E-15**
F11	AVE	**0.00E+00**	**0.00E+00**	2.79E+01	1.49E+01	F23	AVE	**-1.05E+01**	-9.95E+00	-1.04E+01	**-1.05E+01**
	STD	**0.00E+00**	**0.00E+00**	4.45E+01	2.34E+00		STD	**1.75E-15**	8.85E-01	3.27E-01	1.78E-15
F12	AVE	**1.57E-32**	2.02E-03	4.13E+06	9.64E+05						
	STD	**5.47E-48**	3.13E-03	8.90E+06	6.68E+05						

SI Table 2. Wilcoxon Signed-Rank Test Comparing Additional Algorithms in CEC2005

BTOA vs	DRA		JADE		LSHADE		BTOA vs	DRA		JADE		LSHADE	
	p	win	p	win	p	win		p	win	p	win	p	win
**F1**	1.00E+00	=	1.73E-06	+	1.73E-06	+	**F13**	1.73E-06	+	1.73E-06	+	1.73E-06	+
**F2**	1.00E+00	=	1.73E-06	+	1.73E-06	+	**F14**	1.73E-06	+	6.25E-02	=	1.00E+00	=
**F3**	1.00E+00	=	1.73E-06	+	1.73E-06	+	**F15**	1.73E-06	+	1.73E-06	+	1.73E-06	+
**F4**	1.00E+00	=	1.73E-06	+	1.73E-06	+	**F16**	1.73E-06	+	2.50E-01	=	1.00E+00	=
**F5**	5.22E-06	-	1.73E-06	+	1.73E-06	+	**F17**	1.73E-06	+	1.25E-01	=	1.00E+00	=
**F6**	1.73E-06	+	3.91E-03	+	1.00E+00	=	**F18**	1.73E-06	+	1.45E-07	+	4.32E-08	+
**F7**	1.92E-06	+	1.73E-06	+	1.73E-06	+	**F19**	1.73E-06	+	6.25E-02	=	1.00E+00	=
**F8**	1.73E-06	+	1.73E-06	+	1.73E-06	+	**F20**	1.73E-06	+	5.02E-04	-	1.81E-03	+
**F9**	1.00E+00	=	1.73E-06	+	1.73E-06	+	**F21**	1.73E-06	+	1.56E-02	+	1.00E+00	=
**F10**	1.00E+00	=	1.73E-06	+	1.73E-06	+	**F22**	1.73E-06	+	6.25E-02	=	1.00E+00	=
**F11**	1.00E+00	=	1.73E-06	+	1.73E-06	+	**F23**	1.73E-06	+	3.91E-03	+	1.00E+00	=
**F12**	1.73E-06	+	1.73E-06	+	1.73E-06	+							

SI Table 3. Performance Comparison of Additional Algorithms in CEC2017 (Dim = 30)

Fun.	Index	BTOA	DRA	JADE	LSHADE	Fun.	Index	BTOA	DRA	JADE	LSHADE
F1	AVE	4.52E+03	8.12E+10	1.00E+02	**1.00E+02**	F16	AVE	**2.15E+03**	8.05E+03	2.74E+03	2.52E+03
	STD	4.27E+03	2.50E+09	1.45E-02	**6.56E-03**		STD	2.38E+02	1.78E+03	**1.28E+02**	1.59E+02
F2	AVE	1.17E+13	1.50E+52	1.36E+10	**4.60E+02**	F17	AVE	**1.86E+03**	1.20E+04	1.92E+03	1.91E+03
	STD	5.10E+13	7.12E+52	5.40E+10	**1.36E+03**		STD	7.83E+01	1.17E+04	4.51E+01	**4.16E+01**
F3	AVE	**1.26E+04**	1.19E+05	2.78E+04	3.64E+04	F18	AVE	1.50E+05	5.74E+08	2.63E+05	**1.46E+05**
	STD	**3.99E+03**	6.67E+04	4.36E+03	5.34E+03		STD	9.39E+04	3.11E+08	8.82E+04	**6.44E+04**
F4	AVE	4.91E+02	2.99E+04	4.98E+02	**4.84E+02**	F19	AVE	6.72E+03	4.24E+09	2.05E+03	**1.99E+03**
	STD	2.25E+01	9.94E+02	1.53E+01	**9.61E+00**		STD	6.82E+03	9.98E+08	3.82E+01	**1.79E+01**
F5	AVE	**5.57E+02**	1.04E+03	6.57E+02	6.35E+02	F20	AVE	**2.22E+03**	3.77E+03	2.33E+03	2.26E+03
	STD	1.53E+01	1.07E+01	**9.16E+00**	9.57E+00		STD	1.04E+02	2.00E+02	5.96E+01	**5.76E+01**
F6	AVE	6.00E+02	7.23E+02	6.00E+02	**6.00E+02**	F21	AVE	**2.35E+03**	2.91E+03	2.45E+03	2.43E+03
	STD	2.19E-02	7.56E+00	3.42E-08	**6.27E-09**		STD	1.21E+01	4.15E+01	1.13E+01	**1.10E+01**
F7	AVE	**7.92E+02**	1.59E+03	8.90E+02	8.75E+02	F22	AVE	2.30E+03	1.18E+04	**2.30E+03**	**2.30E+03**
	STD	2.11E+01	**2.73E+00**	1.07E+01	8.06E+00		STD	1.66E+00	5.78E+02	**2.99E-13**	3.42E-13
F8	AVE	**8.65E+02**	1.28E+03	9.58E+02	9.35E+02	F23	AVE	**2.70E+03**	3.83E+03	2.79E+03	2.78E+03
	STD	2.34E+01	**3.31E+00**	7.90E+00	1.02E+01		STD	1.26E+01	1.61E+02	1.42E+01	**1.16E+01**
F9	AVE	9.47E+02	1.76E+04	**9.00E+02**	**9.00E+02**	F24	AVE	**2.87E+03**	4.20E+03	2.97E+03	2.95E+03
	STD	1.51E+02	1.50E+02	**2.08E-14**	4.15E-14		STD	1.85E+01	2.31E+02	**1.13E+01**	1.49E+01
F10	AVE	**7.02E+03**	1.06E+04	7.89E+03	7.54E+03	F25	AVE	**2.89E+03**	8.32E+03	2.89E+03	2.89E+03
	STD	1.51E+03	3.53E+02	3.20E+02	**2.44E+02**		STD	2.13E+00	3.79E+02	**1.83E-01**	3.25E-01
F11	AVE	**1.16E+03**	4.46E+04	1.17E+03	1.17E+03	F26	AVE	**3.89E+03**	1.49E+04	4.90E+03	4.80E+03
	STD	2.90E+01	3.30E+04	**2.67E+01**	2.90E+01		STD	5.44E+02	1.07E+03	1.26E+02	**1.12E+02**
F12	AVE	**1.18E+05**	2.12E+10	2.00E+05	1.36E+05	F27	AVE	3.21E+03	5.01E+03	**3.20E+03**	3.21E+03
	STD	1.18E+05	4.98E+09	1.45E+05	**1.09E+05**		STD	1.05E+01	3.34E+02	7.23E+00	**4.64E+00**
F13	AVE	**9.28E+03**	2.46E+10	1.80E+04	1.87E+04	F28	AVE	3.21E+03	9.62E+03	3.20E+03	**3.18E+03**
	STD	8.64E+03	7.98E+09	**6.25E+03**	7.93E+03		STD	2.40E+01	2.36E+02	**1.26E+01**	5.67E+01
F14	AVE	6.97E+03	3.99E+07	1.58E+03	**1.52E+03**	F29	AVE	**3.55E+03**	2.07E+04	3.67E+03	3.68E+03
	STD	1.08E+04	2.67E+07	1.60E+01	**1.45E+01**		STD	1.49E+02	3.35E+04	**6.19E+01**	8.39E+01
F15	AVE	8.50E+03	4.17E+09	1.94E+03	**1.80E+03**	F30	AVE	**1.28E+04**	3.25E+09	2.55E+04	1.69E+04
	STD	6.24E+03	1.31E+09	1.02E+02	**6.58E+01**		STD	**3.70E+03**	1.42E+09	9.35E+03	6.41E+03

SI Table 4. Wilcoxon Signed-Rank Test Comparing Additional Algorithms in CEC2017 (Dim = 30)

BTOA vs	DRA		JADE		LSHADE		BTOA vs	DRA		JADE		LSHADE	
	p	win	p	win	p	win		p	win	p	win	p	win
**F1**	1.73E-06	+	1.73E-06	-	1.73E-06	-	**F16**	1.73E-06	+	1.73E-06	+	1.13E-05	+
**F2**	1.73E-06	+	1.60E-04	-	1.73E-06	-	**F17**	1.73E-06	+	3.88E-04	+	9.63E-04	+
**F3**	1.73E-06	+	1.92E-06	+	1.73E-06	+	**F18**	1.73E-06	+	1.74E-04	+	6.88E-01	=
**F4**	1.73E-06	+	1.92E-01	=	1.66E-02	-	**F19**	1.73E-06	+	1.73E-06	-	1.73E-06	-
**F5**	1.73E-06	+	1.73E-06	+	1.73E-06	+	**F20**	1.73E-06	+	3.06E-04	+	1.65E-01	=
**F6**	1.73E-06	+	1.73E-06	-	1.73E-06	-	**F21**	1.73E-06	+	1.73E-06	+	1.73E-06	+
**F7**	1.73E-06	+	1.73E-06	+	1.73E-06	+	**F22**	1.73E-06	+	3.12E-02	-	3.12E-02	-
**F8**	1.73E-06	+	1.73E-06	+	1.92E-06	+	**F23**	1.73E-06	+	1.73E-06	+	1.73E-06	+
**F9**	1.73E-06	+	2.56E-06	-	2.56E-06	-	**F24**	1.73E-06	+	1.73E-06	+	1.73E-06	+
**F10**	1.73E-06	+	2.30E-02	+	5.30E-01	=	**F25**	1.73E-06	+	1.32E-02	+	2.58E-03	+
**F11**	1.73E-06	+	1.78E-01	=	1.85E-01	=	**F26**	1.73E-06	+	1.73E-06	+	1.73E-06	+
**F12**	1.73E-06	+	5.67E-03	+	4.41E-01	=	**F27**	1.73E-06	+	6.42E-03	-	7.73E-03	-
**F13**	1.73E-06	+	1.38E-03	+	9.63E-04	+	**F28**	1.73E-06	+	8.97E-02	=	5.67E-03	-
**F14**	1.73E-06	+	1.73E-06	-	1.73E-06	-	**F29**	1.73E-06	+	1.96E-03	+	2.41E-03	+
**F15**	1.73E-06	+	1.92E-06	-	1.92E-06	-	**F30**	1.73E-06	+	4.73E-06	+	8.94E-04	+

SI Table 5. Performance Comparison of Additional Algorithms in CEC2017 (Dim = 50)

Fun.	Index	BTOA	DRA	JADE	LSHADE	Fun.	Index	BTOA	DRA	JADE	LSHADE
F1	AVE	3.67E+03	1.33E+11	**3.06E+03**	4.04E+03	F16	AVE	**2.95E+03**	1.30E+04	4.14E+03	4.01E+03
	STD	**4.03E+03**	2.82E+09	4.04E+03	4.42E+03		STD	3.45E+02	1.98E+03	3.01E+02	**2.13E+02**
F2	AVE	1.64E+29	1.40E+87	8.58E+29	**2.00E+12**	F17	AVE	**2.66E+03**	1.44E+05	3.35E+03	3.17E+03
	STD	5.08E+29	1.69E+87	4.60E+30	**5.08E+12**		STD	2.69E+02	4.90E+04	1.78E+02	**1.51E+02**
F3	AVE	**7.80E+04**	9.96E+09	1.14E+05	1.43E+05	F18	AVE	**7.28E+05**	9.86E+08	2.12E+06	1.15E+06
	STD	**1.14E+04**	2.64E+10	1.64E+04	2.10E+04		STD	4.62E+05	4.82E+08	6.21E+05	**2.55E+05**
F4	AVE	5.30E+02	5.47E+04	5.52E+02	**5.00E+02**	F19	AVE	1.86E+04	1.02E+10	1.33E+04	**1.22E+04**
	STD	5.19E+01	3.63E+03	**4.17E+01**	4.78E+01		STD	1.20E+04	2.38E+09	**5.36E+03**	5.36E+03
F5	AVE	**6.68E+02**	1.36E+03	8.43E+02	8.09E+02	F20	AVE	**2.91E+03**	5.28E+03	3.53E+03	3.39E+03
	STD	4.91E+01	**9.19E+00**	1.28E+01	1.56E+01		STD	3.72E+02	2.27E+02	2.19E+02	**1.72E+02**
F6	AVE	6.01E+02	7.35E+02	6.00E+02	**6.00E+02**	F21	AVE	**2.41E+03**	3.49E+03	2.64E+03	2.60E+03
	STD	4.74E-01	2.68E+00	**3.65E-07**	4.68E-07		STD	2.74E+01	7.27E+01	**1.39E+01**	1.42E+01
F7	AVE	**8.98E+02**	2.19E+03	1.09E+03	1.07E+03	F22	AVE	1.09E+04	1.94E+04	**8.95E+03**	1.47E+04
	STD	4.02E+01	2.26E+01	**1.28E+01**	1.83E+01		STD	4.89E+03	**6.34E+02**	6.66E+03	2.44E+03
F8	AVE	**9.46E+02**	1.70E+03	1.14E+03	1.10E+03	F23	AVE	**2.84E+03**	5.05E+03	3.06E+03	3.03E+03
	STD	3.69E+01	2.50E+01	1.55E+01	**1.43E+01**		STD	2.97E+01	2.66E+02	**1.10E+01**	1.90E+01
F9	AVE	2.05E+03	6.47E+04	**9.00E+02**	9.00E+02	F24	AVE	**3.01E+03**	5.52E+03	3.24E+03	3.20E+03
	STD	2.05E+03	1.16E+03	**1.41E-01**	4.81E-01		STD	3.09E+01	3.10E+02	**1.34E+01**	1.71E+01
F10	AVE	**1.22E+04**	1.73E+04	1.40E+04	1.38E+04	F25	AVE	3.06E+03	1.93E+04	3.06E+03	**3.03E+03**
	STD	2.31E+03	**3.93E+02**	4.04E+02	4.97E+02		STD	3.52E+01	5.68E+02	**2.23E+01**	3.99E+01
F11	AVE	**1.27E+03**	1.24E+05	1.28E+03	1.28E+03	F26	AVE	**3.41E+03**	2.00E+04	6.80E+03	6.52E+03
	STD	4.31E+01	1.24E+05	**1.95E+01**	2.67E+01		STD	1.07E+03	3.08E+02	1.77E+02	**1.52E+02**
F12	AVE	2.20E+06	1.24E+11	1.11E+06	**7.23E+05**	F27	AVE	3.29E+03	8.28E+03	**3.24E+03**	3.28E+03
	STD	1.58E+06	1.93E+10	4.05E+05	**3.24E+05**		STD	4.12E+01	6.85E+02	**1.62E+01**	4.07E+01
F13	AVE	**5.78E+03**	7.14E+10	1.28E+04	2.86E+04	F28	AVE	**3.29E+03**	1.92E+04	3.31E+03	3.30E+03
	STD	6.98E+03	1.61E+10	**6.20E+03**	2.04E+04		STD	2.50E+01	1.36E+03	2.94E+01	**1.67E+01**
F14	AVE	7.78E+04	2.75E+08	6.37E+04	**3.22E+04**	F29	AVE	**3.83E+03**	9.41E+05	4.32E+03	4.29E+03
	STD	4.22E+04	1.40E+08	2.03E+04	**1.37E+04**		STD	2.30E+02	1.03E+06	2.37E+02	**1.99E+02**
F15	AVE	**9.90E+03**	2.17E+10	1.05E+04	1.13E+04	F30	AVE	**9.92E+05**	1.60E+10	1.66E+06	1.30E+06
	STD	7.44E+03	2.95E+09	**3.46E+03**	4.50E+03		STD	**2.64E+05**	4.47E+09	6.32E+05	4.05E+05

SI Table 6. Wilcoxon Signed-Rank Test Comparing Additional Algorithms in CEC2017 (Dim = 50)

BTOA vs	DRA		JADE		LSHADE		BTOA vs	DRA		JADE		LSHADE	
	p	win	p	win	p	win		p	win	p	win	p	win
**F1**	1.73E-06	+	9.10E-01	=	6.00E-01	=	**F16**	1.73E-06	+	1.73E-06	+	1.73E-06	+
**F2**	1.73E-06	+	1.20E-03	+	1.73E-06	-	**F17**	1.73E-06	+	1.73E-06	+	3.52E-06	+
**F3**	1.73E-06	+	3.18E-06	+	1.73E-06	+	**F18**	1.73E-06	+	1.92E-06	+	3.32E-04	+
**F4**	1.73E-06	+	1.06E-01	=	3.00E-02	-	**F19**	1.73E-06	+	5.19E-02	=	8.73E-03	-
**F5**	1.73E-06	+	1.73E-06	+	1.92E-06	+	**F20**	1.73E-06	+	1.13E-05	+	1.80E-05	+
**F6**	1.73E-06	+	1.73E-06	-	1.73E-06	-	**F21**	1.73E-06	+	1.73E-06	+	1.73E-06	+
**F7**	1.73E-06	+	1.73E-06	+	1.73E-06	+	**F22**	1.73E-06	+	3.18E-01	=	3.06E-04	+
**F8**	1.73E-06	+	1.73E-06	+	1.73E-06	+	**F23**	1.73E-06	+	1.73E-06	+	1.73E-06	+
**F9**	1.73E-06	+	1.73E-06	-	1.73E-06	-	**F24**	1.73E-06	+	1.73E-06	+	1.73E-06	+
**F10**	1.73E-06	+	8.31E-04	+	7.73E-03	+	**F25**	1.73E-06	+	6.44E-01	=	1.32E-02	-
**F11**	1.73E-06	+	2.99E-01	=	2.37E-01	=	**F26**	1.73E-06	+	1.92E-06	+	1.92E-06	+
**F12**	1.73E-06	+	2.11E-03	-	2.84E-05	-	**F27**	1.73E-06	+	1.24E-05	-	9.26E-01	=
**F13**	1.73E-06	+	1.06E-04	+	1.13E-05	+	**F28**	1.73E-06	+	7.27E-03	+	6.00E-01	=
**F14**	1.73E-06	+	1.71E-01	=	4.07E-05	-	**F29**	1.73E-06	+	3.18E-06	+	5.22E-06	+
**F15**	1.73E-06	+	4.17E-01	=	9.78E-02	=	**F30**	1.73E-06	+	4.07E-05	+	2.26E-03	+

SI Table 7. Performance Comparison of Additional Algorithms in CEC2017 (Dim = 100)

Fun.	Index	BTOA	DRA	JADE	LSHADE	Fun.	Index	BTOA	DRA	JADE	LSHADE
F1	AVE	2.62E+05	2.95E+11	**3.97E+03**	7.73E+03	F16	AVE	**5.48E+03**	3.53E+04	9.27E+03	8.85E+03
	STD	4.00E+05	5.75E+09	**3.85E+03**	7.47E+03		STD	8.83E+02	3.84E+03	**3.74E+02**	3.91E+02
F2	AVE	4.52E+86	4.04E+189	2.60E+55	**1.52E+54**	F17	AVE	**4.79E+03**	6.63E+07	6.53E+03	6.38E+03
	STD	2.43E+87	**6.55E+04**	1.19E+56	7.93E+54		STD	6.78E+02	3.61E+07	**2.92E+02**	3.08E+02
F3	AVE	**2.88E+05**	4.66E+10	3.86E+05	4.62E+05	F18	AVE	**1.60E+06**	1.18E+09	1.00E+07	4.26E+06
	STD	**2.11E+04**	1.95E+11	3.91E+04	4.15E+04		STD	**9.08E+05**	2.29E+08	2.21E+06	1.10E+06
F4	AVE	7.41E+02	1.55E+05	7.00E+02	**6.62E+02**	F19	AVE	5.37E+03	3.81E+10	4.25E+03	**4.19E+03**
	STD	4.89E+01	7.60E+03	3.80E+01	**3.40E+01**		STD	3.15E+03	4.57E+09	**1.99E+03**	3.11E+03
F5	AVE	**1.05E+03**	2.35E+03	1.37E+03	1.30E+03	F20	AVE	**6.53E+03**	9.57E+03	7.08E+03	6.85E+03
	STD	1.32E+02	2.52E+01	**2.48E+01**	2.73E+01		STD	8.17E+02	3.29E+02	2.63E+02	**2.43E+02**
F6	AVE	6.11E+02	7.28E+02	**6.00E+02**	6.00E+02	F21	AVE	**2.67E+03**	5.40E+03	3.18E+03	3.12E+03
	STD	4.76E+00	1.13E+00	**9.94E-03**	1.02E-02		STD	5.80E+01	1.60E+02	3.19E+01	**2.97E+01**
F7	AVE	**1.34E+03**	4.23E+03	1.65E+03	1.61E+03	F22	AVE	**2.39E+04**	3.87E+04	3.35E+04	3.28E+04
	STD	9.84E+01	**1.63E+01**	2.37E+01	2.27E+01		STD	8.26E+03	6.81E+02	**5.73E+02**	6.10E+02
F8	AVE	**1.22E+03**	2.83E+03	1.66E+03	1.60E+03	F23	AVE	**3.14E+03**	8.04E+03	3.67E+03	3.60E+03
	STD	5.08E+01	**1.80E+01**	2.82E+01	1.80E+01		STD	3.92E+01	4.69E+02	3.92E+01	**2.87E+01**
F9	AVE	3.99E+04	1.06E+05	**9.05E+02**	9.12E+02	F24	AVE	**3.63E+03**	1.37E+04	4.09E+03	4.06E+03
	STD	1.52E+04	1.03E+03	**3.23E+00**	7.53E+00		STD	8.63E+01	8.09E+02	**3.49E+01**	3.95E+01
F10	AVE	**2.73E+04**	3.59E+04	3.14E+04	3.08E+04	F25	AVE	3.38E+03	3.50E+04	3.36E+03	**3.30E+03**
	STD	5.24E+03	8.73E+02	**4.86E+02**	6.08E+02		STD	5.01E+01	7.45E+02	**4.40E+01**	4.45E+01
F11	AVE	**1.41E+04**	6.18E+07	2.40E+04	2.95E+04	F26	AVE	**9.03E+03**	6.37E+04	1.39E+04	1.34E+04
	STD	**3.82E+03**	2.19E+08	4.35E+03	5.72E+03		STD	1.57E+03	6.13E+02	**2.92E+02**	3.64E+02
F12	AVE	1.24E+07	2.55E+11	**2.81E+06**	4.06E+06	F27	AVE	3.51E+03	1.63E+04	**3.37E+03**	3.40E+03
	STD	6.07E+06	9.91E+09	**1.10E+06**	2.15E+06		STD	6.41E+01	1.82E+03	**2.60E+01**	3.26E+01
F13	AVE	8.06E+03	6.41E+10	**5.29E+03**	5.94E+03	F28	AVE	3.51E+03	4.03E+04	3.51E+03	**3.42E+03**
	STD	6.58E+03	1.90E+09	**2.53E+03**	3.94E+03		STD	**3.11E+01**	7.29E+02	3.75E+01	3.43E+01
F14	AVE	**6.92E+05**	7.49E+08	4.65E+06	2.09E+06	F29	AVE	**6.59E+03**	6.43E+06	8.42E+03	8.24E+03
	STD	**3.28E+05**	2.45E+08	1.12E+06	7.30E+05		STD	6.54E+02	2.48E+06	3.92E+02	**3.30E+02**
F15	AVE	4.09E+03	3.77E+10	**3.97E+03**	5.59E+03	F30	AVE	1.64E+04	5.89E+10	1.48E+04	**1.07E+04**
	STD	2.36E+03	4.50E+09	**1.46E+03**	4.08E+03		STD	6.61E+03	3.05E+09	3.56E+03	**2.99E+03**

SI Table 8. Wilcoxon Signed-Rank Test Comparing Additional Algorithms in CEC2017 (Dim = 100)

BTOA vs	DRA		JADE		LSHADE		BTOA vs	DRA		JADE		LSHADE	
	p	win	p	win	p	win		p	win	p	win	p	win
**F1**	1.73E-06	+	1.73E-06	-	1.73E-06	-	**F16**	1.73E-06	+	1.73E-06	+	1.73E-06	+
**F2**	1.73E-06	+	1.73E-06	-	1.73E-06	-	**F17**	1.73E-06	+	1.73E-06	+	1.92E-06	+
**F3**	1.73E-06	+	1.73E-06	+	1.73E-06	+	**F18**	1.73E-06	+	1.73E-06	+	1.92E-06	+
**F4**	1.73E-06	+	2.58E-03	-	7.69E-06	-	**F19**	1.73E-06	+	2.37E-01	=	1.65E-01	=
**F5**	1.73E-06	+	1.73E-06	+	3.88E-06	+	**F20**	1.73E-06	+	1.13E-05	+	1.80E-05	+
**F6**	1.73E-06	+	1.73E-06	-	1.73E-06	-	**F21**	1.73E-06	+	1.73E-06	+	1.73E-06	+
**F7**	1.73E-06	+	1.73E-06	+	1.73E-06	+	**F22**	1.73E-06	+	3.72E-05	+	6.32E-05	+
**F8**	1.73E-06	+	1.73E-06	+	1.73E-06	+	**F23**	1.73E-06	+	1.73E-06	+	1.73E-06	+
**F9**	1.73E-06	+	1.73E-06	-	1.73E-06	-	**F24**	1.73E-06	+	1.73E-06	+	1.73E-06	+
**F10**	1.73E-06	+	5.79E-05	+	7.27E-03	+	**F25**	1.73E-06	+	2.21E-01	=	7.51E-05	-
**F11**	1.73E-06	+	5.22E-06	+	1.73E-06	+	**F26**	1.73E-06	+	1.92E-06	+	1.92E-06	+
**F12**	1.73E-06	+	1.92E-06	-	9.32E-06	-	**F27**	1.73E-06	+	1.73E-06	-	3.52E-06	-
**F13**	1.73E-06	+	5.19E-02	=	1.65E-01	=	**F28**	1.73E-06	+	2.99E-01	=	2.35E-06	-
**F14**	1.73E-06	+	1.73E-06	+	1.73E-06	+	**F29**	1.73E-06	+	1.73E-06	+	2.35E-06	+
**F15**	1.73E-06	+	9.92E-01	=	1.53E-01	=	**F30**	1.73E-06	+	7.34E-01	=	1.06E-04	-

Appendix B

Path Optimality

To ensure the efficient operation of UAVs, the planned route must meet specific optimality criteria based on the intended application. For tasks such as aerial photography, mapping, and surface inspection, our objective is to minimize the total path length. The UAV, which is directed by a ground control station (GCS), follows a flight path X_i, represented as a sequence of n waypoints. Each waypoint serves as a node in the search process.

Map with coordinates $P_{ij} = (x_{ij}, y_{ij}, z_{ij})$.By denoting the Euclidean distance between two nodes as $\left\|\overrightarrow{P_{ij}P_{i,j+1}}\right\|$, the cost F_1 associated to the path length can be compuited as:

$$F_1(X_i) = \sum_{j=1}^{n-1} \left\|\overrightarrow{P_{ij}P_{i,j+1}}\right\|.$$

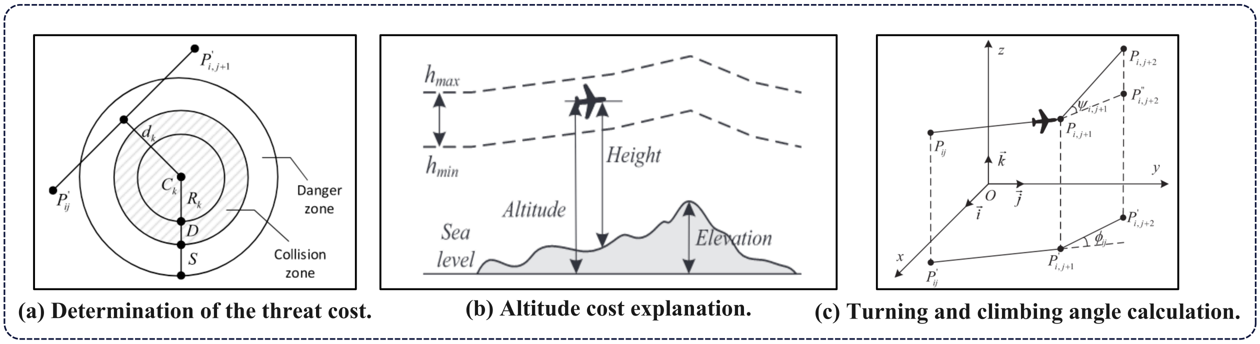

SI Figure 1: Description of UAV path planning

Safety and Feasibility Constraints

In addition to ensuring optimality, it is crucial for the planned path to guarantee the UAV operates safely by navigating around threats typically caused by obstacles within the operational space. Denote K as the set of all potential threats, each represented as a cylinder. The projection of each threat cylinder has a center coordinate C_k and a radius R_k, as illustrated in **SI Figure 1 (a)**. For any given segment of the path $\left|\overrightarrow{P_{ij}P_{i,j+1}}\right|$, the threat cost is related to its distance, d_k, from C_k. Taking into account the UAV's diameter, D, and the safety distance, S, from the collision zone, the threat cost F_2 is calculated across the waypoints P_{ij} for the obstacle set K as follows:

$$F_2(X_i) = \sum_{j=1}^{n-1}\sum_{k=1}^{K} T_k(\overrightarrow{P_{ij}P_{i,j+1}}),$$

$$T_k(\overrightarrow{P_{ij}P_{i,j+1}}) = \begin{cases} 0, & \text{if } d_k > S + D + R_k \\ (S + D + R_k) - d_k, & \text{if } D + R_k < d_k \leq S + D + R_k \\ \infty, & \text{if } d_k \leq D + R_k. \end{cases}$$

In the context of the flight height with respect to the ground, denoted as h_{ij} and depicted in **SI Figure 1 (b)**, H_{ij} is designed to maintain the average altitude while penalizing values that deviate significantly. The altitude cost is determined by summing H_{ij} across all waypoints:

$$F_3(X_i) = \sum_{j=1}^{n} H_{ij}.$$

The smoothness cost assesses the rates of turning and climbing, which are crucial for creating feasible paths. **SI Figure 1 (c)** illustrates that the turning angle, ϕ_{ij} , represents the angle between two successive path segments, $\overrightarrow{P'_{ij}P'_{i,j+1}}$ and $\overrightarrow{P'_{i,j+1}P'_{i,j+2}}$, projected onto the horizontal plane Oxy. Let $\overrightarrow{k}$ be the unit vector along the z axis. The projected vector can then be calculated as follows:

$$\overrightarrow{P'_{ij}P'_{i,j+1}} = \overrightarrow{k} \times (\overrightarrow{P_{ij}P_{i,j+1}} \times \overrightarrow{k}),$$

Hence, the turning angle is computed as:

$$\phi_{ij} = \arctan\left(\frac{\left\|\overrightarrow{P'_{ij}P'_{i,j+1}} \times \overrightarrow{P'_{i,j+1}P'_{i,j+2}}\right\|}{\overrightarrow{P'_{ij}P'_{i,j+1}} . \overrightarrow{P'_{i,j+1}P'_{i,j+2}}}\right).$$

The climbing angle,ψ_{ij},is the angle between the path segment $\overrightarrow{P_{ij}P_{i,i+1}}$ and its projection $\overrightarrow{P'_{ii}P'_{i,i+1}}$ onto the horizontal plane.

It is givenby:

$$\psi_{ij} = \arctan\left(\frac{z_{i,j+1} - z_{ij}}{\left\|\overrightarrow{P'_{ij}P'_{i,j+1}}\right\|}\right).$$

The smoothness cost can be expressed as:

$$F_4(X_i) = a_1 \sum_{j=1}^{n-2} \phi_{ij} + a_2 \sum_{j=1}^{n-1} \mid \psi_{ij} - \psi_{i,j-1} \mid,$$

where a_1 and a_2 denote the penalty coefficients for the turning and climbing angles, respectively.

Taking into account the optimality, safety, and feasibility constraints related to a path X_i, the comprehensive cost function can be formulated as:

$$F(X_i) = \sum_{k=1}^{4} b_k F_k(X_i),$$

where b_k represents the weight coefficients, and $F_1(X_\mathrm{i})$ through $F_4(X_\mathrm{i})$ correspond to the costs associated with path length (1), threat (2), smoothness (4), and flight height (8), respectively. The decision variable X_i encompasses a list of n waypoints $P_{ij} = (x_{ij}, y_{ij}, z_{ij})$ where P_{ij} is an element of O, the operational space for UAVs. With these definitions, the cost function F is completely specified and can be utilized in the path planning procedure.

Corrugated Bulkhead Design

The objective of this problem is to reduce the weight of a corrugated bulkhead within a chemical tanker. Thus, the dimensions of a single corrugation—plate thickness (t_p), web width (c), depth of corrugation (d), and number of corrugations (N_c)—are considered design variables, as shown in **SI Figure 2**.

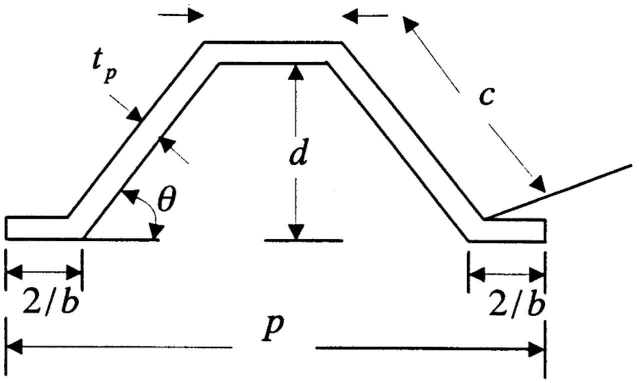

SI Figure 2: Configuration of a single corrugation.

The mathematical formulation of this optimization challenge is as follows, where x_1 represents width, x_2 represents depth, x_3 represents length, and x_4 represents plate thickness[1]:

$$f(X) = \frac{5.885x_4(x_1 + x_3)}{x_1 + \sqrt{|x_3^2 - x_2^2|}},$$

subject to:

$$g_1(X) = -x_4x_2\left(0.4x_1 + \frac{x_3}{6}\right) + 8.94\left(x_1 + \sqrt{|x_3^2 - x_2^2|}\right) \leq 0,$$

$$g_2(X) = -x_4x_2^2\left(0.2x_1 + \frac{x_3}{12}\right) + 2.2\left[8.94\left(x_1 + \sqrt{|x_3^2 - x_2^2|}\right)\right]^{\frac{4}{3}} \leq 0,$$

$$g_3(X) = -x_4 + 0.0156x_1 + 0.15 \leq 0,$$

$$g_4(X) = -x_4 + 0.0156x_3 + 0.15 \leq 0,$$

$$g_5(X) = -x_4 + 1.05 \leq 0,$$

$$g_6(X) = -x_3 + x_2 \leq 0,$$

variable range:

$$0 \leq x_1, x_2, x_3 \leq 100,$$

$$0 \leq x_4 \leq 5.$$

Multiple Disk Clutch Brake Design

This task focuses on reducing the weight of a multiple disc clutch brake by evaluating five discrete design parameters: inner radius, outer radius, disc thickness, actuating force, and the number of friction surfaces. Eight different constraints are considered based on geometric and operational conditions.

The feasible region encompasses almost 70% of the search area. The optimal solution for this task is $f(X) = 0.313656611$ at $X = \{70, 90, 1, 810, 3\}$ with one constraint being active[2].

$$
\begin{aligned}
&f(x) = \Pi(r_o^2 - r_i^2)t(Z+1)\rho \\
&\text{subject to:} \\
&g_1(x) = r_o - r_i - \Delta r \geq 0 \\
&g_2(x) = l_{\max} - (Z+1)(t+\delta) \geq 0 \\
&g_3(x) = P_{\max} - P_{rz} \geq 0 \\
&g_4(x) = P_{\max} v_{sr\,\max} - P_{rz}\nu_{sr} \geq 0 \\
&g_5(x) = v_{sr\max} - v_{sr} \geq 0 \\
&g_6 = T_{\max} - T \geq 0 \\
&g_7(x) = M_h - sM_s \geq 0 \\
&g_8(x) = T \geq 0 \\
&\text{where,} \\
&M_h = \frac{2}{3}\mu F Z \frac{r_o^3 - r_i^2}{r_o^2 - r_i^3}, P_{rz} = \frac{F}{\Pi(r_o^2 - r_i^2)}, \\
&v_{rz} = \frac{2\Pi n(r_o^3 - r_i^3)}{90\,(r_o^2 - r_i^2)}, T = \frac{I_z \Pi n}{30(M_h + M_f)} \\
&\Delta r = 20\text{mm}, I_z = 55\text{kgmm}^2, P_{\max} = 1\text{MPa}, F_{\max} = 1000\text{N}, \\
&T_{\max} = 15s, \mu = 0.5, s = 1.5, M_s = 40\text{Nm}, \\
&M_f = 3\text{Nm}, n = 250\text{rpm}, \\
&v_{sr\,\max} = 10\text{m/s}, l_{\max} = 30\text{mm}, r_{i\,\min} = 60, \\
&r_{i\,\max} = 80, r_{o\,\min} = 90, \\
&r_{o\,\max} = 110, t_{\min} = 1.5, t_{\max} = 3, F_{\min} = 600, \\
&F_{\max} = 1000, Z_{\min} = 2, Z_{\max} = 9,
\end{aligned}
$$

Blending-Pooling-Separation Problem

This challenge involves a feed mixture comprising three components, which are processed to produce two multi-component outputs through the use of separators, as well as splitting, blending, and pooling operations. The operating cost for each separator is directly proportional to its flow rate, with constraints dictated by the mass balance relationships surrounding each individual separator, splitter, and mixer[3].

$$
\begin{aligned}
&f(\overline{\alpha}) = 0.9979 + 0.00432\alpha_5 + 0.01517\alpha_{13} \\
&\text{subject to:} \\
&h_1(\overline{\alpha}) = \alpha_4 + \alpha_3 + \alpha_2 + \alpha_1 = 300, \\
&h_2(\overline{\alpha}) = \alpha_6 - \alpha_8 - \alpha_7 = 0, \\
&h_3(\overline{\alpha}) = \alpha_9 - \alpha_{11} - \alpha_{10} - \alpha_{12} = 0,
\end{aligned}
$$

$$h_4(\overline{\alpha}) = \alpha_{14} - \alpha_{16} - \alpha_{17} - \alpha_{15} = 0,$$
$$h_5(\overline{\alpha}) = \alpha_{18} - \alpha_{20} - \alpha_{19} = 0,$$
$$h_6(\overline{\alpha}) = \alpha_5\alpha_{21} - \alpha_6\alpha_{22} - \alpha_9\alpha_{23} = 0,$$
$$h_7(\overline{\alpha}) = \alpha_5\alpha_{24} - \alpha_6\alpha_{25} - \alpha_9\alpha_{26} = 0,$$
$$h_8(\overline{\alpha}) = \alpha_5\alpha_{27} - \alpha_6\alpha_{28} - \alpha_9\alpha_{29} = 0,$$
$$h_9(\overline{\alpha}) = \alpha_{13}\alpha_{30} - \alpha_{14}\alpha_{31} - \alpha_{18}\alpha_{32} = 0,$$
$$h_{10}(\overline{\alpha}) = \alpha_{13}\alpha_{33} - \alpha_{14}\alpha_{34} - \alpha_{18}\alpha_{35} = 0,$$
$$h_{11}(\overline{\alpha}) = \alpha_{13}\alpha_{36} - \alpha_{14}\alpha_{37} - \alpha_{18}\alpha_{35} = 0,$$
$$h_{12}(\overline{\alpha}) = 0.333\alpha_1 + \alpha_{15}\alpha_{31} - \alpha_5\alpha_{21} = 0,$$
$$h_{13}(\overline{\alpha}) = 0.333\alpha_1 + \alpha_{15}\alpha_{34} - \alpha_5\alpha_{24} = 0,$$
$$h_{14}(\overline{\alpha}) = 0.333\alpha_1 + \alpha_{15}\alpha_{37} - \alpha_5\alpha_{27} = 0,$$
$$h_{15}(\overline{\alpha}) = 0.333\alpha_2 + \alpha_{10}\alpha_{23} - \alpha_{13}\alpha_{30} = 0,$$
$$h_{16}(\overline{\alpha}) = 0.333\alpha_2 + \alpha_{10}\alpha_{26} - \alpha_{13}\alpha_{33} = 0,$$
$$h_{17}(\overline{\alpha}) = 0.333\alpha_2 + \alpha_{10}\alpha_{29} - \alpha_{13}\alpha_{36} = 0,$$
$$h_{18}(\overline{\alpha}) = 0.333\alpha_3 + \alpha_7\alpha_{22} + \alpha_{11}\alpha_{23} + \alpha_{16}\alpha_{31} + \alpha_{19}\alpha_{32} = 30,$$
$$h_{19}(\overline{\alpha}) = 0.333\alpha_3 + \alpha_7\alpha_{25} + \alpha_{11}\alpha_{26} + \alpha_{16}\alpha_{34} + \alpha_{19}\alpha_{35} = 50,$$
$$h_{20}(\overline{\alpha}) = 0.333\alpha_3 + \alpha_7\alpha_{28} + \alpha_{11}\alpha_{29} + \alpha_{16}\alpha_{37} + \alpha_{19}\alpha_{38} = 30,$$
$$h_{21}(\overline{\alpha}) = \alpha_{21} + \alpha_{24} + \alpha_{27} = 1,$$
$$h_{22}(\overline{\alpha}) = \alpha_{22} + \alpha_{25} + \alpha_{28} = 1,$$
$$h_{23}(\overline{\alpha}) = \alpha_{23} + \alpha_{26} + \alpha_{29} = 1,$$
$$h_{24}(\overline{\alpha}) = \alpha_{30} + \alpha_{33} + \alpha_{36} = 1,$$
$$h_{25}(\overline{\alpha}) = \alpha_{31} + \alpha_{34} + \alpha_{37} = 1,$$
$$h_{26}(\overline{\alpha}) = \alpha_{32} + \alpha_{35} + \alpha_{38} = 1,$$
$$h_{27}(\overline{\alpha}) = \alpha_{25} = 0,$$
$$h_{28}(\overline{\alpha}) = \alpha_{28} = 0,$$
$$h_{29}(\overline{\alpha}) = \alpha_{23} = 0,$$
$$h_{30}(\overline{\alpha}) = \alpha_{37} = 0,$$
$$h_{31}(\overline{\alpha}) = \alpha_{32} = 0,$$
$$h_{32}(\overline{\alpha}) = \alpha_{35} = 0,$$

with bounds :

$$0 \leq \alpha_1, \alpha_3, \alpha_8, \alpha_9, \alpha_5, \alpha_6, \alpha_{14}, \alpha_{18}, \alpha_{10}, \alpha_{16}, \alpha_{13}, \alpha_{20} \leq 90,$$
$$0 \leq \alpha_2, \alpha_4, \alpha_7, \alpha_{11}, \alpha_{12}, \alpha_{15}, \alpha_{17}, \alpha_{19} \leq 150,$$
$$0 \leq \alpha_{21}, \alpha_{23}, \alpha_{24}, \alpha_{25}, \alpha_{27}, \alpha_{28} \leq 1,$$
$$0 \leq \alpha_{22}, \alpha_{32}, \alpha_{34}, \alpha_{35}, \alpha_{37}, \alpha_{38} \leq 1.2,$$
$$0 \leq \alpha_{26}, \alpha_{29}, \alpha_{30}, \alpha_{31}, \alpha_{33}, \alpha_{36} \leq 0.5.$$

Industrial Refrigeration System

The system comprises three primary vessels as depicted in **SI Figure 3**. Firstly, the high-pressure receiver stores liquid refrigerant draining from the condenser. This liquid refrigerant is then throttled from the high-pressure receiver either to the intermediate pressure receiver or directly to the evaporators in the banana and tomato ripening rooms. A back-pressure regulator is used to maintain the refrigerant temperature in the evaporators of the banana/tomato rooms at a desired level. Subsequently, the backpressure regulator throttles the refrigerant gas to the intermediate pressure receiver, which operates at a lower temperature and pressure. The intermediate pressure receiver then either pumps the liquid to the cooler and cooler dock evaporators or further throttles it to the low-pressure receiver. From the low-pressure receiver, liquid refrigerant is pumped to the freezer evaporators using a mechanical liquid recirculating pump. The liquid levels in both the intermediate and low-pressure receivers are kept nearly constant by a pilot-operated, modulating expansion valve controlled by a float switch located on the receiver tank. This issue is addressed as a non-linear inequality Constrained Optimization Problem (COP) and is represented in the following manner[4]:

$$\begin{aligned}
f(\overline{\alpha}) = {} & 63098.88\alpha_2\alpha_4\alpha_{12} + 5441.5\alpha_2^2\alpha_{12} + 115055.5\alpha_2^{1.664}\alpha_6 \\
& + 6172.27\alpha_2^2\alpha_6 + 63098.88\alpha_1\alpha_3\alpha_{11} + 5441.5\alpha_1^2\alpha_{11} \\
& + 115055.5\alpha_1^{1.664}\alpha_5 + 6172.27\alpha_1^2\alpha_5 + 140.53\alpha_1\alpha_{11} \\
& + 281.29\alpha_3\alpha_{11} + 70.26\alpha_1^2 + 281.29\alpha_1\alpha_3 + 281.29\alpha_3^2 \\
& + 14437\alpha_8^{1.8812}\alpha_{12}^{0.3424}\alpha_{10}\alpha_{14}^{-1}\alpha_1^2\alpha_7\alpha_9^{-1} \\
& + 20470.2\alpha_7^{2.893}\alpha_{11}^{0.316}\alpha_1^2
\end{aligned}$$

subject to:

$$\begin{aligned}
& g_1(\overline{\alpha}) = 1.524\alpha_7^{-1} \leq 1, \\
& g_2(\overline{\alpha}) = 1.524\alpha_8^{-1} \leq 1, \\
& g_3(\overline{\alpha}) = 0.07789\alpha_1 - 2\alpha_7^{-1}\alpha_9 - 1 \leq 0, \\
& g_4(\overline{\alpha}) = 7.05305\alpha_9^{-1}\alpha_1^2\alpha_{10}\alpha_8^{-1}\alpha_2^{-1}\alpha_{14}^{-1} - 1 \leq 0, \\
& g_5(\overline{\alpha}) = 0.0833\alpha_{13}^{-1}\alpha_{14} - 1 \leq 0, \\
& g_6(\overline{\alpha}) = 47.136\alpha_2^{0.333}\alpha_{10}^{-1}\alpha_{12} - 1.333\alpha_8\alpha_{13}^{2.1195} \\
& \quad + 62.08\alpha_{13}^{2.1195}\alpha_{12}^{-1}\alpha_8^{0.2}\alpha_{10}^{-1} - 1 \leq 0, \\
& g_7(\overline{\alpha}) = 0.04771\alpha_{10}\alpha_8^{1.8812}\alpha_{12}^{0.3424} - 1 \leq 0, \\
& g_8(\overline{\alpha}) = 0.0488\alpha_9\alpha_7^{1.893}\alpha_{11}^{0.316} - 1 \leq 0, \\
& g_9(\overline{\alpha}) = 0.0099\alpha_1\alpha_3^{-1} - 1 \leq 0, \\
& g_{10}(\overline{\alpha}) = 0.0193\alpha_2\alpha_4^{-1} - 1 \leq 0, \\
& g_{11}(\overline{\alpha}) = 0.0298\alpha_1\alpha_5^{-1} - 1 \leq 0, \\
& g_{12}(\overline{\alpha}) = 0.056\alpha_2\alpha_6^{-1} - 1 \leq 0, \\
& g_{13}(\overline{\alpha}) = 2\alpha_9^{-1} - 1 \leq 0, \\
& g_{14}(\overline{\alpha}) = 2\alpha_{10}^{-1} - 1 \leq 0, \\
& g_{15}(\overline{\alpha}) = \alpha_{12}\alpha_{11}^{-1} - 1 \leq 0,
\end{aligned}$$

with bounds:

$$0.001 \leq \alpha_i \leq 5, \quad i = 1, \ldots, 14.$$

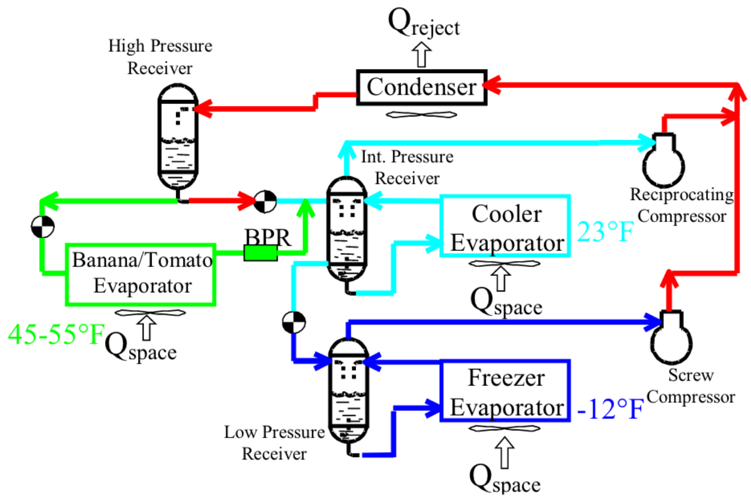

SI Figure 3: Simplified System Diagram

Himmelblau's Function

The problem was proposed by Proctor and Gamble Corporation to simulate Process Design Problems and has been referenced by D. M. Himmelblau in Ref. It serves as a widely recognized benchmark for evaluating non-linear constrained optimization algorithms. This specific problem includes six nonlinear constraints and five variables. The detailed description is provided below[5]:

$$\mathrm{f}(\overline{\alpha}) = 5.3578547\alpha_3^2 + 0.8356891\alpha_1\alpha_5 + 37.293239\alpha_1 - 40792.141$$

subject to:

$$g_1(\overline{\alpha}) = -G1 \leq 0,$$

$$-g_2(\overline{\alpha}) = G1 - 92 \leq 0,$$

$$g_3(\overline{\alpha}) = 90 - G2 \leq 0,$$

$$-g_4(\overline{\alpha}) = G2 - 110 \leq 0,$$

$$-g_5(\overline{\alpha}) = 20 - G3 \leq 0,$$

$$g_6(\overline{\alpha}) = G3 - 25 \leq 0,$$

where,

$$G1 = 85.334407 + 0.0056858\alpha_2\alpha_5 + 0.0006262\alpha_1\alpha_4 - 0.0022053\alpha_3\alpha_5,$$

$$G2 = 80.51249 + 0.0071317\alpha_2\alpha_5 + 0.0029955\alpha_1\alpha_2 + 0.0021813\alpha_3^2,$$

$$G3 = 9.300961 + 0.0047026\alpha_3\alpha_5 + 0.00125447\alpha_1\alpha_3 + 0.0019085\alpha_3\alpha_4,$$

with bounds

$$78 \leq \alpha_1 \leq 102,$$

$$33 \leq \alpha_2 \leq 45,$$

$$27 \leq \alpha_3 \leq 45,$$
$$27 \leq \alpha_4 \leq 45.$$

0.0.1. Cantilever Beam

Based on earlier research , the cantilever beam depicted in **SI Figure 4** is analyzed in its deformed state under an applied end force F and an end moment M_0 . The end force is split into its horizontal component F_x and vertical component F_y . The moment exerted at any position (x, y) along the beam can be described as follows:

$$M_{(x,y)} = EI\frac{d\alpha}{ds} = F_x(b - y) + F_y(a - x) + M_0,$$

The coordinates (a,b) represent the position of the beam's deflected endpoint. The beam's flexural rigidity, denoted by EI, is considered to be uniform along the entire beam length. The slope at any given point (x,y) is represented by α, and the distance from the fixed end to that point along the beam is denoted by s. It is assumed that the total length L of the undeformed beam remains constant after deformation. By differentiating Equation (1) and then substitutin:

$$\frac{dx}{ds} = \cos\alpha \quad \text{and} \quad \frac{dy}{ds} = \sin\alpha$$

yields

$$\frac{d^2\alpha}{ds^2} = -\frac{F}{EI}(\cos\alpha + \sin\alpha).$$

In order to address the aforementioned second-order differential equation, it is necessary to employ the following two boundary conditions[6].

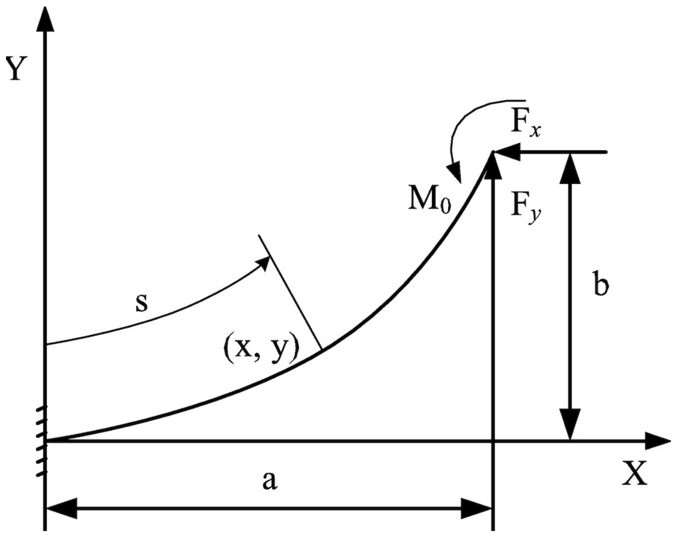

SI Figure 4: Simplified System Diagram

References

[1] X. Wang, H. Wang, S. Li, H. Jin, A reinforcement learning-based online learning strategy for real-time short-term load forecasting, Energy 305 (2024) 132344. doi:10.1016/j.energy.2024.132344.

[2] B. Yang, X. Liang, J. Zhong, J. Peng, G. Wang, L. Lin, Unifying Dynamic Optimizer Search and Network Architecture Search, in: 2021 IEEE International Conference on Multimedia and Expo (ICME), IEEE, Shenzhen, China, 2021, pp. 1–6. doi:10.1109/ICME51207.2021.9428169.

[3] K. Wang, M. Guo, C. Dai, Z. Li, Information-decision searching algorithm: Theory and applications for solving engineering optimization problems, Information Sciences 607 (2022) 1465–1531. doi:10.1016/j.ins.2022.06.008.

[4] A. A. Heidari, S. Mirjalili, H. Faris, I. Aljarah, M. Mafarja, H. Chen, Harris hawks optimization: Algorithm and applications, Future Generation Computer Systems 97 (2019) 849–872. doi:10.1016/j.future.2019.02.028.

[5] A. Kumar, G. Wu, M. Z. Ali, R. Mallipeddi, P. N. Suganthan, S. Das, A test-suite of non-convex constrained optimization problems from the real-world and some baseline results, Swarm and Evolutionary Computation 56 (2020) 100693. doi:10.1016/j.swevo.2020.100693.

[6] D.-E. Ko, S.-H. Shin, A Study on the Optimum Design of Corrugated Bulkhead for Product Carrier, IOP Conference Series: Materials Science and Engineering 269 (2017) 012084. doi:10.1088/1757-899X/269/1/012084.
